# Causal link between gut microbiota and four types of pancreatitis: a genetic association and bidirectional Mendelian randomization study

**DOI:** 10.3389/fmicb.2023.1290202

**Published:** 2023-11-23

**Authors:** Kui Wang, Xianzheng Qin, Taojing Ran, Yundi Pan, Yu Hong, Jiawei Wang, Xianda Zhang, XiaoNan Shen, Chenxiao Liu, Xinchen Lu, Yifei Chen, Yaya Bai, Yao Zhang, Chunhua Zhou, Duowu Zou

**Affiliations:** ^1^Department of Gastroenterology, Ruijin Hospital, School of Medicine, Shanghai Jiao Tong University, Shanghai, China; ^2^Department of Gastroenterology, The Affiliated Hospital of Kunming University of Science and Technology, The First People’s Hospital of Yunnan Province, Kunming, China

**Keywords:** pancreatitis, genetics, gut microbiota, bidirectional Mendelian randomization, single nucleotide polymorphisms

## Abstract

**Background:**

A number of recent observational studies have indicated a correlation between the constitution of gut microbiota and the incidence of pancreatitis. Notwithstanding, observational studies are unreliable for inferring causality because of their susceptibility to confounding, bias, and reverse causality, the causal relationship between specific gut microbiota and pancreatitis is still unclear. Therefore, our study aimed to investigate the causal relationship between gut microbiota and four types of pancreatitis.

**Methods:**

An investigative undertaking encompassing a genome-wide association study (GWAS) comprising 18,340 participants was undertaken with the aim of discerning genetic instrumental variables that exhibit associations with gut microbiota, The aggregated statistical data pertaining to acute pancreatitis (AP), alcohol-induced AP (AAP), chronic pancreatitis (CP), and alcohol-induced CP (ACP) were acquired from the FinnGen Consortium. The two-sample bidirectional Mendelian randomization (MR) approach was utilized. Utilizing the Inverse-Variance Weighted (IVW) technique as the cornerstone of our primary analysis. The Bonferroni analysis was used to correct for multiple testing, In addition, a number of sensitivity analysis methodologies, comprising the MR-Egger intercept test, the Cochran’s Q test, MR polymorphism residual and outlier (MR-PRESSO) test, and the leave-one-out test, were performed to evaluate the robustness of our findings.

**Results:**

A total of 28 intestinal microflora were ascertained to exhibit significant associations with diverse outcomes of pancreatitis. Among them, *Class Melainabacteria* (OR = 1.801, 95% CI: 1.288–2.519, *p* = 0.008) has a strong causality with ACP after the Bonferroni-corrected test, in order to assess potential reverse causation effects, we used four types of pancreatitis as the exposure variable and scrutinized its impact on gut microbiota as the outcome variable, this analysis revealed associations between pancreatitis and 30 distinct types of gut microflora. The implementation of Cochran’s Q test revealed a lack of substantial heterogeneity among the various single nucleotide polymorphisms (SNP).

**Conclusion:**

Our first systematic Mendelian randomization analysis provides evidence that multiple gut microbiota taxa may be causally associated with four types of pancreatitis disease. This discovery may contribute significant biomarkers conducive to the preliminary, non-invasive identification of Pancreatitis. Additionally, it could present viable targets for potential therapeutic interventions in the disease’s treatment.

## Background

Pancreatitis represents a multifaceted, protracted, debilitative inflammatory disorder affecting the pancreas, encompassing clinical designations such as acute pancreatitis (AP), recurrent acute pancreatitis (RAP), and chronic pancreatitis (CP). Notably, AP emerges as the principal etiology behind hospitalizations associated with gastrointestinal conditions, and its prevalence is increasing globally (Iannuzzi et al., 2022; Vege and Chari, 2022; Ben-Aharon et al., 2023). AP is linked with significant morbidity and mortality, and the expenses incurred during hospitalization in the United States amount to over $30,000 per individual (Xiao et al., 2016; Petrov and Yadav, 2019). Prolonged occurrences of AP can ultimately culminate in pancreatic insufficiency and the development of CP (Cohen and Kent, 2023). CP represents a persistent inflammatory and fibrotic condition affecting the pancreas, with an incidence ranging from 42 to 73 per 100,000 adults in the United States (Beyer et al., 2020). Apart from gallstone-induced conditions, alcohol abuse stands as the predominant etiological factor for acute pancreatitis and represents the primary cause of chronic pancreatitis in the human population (Yadav and Whitcomb, 2010; Lugea et al., 2017; Bhatia et al., 2022; Nagy et al., 2022; Ocskay et al., 2022; Szentesi et al., 2022). In the United States, alcohol is identified as the primary secondary etiological agent for AP, attributing to nearly one-third of AP cases (Argueta et al., 2021). Utilizing the 2016 National Readmission Database (NRD), Nieto et al. (2023) discerned that a significant proportion of the 43.1% 11-month readmission rate was attributable to recurrent episodes of Alcohol -induced acute pancreatitis (AAP). Persons diagnosed with alcohol-induced chronic pancreatitis (ACP) characteristically exhibit a documented history of significant alcohol intake (Kleeff et al., 2017). The initiation age is influenced by the intensity of alcohol overindulgence; however, indications of chronic pancreatitis commonly manifest by the age of 40 years. These clinical manifestations encompass pronounced discomfort, steatorrhea, reduction in weight, and eventual cachexia (Cohen and Kent, 2023; de Rijk et al., 2023; Klöppel and Zamboni, 2023). Consequently, prevention and effectiveness of management are priorities in clinical practice. In recent times, the gut microbiota has garnered substantial attention as a promising therapeutic focus for averting or managing chronic ailments and fostering human longevity and health span extension (Thomas and Jobin, 2020; Shah et al., 2022; Shanahan et al., 2023). The human intestinal microbiota is a symbiotic organ of microorganisms located in the gut that is involved in important metabolic and immune processes such as host immunity, dietary digestion, intestinal hormonal function, and intestinal osmosis (Bevins and Salzman, 2011; Sun et al., 2015; Lynch and Pedersen, 2016; de Goffau et al., 2019). Growing evidence suggests that microbial dysbiosis may play an important role in the pathogenesis of pancreatitis (Farrell et al., 2012; Hamada et al., 2018; Nishiyama et al., 2018; Del Castillo et al., 2019; Fernández-Millán and Guillén, 2022; Zhang et al., 2022; Sun et al., 2023). 0.16S qPCR has revealed a scarcity of bacteria in normal mice pancreas compared to Kras-mutant or Trp53-mutant mice (Pushalkar et al., 2018). Numerous fecal microbiota, such as *Staphylococcus, Enterococcus, Bifidobacterium, Lachnoclostridium, Escherichia coli, Faecalibacterium prausnitzii, Actinobacteria*, and *Ruminococcus bromii*, have been linked to pancreatitis based on findings from observational studies (Beger et al., 1986; Zhang et al., 2018; Zhou et al., 2020; Jing et al., 2022; Xu et al., 2023). Jandhyala et al. (2017) conducted an inquiry into the enduring functional consequences of altered intestinal microbiota in individuals diagnosed with CP. Nevertheless, observational studies are unreliable for inferring causality because of their susceptibility to confounding, bias, and reverse causality. To sum up, the causal connections between the gut microbiota and pancreatitis, as well as the directionality of such associations, remain equivocal. Therefore, it is imperative to undertake comprehensive investigations to elucidate the causal relationship between the gut microbiota and pancreatitis.

Over the last decade, Genome-wide association studies (GWASs) have scrutinized a vast number of genetic variants spanning the genomes of numerous individuals to discern associations between genotypes and phenotypes (Sud et al., 2017; Visscher et al., 2017). Mendelian randomization (MR) represents an analytical approach that leverages germline genetic variants as instrumental variables (IVs) to explore and infer causality for various exposures (Smith and Ebrahim, 2003; Davey Smith and Hemani, 2014; Sekula et al., 2016; Su et al., 2023). These genetic variants are stochastically distributed across the populace during meiosis and conception, simulating a controlled randomized experimental context. The MR framework is adept at mitigating the influence of potential residual confounders and circumventing the pitfalls of reverse causation bias (Zhao et al., 2023). The domain of gut microbiota and disease GWASs has undergone rapid expansion (Wang et al., 2018; Kurilshikov et al., 2021; Au Yeung and Gill, 2023).

In this investigation, we sought to elucidate the causal connection between gut microbiota and a diverse spectrum of pancreatitis conditions through a comprehensive bidirectional two-sample MR analysis. The data for four distinct pancreatitis types were sourced from the FinnGen Consortium including CP, AP, ACP, and AAP. Through the implementation of a bi-directional MR strategy, we can probe the potential causal influence of gut microbiota on pancreatitis risk, as well as ascertain whether the genetic predisposition to pancreatitis risk exerts a causal impact on the gut microbiota. Based on these findings, we sought to elucidate the role of the gut microbiota in the pathogenesis of pancreatitis, which may also provide viable targets for potential therapeutic interventions for the disease, such as probiotic therapies, dietary modifications, and fecal microbiota transplantation (FMT) (Mederos et al., 2021; Petrov and Taylor, 2022; Strand et al., 2022; Petrov, 2023).

## Materials and methods

### Study design

**FIGURE 1:**
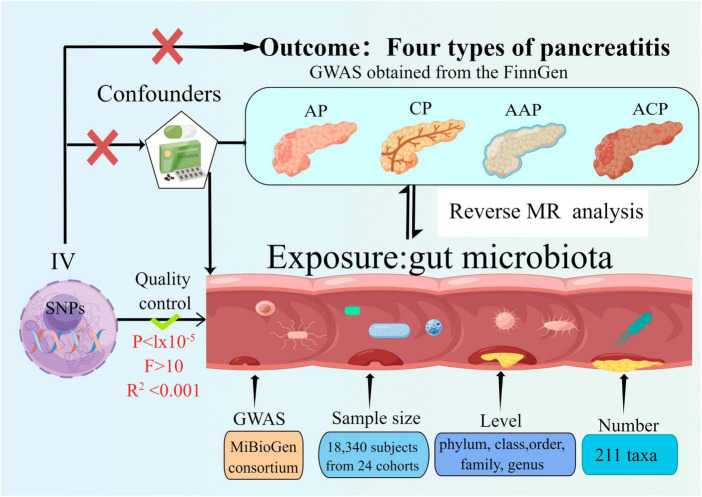
Study design of the two-sample bidirectional Mendelian randomization for the effect of the genetically predicted gut microbiome on four types of pancreatitis. SNP, single nucleotide polymorphism; AP, acute pancreatitis; CP, chronic pancreatitis; AAP, alcohol-induced AP; ACP, alcohol-induced CP; IV, instrumental variables.

The bidirectional MR study was conducted to assess the causal associations between gut microbiota and pancreatitis. Figure 1 shows the overall flow of this study. Two-sample MR is required to perform three key assumptions (Bowden and Holmes, 2019), (1) IVs are substantially correlated with gut microbiota; (2) IVs are not affected by confounding variables other than gut microbiota; (3) IVs can only have an effect on pancreatitis through the gut microbiota; We followed the strengthening the reporting of observational studies in epidemiology using MR (STROBE-MR) guidelines in reporting our results (Skrivankova et al., 2021a,b; Au Yeung and Gill, 2023).

### Exposure data

Single nucleotide polymorphisms (SNPs) linked to the composition of the human gut microbiome were discerned as IVs from a GWAS Genome Data Set sourced from the international MiBioGen Alliance (Kurilshikov et al., 2021). The dataset pertaining to the gut microbiome encompassed 211 taxa with a mean abundance exceeding 1%, which included 131 genera, 35 families, 20 orders, 16 classes, and 9 phyla. After the exclusion of 15 taxa associated with unidentified groups (comprising 12 genera and 3 families), 196 bacterial taxa were incorporated into the MR evaluation (Kurilshikov et al., 2021).

### Outcome data

The FinnGen research project encompasses the acquisition and scrutiny of genetic information from upwards of 500,000 individuals affiliated with Finnish biobanks. This data is integrated with their electronic health records from the Care Register for Health Care, as well as data derived from the registries of cancer, mortality causes, and medication reimbursements. Summary GWAS statistics for AP, AAP, CP, and ACP downloaded from FinnGen. The FinnGen consortium data was utilized in its R5 release^1^, comprehensive data regarding each incorporated outcome, along with their correlated GWAS details, are elaborated upon in the link. There are 457 cases and 218,335 controls for AAP^2^, 195,144 controls and 3,022 cases for AP in this data set^3^, 977 cases and 217,815 controls for ACP^4^ and 1,737 cases and 195,144 controls with CP^5^. The genetic linkages were calibrated taking into account variables such as sex, age, genetic constituents, and genotyping batch variations.

### Instrumental variable selection

An individual taxon was defined as a feature of bacteria at five levels (phylum, class, order, family, and genus). As a quality control measure, the following quality control measures were applied to ensure the credibility and precision of the conclusions regarding the causal link between the gut microbiota and pancreatitis risk. The number of IVs obtained under the strict threshold (*P* < 5 × 10^–8^) was extremely low, so we used the more inclusive threshold (*P* < 1 × 10^–5^) to obtain relatively more IVs (Kurilshikov et al., 2021; Zeng et al., 2023). Moreover, to ensure the independence of each IV, SNPs within a genomic window of 10,000 kilobases were subjected to pruning, applying a threshold of *r*^2^ < 0.001 to mitigate the effects of linkage disequilibrium (LD). Then, palindromic SNPs and SNPs not present in the result were deleted from the IVs. Ensuring that the effects of SNPs on exposure match the effects on outcome on the same allele is an essential step in MR. We removed palindromic SNPs to avoid distorting strand orientation or encoding alleles. Alleles were aligned to the human genome reference sequence (build 37) and ambiguous and duplicated SNPs were deleted during the harmonization process. In order to mitigate the risk of SNPs being linked to potential confounding variables or risk factors (such as diabetes and cholestasis etc.) that could influence the outcomes, the Phenoscanner tool was employed to scrutinize and eliminate such associations. The F-statistic was determined for one SNP at a time, and SNPs with an F-statistic less than 10 were discarded if present, to avoid potential instrumental bias (Burgess and Thompson, 2011). We conducted an assessment of the strength of each individual IV via the F-statistics, denoted as *F* = β*^2^ exposure/SE^2^ exposure*. Additionally, an aggregate F-statistic was determined employing the subsequent formula: *F*= *(n–k–1)^2^/(1–R^2^)*. In which n represents the sample size of the exposure dataset, *k* denotes the count of SNPs, and *R*^2^ signifies the fraction of exposure variance elucidated by genetic factors. The value of *R*^2^ was ascertained utilizing the subsequent equation: *R*^2^ = 2 × EAF × (1–EAF) × beta^2^/2 × EAF × (1–EAF) × beta^2^ + 2 × EAF × (1–EAF) × *n* × se^2^ (Pierce et al., 2011). Multiple approaches were employed to assess potential horizontal pleiotropy. Specifically, the presence of horizontal pleiotropy was monitored using the *p*-value derived from the MR-Egger intercept test and the MR pleiotropy residual sum and outlier (MR-PRESSO) global test (Yavorska and Burgess, 2017; Verbanck et al., 2018). A significance level of *P* < 0.05 was considered to indicate statistical significance. SNPs were arranged in ascending order based on their MR-PRESSO outlier test p-values and were then removed one by one (Mi et al., 2022).

### MR analysis

We utilized MR analysis to evaluate the causal association between microbiome characteristics and pancreatitis. For characteristics that only contained one IV, the Wald ratio test was used to estimate the association between the identified IV and each pancreatitis (Burgess et al., 2017). Five popular MR methods were applied to features with multiple IVs: inverse variance weighted (IVW) test (Burgess et al., 2013), MR-PRESSO (Bowden et al., 2017; Verbanck et al., 2018), weighted median estimator (WME) (Bowden et al., 2016), MR-Egger regression, and weighted mode. A combination of IVs will be analyzed primarily using the IVW method, with the other four being used as supplements (Bowden et al., 2016).

In particular, in order to adjust our results for multiple hypotheses, we used the Bonferroni type and the Hochberg false discovery rate (FDR), The threshold for statistical significance was determined as *P* < 0.05 divided by the effective number of distinct bacterial taxa at the corresponding taxonomic level (denoted as n). A significant association was observed when the Bonferroni-corrected *p*-value was less than 0.05, whereas suggestive evidence of association was found when the *p*-value was less than 0.05 but the Bonferroni-corrected *P*-value was greater than 0.05 (Glickman et al., 2014; Korthauer et al., 2019).

### Reverse MR analysis

To investigate whether pancreatitis have any causal effect on the bacterial genera, we also analyzed a reverse MR (i.e., pancreatitis as the exposure and the gut microbiota as the outcome) using SNPs associated with pancreatitis as IVs.

### Heterogeneity

On significant findings, sensitivity analyses were conducted in order to detect possible heterogeneities and pleiotropies. As a means of detecting heterogeneity, we used the Cochran’s Q test. In order to determine horizontal pleiotropy, we used the MR-Egger intercept test. As long as the *P*-value was greater than 0.05, we defined there to be no heterogeneity or pleiotropy. Furthermore, a leave-one-out sensitivity analysis was performed on the significant findings identified, aiming to discern if the causal associations observed in the TSMR analysis were primarily influenced by any individual SNP. In all analyses, R (version 4.2.2) was used in conjunction with TwoSample MR (version 0.5.6) and stats (version 4.2.1) (Yavorska and Burgess, 2017).

## Results

### An overview of IVs in taxa

A genome-wide significance threshold screening (*P* < 1 × 10^–5^), likelihood distribution tests, harmonization, and verification of F statistics have identified multiple SNPs as IVs in 196 bacterial taxa. In all maintained SNPs, the F-statistic is greater than 10, (Supplementary Table 1) indicating sufficient power of correlation between the IVs and the associated bacterial taxon. Consequently, there is no weak instrumental bias in our study. Finally, having excluded pleiotropic SNPs identified by the MR-PRESSO outlier test and the MR-Egger regression, IVs were not horizontally pleiotropic (*P* > 0.05 for MR-PRESSO global test and *P* > 0.05 for MR-Egger regression, Supplementary Tables 1–8).

### Causative effects of gut microbiota on the development of four types of pancreatitis

#### Gut microbiome and AP

**FIGURE 2:**
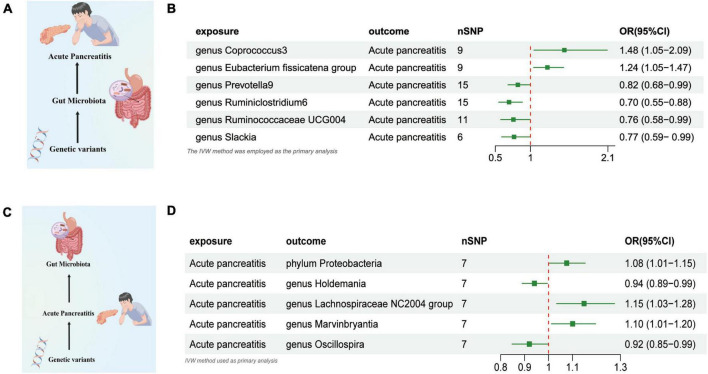
**(A)** Causal effect of gut microbiota with Acute Pancreatitis Schematic representation of the MR analysis results. **(B)** Forest plot of the MR analysis results. **(C)** Causal effect of Acute Pancreatitis with gut microbiota Schematic representation of the Reverse MR analysis results. **(D)** Forest plot of the MR analysis results. OR, odds ratio; CI, confidence interval; IVW, inverse variance weighted method. Significant threshold was set at *p*-value < 0.05 for the Inverse Variance Weighted method (IVW).

Based on the IVW test estimates, six genera are genetically predicted to be abundant, namely, *Coprococcus3, Eubacterium fissicatena group, Prevotella9, Ruminiclostridium6*, and *Ruminococcaceae UCG004*,Researchers found that *slackia* increased or reduced AP risk a higher genetically predicted abundance of *Genus Coprococcus3* was linked to a higher risk of AP (OR: 1.480, 95% CI: 1.049–2.089, *P* = 0.048). The genetically predicted abundance of *Genus Eubacterium Fissicatena Group* was also associated with a Increased risk of AP (OR: 1.240, 95% CI: 1.045–1.471, *P* = 0.013). A higher genetically predicted abundance of *Genus Prevotella9* was related to a reduced risk of AP (OR: 0.820, 95% CI: 0.680–0.989, *P* = 0.038). The genetically predicted abundance of *Genus Ruminiclostridium6* was also associated with a reduced risk of AP (OR: 0.696, 95% CI: 0.548–0.883, *P* = 0.002). The genetically predicted abundance of *Genus Ruminococcaceae UCG004* was negatively related to the risk of AP (OR: 0.757, 95% CI: 0.576–0.994, *P* = 0.045). The higher genetically predicted abundance of *Genus Slackia* was also linked to a reduced risk of AP (OR: 0.766, 95% CI: 0.590–0.996, *P* = 0.046) (Figures 2A, B and Supplementary Table 1).

#### AP and gut microbiome

We used AP as the exposure and gut microbiota as the outcome to assess any reverse causation effects, our analysis indicated that a genetically predisposed likelihood of AP was potentially correlated with an enhanced prevalence of the *phylum Proteobacteria*, (OR = 1.075 [95% CI: 1.003–1.152], *P* = 0.038). The study further demonstrated a potential correlation with an increased prevalence of the *Lachnospiraceae NC2004 Group* (OR = 1.148 [95% CI: 1.033–1.275], *P* = 0.010) and the *Genus Marvinbryantia* (OR = 1.100 [95% CI: 1.012–1.196], *P* = 0.024). Conversely, we observed an inverse correlation with the abundance of the *Genus Holdemania* (OR = 0.914 [95% CI: 0.839–0.997], *P* = 0.043), and the *Genus Oscillospira* (OR = 0.919 [95% CI: 0.847–0.998], *P* = 0.045) (Figures 2C, D and Supplementary Table 2).

#### Gut microbiome and CP

**FIGURE 3:**
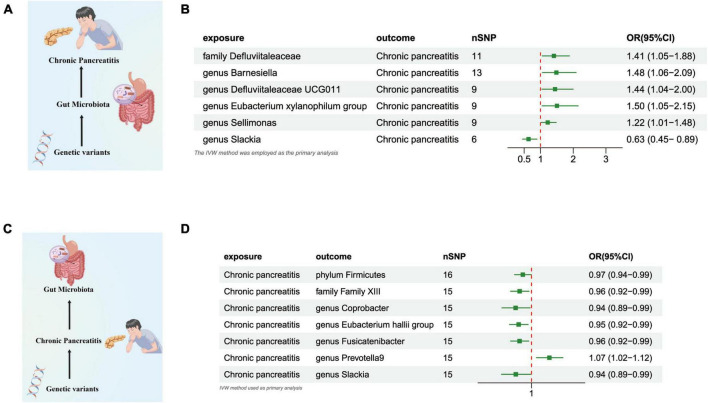
**(A)** Causal effect of gut microbiota with Chronic Pancreatitis Schematic representation of the MR analysis results. **(B)** Forest plot of the MR analysis results. **(C)** Causal effect of Chronic Pancreatitis with gut microbiota Schematic representation of the Reverse MR analysis results. **(D)** Forest plot of the MR analysis results. OR, odds ratio; CI, confidence interval; IVW, inverse variance weighted method.

Our research revealed a potential correlation between the genetically anticipated presence of the *Genus Slackia*, (OR = 0.633 [95% CI: 0.449–0.892], *P* = 0.009), and a reduced susceptibility to CP. Conversely, a genetic predisposition to the *Family Defluviitaleaceae* (OR = 1.408 [95% CI: 1.053–1.883], *P* = 0.020), the *Genus Barnesiella* (OR = 1.484 [95% CI: 1.055–2.088], *P* = 0.023), the *Genus Defluviitaleaceae UCG011* (OR = 1.443 [95% CI: 1.042–1.998], *P* = 0.027), the *Eubacterium xylanophilum Group* (OR = 1.502 [95% CI: 1.049–2.150], *P* = 0.026), and the *Genus Sellimonas* (OR = 1.219 [95% CI: 1.006–1.478], *P* = 0.042) was potentially associated with an elevated susceptibility to CP (Figures 3A, B and Supplementary Table 3).

#### CP and gut microbiome

In the reverse direction MR analysis, we explored the potential causative relationship of CP influencing the gut microbiota composition at varying taxonomic levels, including the *Phylum*, *Family*, and *Genus*. In our analysis, a genetic predisposition toward CP demonstrated a potential correlation with an increased prevalence of the *Genus Prevotella9*, (OR = 1.067 [95% CI: 1.018–1.117], *P* = 0.006). Conversely, our results indicated an inverse association with the abundance of the *Phylum Firmicutes* (OR = 0.967 [95% CI: 0.936–0.999], *P* = 0.047), the *Family Family XIII* (OR = 0.955 [95% CI: 0.921–0.990], *P* = 0.014), the *Genus Coprobacter* (OR = 0.940 [95% CI: 0.889–0.995], *P* = 0.032), the *Eubacterium Hallii Group* (OR = 0.952 [95% CI: 0.918–0.987], *P* = 0.008), the *Genus Fusicatenibacter* (OR = 0.955 [95% CI: 0.922–0.989], *P* = 0.011), and the *Genus Slackia* (OR = 0.941 [95% CI: 0.888–0.998], *P* = 0.043) (Figures 3C, D and Supplementary Table 4).

#### Gut microbiome and AAP

**FIGURE 4:**
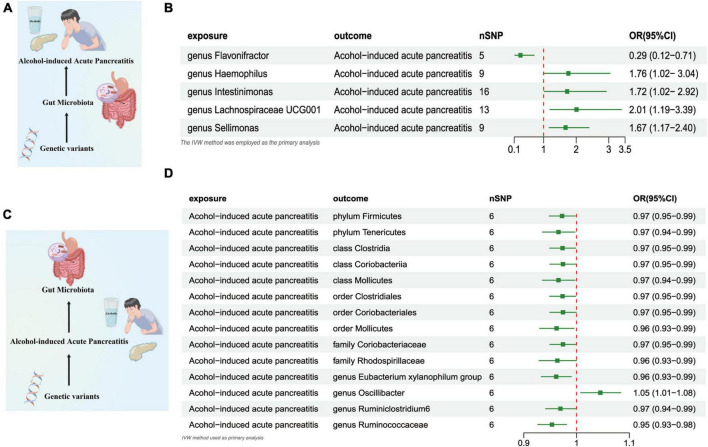
**(A)** Causal effect of gut microbiota with Alcohol-induced Acute Pancreatitis Schematic representation of the MR analysis results. **(B)** Forest plot of the MR analysis results. **(C)** Causal effect of Alcohol-induced Acute Pancreatitis with gut microbiota Schematic representation of the Reverse MR analysis results. **(D)** Forest plot of the MR analysis results. OR, odds ratio; CI, confidence interval; IVW, inverse variance weighted method; NSNPs, number of single nucleotide polymorphisms.

The outcomes of the IVW test revealed a significant negative association between the genetically predicted relative abundance of the specific genus, *flavonifractor* (OR = 0.290, [95% CI: 0.12–0.71], *P* = 0.006), and the susceptibility to AAP. On the other hand, the genetically anticipated relative abundance of four distinct *Genera*, namely, *Haemophilus* (OR = 1.760, [95% CI: 1.020–3.037], *P* = 0.041), *lntestinimonas* (OR = 1.724, [95% CI: 1.017–2.924], *P* = 0.043), *Lachnospiraceae UCG001* (OR = 2.011, [95% CI: 1.193–3.389], *P* = 0.008), and *Sellimonas* (OR = 1.673, [95% CI: 1.169–2.395], *P* = 0.004), demonstrated a positive correlation with the risk of AAP (Figures 4A, B and Supplementary Table 5).

#### AAP and gut microbiome

In the reverse direction MR analysis, we explored the potential causative relationship of AAP influencing the gut microbiota composition at varying taxonomic levels, including the *Phylum*, *Class, Family*, and *Genus*. In our analysis, a genetic predisposition toward AAP demonstrated a potential correlation with an increased of the *Genus Oscillibacter*, (OR = 1.045 [95% CI: 1.007–1.083], *P* = 0.017). Conversely, our results indicated an inverse association with the abundance of the *Phylum Firmicutes* (OR = 0.972 [95% CI: 0.948–0.997], *P* = 0.031), the *Phylum Tenericutes* (OR = 0.965 [95% CI: 0.935–0.997], *P* = 0.032), the *Class Clostridia* (OR = 0.973 [95% CI: 0.949–0.998], *P* = 0.036), *Class Coriobacteriia* (OR = 0.974 [95% CI: 0.949–0.999], *P* = 0.046), the *Class Mollicutes* (OR = 0.965 [95% CI: 0.935–0.997], *P* = 0.032), and the *Family Coriobacteriaceae* (OR = 0.974 [95% CI: 0.949–0.999], *P* = 0.046), the *Family Rhodospirillaceae* (OR = 0.963 [95% CI: 0.928–0.999], *P* = 0.048), *Order Clostridiales* (OR = 0.973 [95% CI: 0.949–0.998], *P* = 0.036),the *Order Coriobacteriales* (OR = 0.974 [95%CI: 0.949–0.999], *P* = 0.046),*Order Mollicutes* (OR = 0.962 [95%CI: 0.930–0.995], *P* = 0.024), the *Class Clostridia* (OR = 0.973 [95% CI: 0.949–0.998], *P* = 0.036), *Genus Eubacterium Xylanophilum Group* (OR = 0.961 [95% CI: 0.933–0.990], *P* = 0.009),the *Genus Ruminiclostridium6* (OR = 0.969 [95% CI: 0.941–0.998], *P* = 0.039),and *Genus Ruminococcaceae UCG014* (OR = 0.953 [95% CI: 0.926–0.980], *P* = 0.001) (Figures 4C, D and Supplementary Table 6).

#### Gut microbiome and ACP

**FIGURE 5:**
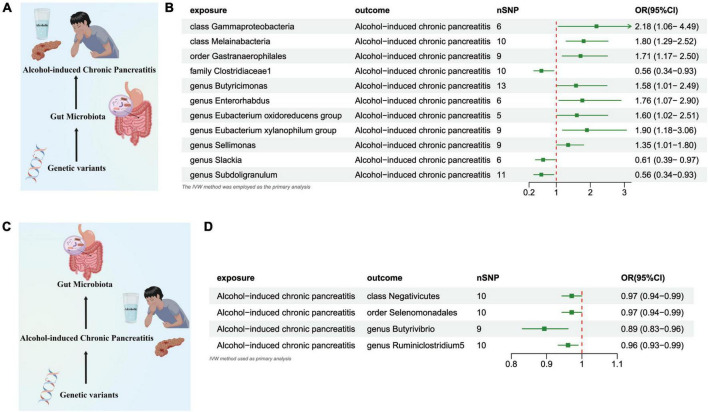
**(A)** Causal effect of gut microbiota with Alcohol-induced Chronic Pancreatitis Schematic representation of the MR analysis results. **(B)** Forest plot of the MR analysis results. **(C)** Causal effect of Alcohol-induced Chronic Pancreatitis with gut microbiota Schematic representation of the Reverse MR analysis results. **(D)** Forest plot of the MR analysis results. OR, odds ratio; CI, confidence interval; IVW, inverse variance weighted method.

The results from the IVW test provided estimations suggesting that the genetically predicted relative abundance of 2 taxa at the class level, 1 taxon at the order level, 1 taxon at the family level, and 7 taxa at the genus level exhibited associations with either an elevated or diminished risk of ACP. In our analysis, a genetic predisposition toward ACP demonstrated a potential correlation with an increased of the *Genus Oscillibacter* (OR = 2.179 [95% CI: 1.057–4.449], *P* = 0.034), the *Class Melainabacteria* (OR = 1.801 [95% CI: 1.288–2.519], *P* = 0.0005), the *Order Gastranaerophilales* (OR = 1.712 [95% CI: 1.171–2.503], *P* = 0.005), the *Genus Butyricimonas* (OR = 1.579 [95% CI: 1.001–2.488], *P* = 0.049),the *Genus Oscillibacter* (OR = 2.179 [95% CI: 1.057–1.083], *P* = 4.449), the *Genus Enterorhabdus* (OR = 1.764 [95% CI: 1.073–2.902], *P* = 0.025), the *Genus Eubacterium Oxidoreducens Group* (OR = 1.602 [95% CI: 1.023–2.510], *P* = 0.039), *Genus Eubacterium Xylanophilum Group* (OR = 1.899 [95% CI: 1.179–3.060], *P* = 0.008, the *Genus Sellimonas* (OR = 1.347 [95% CI: 1.006–1.803], *P* = 0.048. Conversely, our results indicated an inverse association with the abundance of the *Family Clostridiaceae1* (OR = 0.563 [95% CI: 0.342–0.928], *P* = 0.024), the *Genus Slackia* (OR = 0.614 [95% CI: 0.388–0.971], *P* = 0.037), the *Genus Subdoligranulum* (OR = 0.561 [95% CI: 0.339–0.928], *P* = 0.024) (Figures 5A, B and Supplementary Table 7).

#### ACP and gut microbiome

In the reverse direction MR analysis, we explored the potential causative relationship of ACP influencing the gut microbiota composition at varying taxonomic levels, including the *Class, Order*, and *Genus.* Our findings suggested that a genetic inclination toward ACP may be associated with a consequential reduction in the presence of the *Class Negativicutes* (OR = 0.971 [95% CI: 0.943–0.999], *P* = 0.046), the *Order Selenomonadales* (OR = 0.971 [95% CI: 0.943–0.999], *P* = 0.046), and the *Genera Butyrivibrio* (OR = 0.893 [95%CI: 0.831–0.960], *P* = 0.002) and *Ruminiclostridium5* (OR = 0.960 [95% CI: 0.932–0.989], *P* = 0.007) (Figures 5C, D and Supplementary Table 8).

#### Sensitivity analyses, Bonferroni-corrected test

The outcomes derived from the Bonferroni-adjusted analysis indicate a robust causative association between elevated levels of the *Class Melainabacteria* (OR = 1.801, 95% CI: 1.288–2.519, *P* = 0.008) and ACP. Following the Bonferroni test, our investigation revealed no discernible genetic predisposition to gut microbiota that exhibited a causal association with other forms of pancreatitis. Utilizing Cochran’s Q and MR-PRESSO assessments, we observed no heterogeneity (*P* > 0.05) nor any detectable outliers as listed in Supplementary Table 8. All *P*-values resulting from the MR-Egger interpretation exceeded 0.05, thereby indicating a lack of horizontal pleiotropy (Supplementary Table 8). Firstly, the application of the “Leave-one-out” analytical approach substantiated the stability and credibility of our principal outcomes (Supplementary Figures 1–5), furthermore, to evaluate the reciprocal causative impact of pancreatitis on the gut microbiome, we employed four supplementary methodologies, namely MR-Egger, weighted median, simple mode, and weighted mode (Supplementary Figures 6–10), Forest plots for causal effects of gut microbiota on four types of pancreatitis risk with individual SNPs. In reverse MR analysis, The Forest plots for association between four types of pancreatitis and gut microbiota (Supplementary Figures 11–15).

## Discussion

To the extent of our knowledges, our research constitutes the first wide-ranging, in-depth MR analysis aimed at examining the cause-and-effect relationship between intestinal microbiota and pancreatitis at a genetic prediction scale. Prior research probing the relationship between gut microbiota and pancreatitis has predominantly utilized animal models as the primary mode of investigation (Zhang et al., 2019; Zhu et al., 2019; Glaubitz et al., 2023; Hu et al., 2023; Jiao et al., 2023; Liu L. et al., 2023; Wang Z. et al., 2023). These findings might bear significance for public health initiatives that are targeted toward mitigating the risk associated with pancreatitis.

An escalating volume of scholarly research has identified a potential correlation between the specific gut microbiota examined in our study and the incidence of pancreatitis. We successfully found that patients with acute pancreatitis were associated with elevated phylum Proteobacteria. Lachnospiraceae NC2004 group genus *Marvinbryantia* intestinal flora and decreased genus Holdemania genus *Oscillospira* bacteria. In the reverse direction MR analysis study, Patients with acute pancreatitis contain more Proteobacteria bacteria and Marvinbryantia, which was consistent with Zhang’s research and Wang’s (Zhang et al., 2018; Wang et al., 2020). At the phylum classification, the gut microbiome of CP exhibited increased abundance of Proteobacteria. These findings are consistent with recent scholarly investigations, suggesting that individuals afflicted with CP display perturbations in gut microbiota, characterized by a decline in both diversity and richness, accompanied by changes in taxonomic distribution (Zhou et al., 2020).

The influence of the gut microbiota on pancreatitis has been scrutinized across various research investigations. These studies (Liang et al., 2022; Wang et al., 2022a,b; Zhang et al., 2022; Zou et al., 2022; Hu et al., 2023; Liu L.-W. et al., 2023; Liu L. et al., 2023; Wang J. et al., 2023; Xu et al., 2023) have revealed that the progression of pancreatitis could potentially be modulated by the gut microbiome, either via its impact on microbial translocation or through mediating the host’s immune response (Brestoff and Artis, 2013; Sun et al., 2015; Ahuja et al., 2017; Xu et al., 2022). Contemporary research has demonstrated variability in the gut microbial composition when comparing individuals with pancreatitis to healthy control subjects. Zhang et al. (2018) employed high-throughput 16S rRNA gene amplicon sequencing to investigate the composition of the gut microbiome in a cohort comprising 45 patients with AP in comparison to 44 healthy individuals. The authors conducted a comparative analysis between the two cohorts and observed significant differences in the composition of gut microbiota in patients with AP. Notably, AP patients exhibited a substantial reduction in phyla diversity compared to the control group. Lei et al. (2021) discovered that Parabacteroides could attenuate AP in heparanase-transgenic mice through minimizing neutrophil infiltration.

The principal advantage of this study is the implementation of a MR design, which mitigates potential confounders and reverse causality, thereby enhancing the capacity to infer causality in the observed associations. Hence, the outcomes garnered are more veritable, offering a dependable interpretation effect based on causality. Furthermore, the application of reverse MR and sensitivity analysis unveiled no signs of pleiotropy or heterogeneity, thereby affirming the statistical robustness of our results. These research insights emphasize the role of gut microbiota in driving pancreatitis and acting as a supplementary component that could assist in patient risk stratification. Additionally, they present opportunities for identifying potential therapeutic targets based on gut microbiota. These outcomes could hold significance for the formulation of individualized therapeutic strategies aimed at pancreatitis prevention and survival enhancement.

However, our study was subject to various limitations. First, it is essential to acknowledge that the majority of participants in the GWAS summary data utilized for this investigation were of European descent; nonetheless, a smaller subset of non-European individuals was also included in the GWAS dataset, this demographic composition could potentially introduce biases in the estimates and impact the generalizability or universality of the findings. Second, the use of MR to select the gut microbiome as an exposure also has limitations. Notably, the abundance of gut microbiota may be affected by factors such as diet, gender, medication, and sampling time. These factors could potentially reduce the proportion of variance explained by genetics. Thirdly, the utilization of varying cohorts implies that non-linear associations cannot be examined, particularly considering the possible variations in the gut microbiome across cohorts, making it impossible to conduct a subgroup analysis. Nevertheless, the consistency of causal effects should be generally maintained among different cohorts.

## Conclusion

Our first systematic Mendelian randomization analysis provides evidence that multiple gut microbiota taxa may be causally associated with four types of pancreatitis disease. This discovery may contribute significant biomarkers conducive to the preliminary, non-invasive identification of Pancreatitis. Additionally, it could present viable targets for potential therapeutic interventions in the disease’s treatment.

## Data availability statement

The original contributions presented in this study are included in this article/Supplementary material, further inquiries can be directed to the corresponding authors.

## Ethics statement

All the data utilized in this investigation are publicly accessible and fall within the public domain. All participants granted informed consent, and the study protocols received approval from their respective local Ethical Committees (Kurilshikov et al., 2021). The present study received approval from the Institutional Review Board of Ruijin Hospital, Shanghai Jiao Tong University.

## Author contributions

KW: Conceptualization, Data curation, Investigation, Software, Visualization, Writing – original draft, Writing – review and editing, Formal analysis, Methodology, Project administration. XQ: Conceptualization, Investigation, Software, Writing – original draft, Writing – review and editing. TR: Conceptualization, Investigation, Writing – original draft, Writing – review and editing, Methodology, Supervision, Visualization. YP: Conceptualization, Data curation, Methodology, Supervision, Validation, Writing – review and editing. YH: Conceptualization, Formal analysis, Supervision, Visualization, Writing – review and editing. JW: Investigation, Project administration, Validation, Visualization, Writing – review and editing. XZ: Formal analysis, Investigation, Methodology, Supervision, Writing – review and editing. XS: Formal analysis, Investigation, Methodology, Project administration, Writing – review and editing. CL: Investigation, Project administration, Supervision, Validation, Writing – review and editing. XL: Data curation, Formal analysis, Software, Validation, Writing – review and editing. YC: Data curation, Formal analysis, Investigation, Methodology, Writing – review and editing. YB: Software, Supervision, Validation, Writing – review and editing. YZ: Investigation, Methodology, Software, Writing – review and editing. CZ: Conceptualization, Data curation, Funding acquisition, Investigation, Methodology, Project administration, Resources, Supervision, Validation, Writing – review and editing. DZ: Conceptualization, Funding acquisition, Investigation, Methodology, Project administration, Resources, Software, Supervision, Validation, Writing – review and editing.

## Footnotes

1
https://finngen.gitbook.io/documentation

2
https://risteys.finngen.fi/phenocode/ALCOPANCACU

3
https://risteys.finngen.fi/endpoints/K11_ACUTPANC

4
https://risteys.finngen.fi/phenocode/ALCOPANCCHRON

5
https://risteys.finngen.fi/phenocode/K11_CHRONPANC
