# Supplementary material for: Causal link between gut microbiota and four types of pancreatitis: a genetic association and bidirectional Mendelian randomization study
Source: Front Microbiol. 2023 Nov 23;14:1290202. doi: 10.3389/fmicb.2023.1290202 (PMC10702359; doi:10.3389/fmicb.2023.1290202)
Supplement: Supplementary file 2 [file Table_2.DOCX]

| **Supplementary Table 1:MR results and sensitivity analysis of gut microbiota on AP** | | | | | | | | | | | | | | | | | | | | | | | | | | | | | | | | | | | | | | | | | | | | | | | | | | | | | | | | | | | | | | | | | | | | | | | | | | | | | | | | | | | | | | | | | | | | | | | | | | | | | | | | | | |
| --- | --- | --- | --- | --- | --- | --- | --- | --- | --- | --- | --- | --- | --- | --- | --- | --- | --- | --- | --- | --- | --- | --- | --- | --- | --- | --- | --- | --- | --- | --- | --- | --- | --- | --- | --- | --- | --- | --- | --- | --- | --- | --- | --- | --- | --- | --- | --- | --- | --- | --- | --- | --- | --- | --- | --- | --- | --- | --- | --- | --- | --- | --- | --- | --- | --- | --- | --- | --- | --- | --- | --- | --- | --- | --- | --- | --- | --- | --- | --- | --- | --- | --- | --- | --- | --- | --- | --- | --- | --- | --- | --- | --- | --- | --- | --- | --- | --- | --- | --- | --- | --- | --- | --- | --- | --- | --- |
| **Taxa** | | | | | | **Gut microbiota (exposure)** | | | | | | | | **Trait (outcome)** | | | | | | | **Nsnp** | | | | | | **Methods** | | | | | | | | **Beta** | | | | | | | | **SE** | | | | | | | | **OR (95%CI)** | | | | | | | | ***P* value** | | | | | | | | **MR-PRESSO** | | | | | | | | **Heterogeneity** | | | | | | | | | | | | | | **Horizontal pleiotrop** | | | | | | | | | | | | | | | | | |
|  |  |  |  |  |  |  |  |  |  |  |  |  |  |  |  |  |  |  |  |  |  |  |  |  |  |  |  |  |  |  |  |  |  |  |  |  |  |  |  |  |  |  |  |  |  |  |  |  |  |  |  |  |  |  |  |  |  |  |  |  |  |  |  |  |  |  |  |  |  |  |  |  |  |  | **Cochran’s Q** | | | | | | | ***P* value** | | | | | | | **Egger intercept** | | | | | | | | **SE** | | | | | | | | ***P* value** | |
| genus | | | | | | Coprococcus3 | | | | | | | | Acute pancreatitis | | | | | | | 9 | | | | | | MR-Egger | | | | | | | | 0.24 | | | | | | | | 0.98 | | | | | | | | 1.27 (0.19-8.76) | | | | | | | | 0.812 | | | | | | | | 0.86 | | | | | | | | 4.964 | | | | | | | 0.7613 | | | | | | | 0.0090 | | | | | | | | 0.058 | | | | | | | | 0.880 | |
|  |  |  |  |  |  |  |  |  |  |  |  |  |  |  |  |  |  |  |  |  |  |  |  |  |  |  | Weighted median | | | | | | | | 0.31 | | | | | | | | 0.23 | | | | | | | | 1.37 (0.87-2.18) | | | | | | | | 0.176 | | | | | | | |  |  |  |  |  |  |  |  |  |  |  |  |  |  |  |  |  |  |  |  |  |  |  |  |  |  |  |  |  |  |  |  |  |  |  |  |  |  |  |  |
|  |  |  |  |  |  |  |  |  |  |  |  |  |  |  |  |  |  |  |  |  |  |  |  |  |  |  | Inverse variance weighted | | | | | | | | 0.39 | | | | | | | | 0.17 | | | | | | | | 1.48 (1.05-2.09) | | | | | | | | 0.025 | | | | | | | |  |  |  |  |  |  |  |  |  |  |  |  |  |  |  |  |  |  |  |  |  |  |  |  |  |  |  |  |  |  |  |  |  |  |  |  |  |  |  |  |
|  |  |  |  |  |  |  |  |  |  |  |  |  |  |  |  |  |  |  |  |  |  |  |  |  |  |  | Simple mode | | | | | | | | 0.21 | | | | | | | | 0.37 | | | | | | | | 1.23 (0.59-2.57) | | | | | | | | 0.588 | | | | | | | |  |  |  |  |  |  |  |  |  |  |  |  |  |  |  |  |  |  |  |  |  |  |  |  |  |  |  |  |  |  |  |  |  |  |  |  |  |  |  |  |
|  |  |  |  |  |  |  |  |  |  |  |  |  |  |  |  |  |  |  |  |  |  |  |  |  |  |  | Weighted mode | | | | | | | | 0.15 | | | | | | | | 0.32 | | | | | | | | 1.16 (0.61-2.21) | | | | | | | | 0.654 | | | | | | | |  |  |  |  |  |  |  |  |  |  |  |  |  |  |  |  |  |  |  |  |  |  |  |  |  |  |  |  |  |  |  |  |  |  |  |  |  |  |  |  |
| genus | | | | | | Eubacterium fissicatena group | | | | | | | | Acute pancreatitis | | | | | | | 9 | | | | | | MR-Egger | | | | | | | | 0.480 | | | | | | | | 0.45 | | | | | | | | 1.62 (0.67-3.92) | | | | | | | | 0.3235 | | | | | | | | 0.98 | | | | | | | | 2.366 | | | | | | | 0.9676 | | | | | | | -0.0348 | | | | | | | | 0.0583 | | | | | | | | 0.569 | |
|  |  |  |  |  |  |  |  |  |  |  |  |  |  |  |  |  |  |  |  |  |  |  |  |  |  |  | Weighted median | | | | | | | | 0.189 | | | | | | | | 0.107 | | | | | | | | 1.21 (0.98-1.49) | | | | | | | | 0.0766 | | | | | | | |  |  |  |  |  |  |  |  |  |  |  |  |  |  |  |  |  |  |  |  |  |  |  |  |  |  |  |  |  |  |  |  |  |  |  |  |  |  |  |  |
|  |  |  |  |  |  |  |  |  |  |  |  |  |  |  |  |  |  |  |  |  |  |  |  |  |  |  | Inverse variance weighted | | | | | | | | 0.21 | | | | | | | | 0.087 | | | | | | | | 1.24 (1.05-1.47) | | | | | | | | 0.013 | | | | | | | |  |  |  |  |  |  |  |  |  |  |  |  |  |  |  |  |  |  |  |  |  |  |  |  |  |  |  |  |  |  |  |  |  |  |  |  |  |  |  |  |
|  |  |  |  |  |  |  |  |  |  |  |  |  |  |  |  |  |  |  |  |  |  |  |  |  |  |  | Simple mode | | | | | | | | 0.18 | | | | | | | | 0.168 | | | | | | | | 1.21 (0.87-1.68) | | | | | | | | 0.297 | | | | | | | |  |  |  |  |  |  |  |  |  |  |  |  |  |  |  |  |  |  |  |  |  |  |  |  |  |  |  |  |  |  |  |  |  |  |  |  |  |  |  |  |
|  |  |  |  |  |  |  |  |  |  |  |  |  |  |  |  |  |  |  |  |  |  |  |  |  |  |  | Weighted mode | | | | | | | | 0.18 | | | | | | | | 0.168 | | | | | | | | 1.20 (0.86-1.68) | | | | | | | | 0.305 | | | | | | | |  |  |  |  |  |  |  |  |  |  |  |  |  |  |  |  |  |  |  |  |  |  |  |  |  |  |  |  |  |  |  |  |  |  |  |  |  |  |  |  |
| genus | | | | | | Prevotella9 | | | | | | | | Acute pancreatitis | | | | | | | 15 | | | | | | MR-Egger | | | | | | | | -0.08 | | | | | | | | 0.278 | | | | | | | | 0.92 (0.53-1.59) | | | | | | | | 0.771 | | | | | | | | 0.77 | | | | | | | | 10.124 | | | | | | | 0.7530 | | | | | | | -0.012 | | | | | | | | 0.0273 | | | | | | | | 0.6673 | |
|  |  |  |  |  |  |  |  |  |  |  |  |  |  |  |  |  |  |  |  |  |  |  |  |  |  |  | Weighted median | | | | | | | | -0.23 | | | | | | | | 0.129 | | | | | | | | 0.79 (0.61-1.02) | | | | | | | | 0.067 | | | | | | | |  |  |  |  |  |  |  |  |  |  |  |  |  |  |  |  |  |  |  |  |  |  |  |  |  |  |  |  |  |  |  |  |  |  |  |  |  |  |  |  |
|  |  |  |  |  |  |  |  |  |  |  |  |  |  |  |  |  |  |  |  |  |  |  |  |  |  |  | Inverse variance weighted | | | | | | | | -0.19 | | | | | | | | 0.09 | | | | | | | | 0.82 (0.68-0.99) | | | | | | | | 0.038 | | | | | | | |  |  |  |  |  |  |  |  |  |  |  |  |  |  |  |  |  |  |  |  |  |  |  |  |  |  |  |  |  |  |  |  |  |  |  |  |  |  |  |  |
|  |  |  |  |  |  |  |  |  |  |  |  |  |  |  |  |  |  |  |  |  |  |  |  |  |  |  | Simple mode | | | | | | | | -0.27 | | | | | | | | 0.21 | | | | | | | | 0.76 (0.50-1.16) | | | | | | | | 0.223 | | | | | | | |  |  |  |  |  |  |  |  |  |  |  |  |  |  |  |  |  |  |  |  |  |  |  |  |  |  |  |  |  |  |  |  |  |  |  |  |  |  |  |  |
|  |  |  |  |  |  |  |  |  |  |  |  |  |  |  |  |  |  |  |  |  |  |  |  |  |  |  | Weighted mode | | | | | | | | -0.26 | | | | | | | | 0.188 | | | | | | | | 0.77 (0.53-1.11) | | | | | | | | 0.181 | | | | | | | |  |  |  |  |  |  |  |  |  |  |  |  |  |  |  |  |  |  |  |  |  |  |  |  |  |  |  |  |  |  |  |  |  |  |  |  |  |  |  |  |
| genus | | | | | | Ruminiclostridium6 | | | | | | | | Acute pancreatitis | | | | | | | 15 | | | | | | MR-Egger | | | | | | | | -0.05 | | | | | | | | 0.30 | | | | | | | | 0.94 (0.52-1.71) | | | | | | | | 0.854 | | | | | | | | 0.91 | | | | | | | | 6.944 | | | | | | | 0.9368 | | | | | | | -0.028 | | | | | | | | 0.0260 | | | | | | | | 0.2898 | |
|  |  |  |  |  |  |  |  |  |  |  |  |  |  |  |  |  |  |  |  |  |  |  |  |  |  |  | Weighted median | | | | | | | | -0.36 | | | | | | | | 0.16 | | | | | | | | 0.70 (0.50-0.96) | | | | | | | | 0.029 | | | | | | | |  |  |  |  |  |  |  |  |  |  |  |  |  |  |  |  |  |  |  |  |  |  |  |  |  |  |  |  |  |  |  |  |  |  |  |  |  |  |  |  |
|  |  |  |  |  |  |  |  |  |  |  |  |  |  |  |  |  |  |  |  |  |  |  |  |  |  |  | Inverse variance weighted | | | | | | | | -0.36 | | | | | | | | 0.12 | | | | | | | | 0.70 (0.55-0.88) | | | | | | | | 0.002 | | | | | | | |  |  |  |  |  |  |  |  |  |  |  |  |  |  |  |  |  |  |  |  |  |  |  |  |  |  |  |  |  |  |  |  |  |  |  |  |  |  |  |  |
|  |  |  |  |  |  |  |  |  |  |  |  |  |  |  |  |  |  |  |  |  |  |  |  |  |  |  | Simple mode | | | | | | | | -0.34 | | | | | | | | 0.27 | | | | | | | | 0.71 (0.41-1.21) | | | | | | | | 0.226 | | | | | | | |  |  |  |  |  |  |  |  |  |  |  |  |  |  |  |  |  |  |  |  |  |  |  |  |  |  |  |  |  |  |  |  |  |  |  |  |  |  |  |  |
|  |  |  |  |  |  |  |  |  |  |  |  |  |  |  |  |  |  |  |  |  |  |  |  |  |  |  | Weighted mode | | | | | | | | -0.26 | | | | | | | | 0.26 | | | | | | | | 0.76 (0.45-1.29) | | | | | | | | 0.330 | | | | | | | |  |  |  |  |  |  |  |  |  |  |  |  |  |  |  |  |  |  |  |  |  |  |  |  |  |  |  |  |  |  |  |  |  |  |  |  |  |  |  |  |
| genus | | | | | | Ruminococcaceae UCG004 | | | | | | | | Acute pancreatitis | | | | | | | 11 | | | | | | MR-Egger | | | | | | | | -0.92 | | | | | | | | 0.78 | | | | | | | | 0.40 (0.09-1.85) | | | | | | | | 0.269 | | | | | | | | 0.22 | | | | | | | | 14.101 | | | | | | | 0.1684 | | | | | | | 0.0549 | | | | | | | | 0.0657 | | | | | | | | 0.425 | |
|  |  |  |  |  |  |  |  |  |  |  |  |  |  |  |  |  |  |  |  |  |  |  |  |  |  |  | Weighted median | | | | | | | | -0.12 | | | | | | | | 0.16 | | | | | | | | 0.88 (0.63-1.22) | | | | | | | | 0.436 | | | | | | | |  |  |  |  |  |  |  |  |  |  |  |  |  |  |  |  |  |  |  |  |  |  |  |  |  |  |  |  |  |  |  |  |  |  |  |  |  |  |  |  |
|  |  |  |  |  |  |  |  |  |  |  |  |  |  |  |  |  |  |  |  |  |  |  |  |  |  |  | Inverse variance weighted | | | | | | | | -0.27 | | | | | | | | 0.13 | | | | | | | | 0.76 (0.58-0.99) | | | | | | | | 0.045 | | | | | | | |  |  |  |  |  |  |  |  |  |  |  |  |  |  |  |  |  |  |  |  |  |  |  |  |  |  |  |  |  |  |  |  |  |  |  |  |  |  |  |  |
|  |  |  |  |  |  |  |  |  |  |  |  |  |  |  |  |  |  |  |  |  |  |  |  |  |  |  | Simple mode | | | | | | | | -0.12 | | | | | | | | 0.27 | | | | | | | | 0.88 (0.52-1.50) | | | | | | | | 0.652 | | | | | | | |  |  |  |  |  |  |  |  |  |  |  |  |  |  |  |  |  |  |  |  |  |  |  |  |  |  |  |  |  |  |  |  |  |  |  |  |  |  |  |  |
|  |  |  |  |  |  |  |  |  |  |  |  |  |  |  |  |  |  |  |  |  |  |  |  |  |  |  | Weighted mode | | | | | | | | -0.14 | | | | | | | | 0.24 | | | | | | | | 0.86 (0.53-1.40) | | | | | | | | 0.565 | | | | | | | |  |  |  |  |  |  |  |  |  |  |  |  |  |  |  |  |  |  |  |  |  |  |  |  |  |  |  |  |  |  |  |  |  |  |  |  |  |  |  |  |
| genus | | | | | | Slackia | | | | | | | | Acute pancreatitis | | | | | | | 6 | | | | | | MR-Egger | | | | | | | | 0.91 | | | | | | | | 0.86 | | | | | | | | 2.49 (0.45-13.62) | | | | | | | | 0.352 | | | | | | | | 0.57 | | | | | | | | 3.792 | | | | | | | 0.579 | | | | | | | -0.1184 | | | | | | | | 0.086 | | | | | | | | 0.2417 | |
|  |  |  |  |  |  |  |  |  |  |  |  |  |  |  |  |  |  |  |  |  |  |  |  |  |  |  | Weighted median | | | | | | | | -0.29 | | | | | | | | 0.17 | | | | | | | | 0.74  (0.53- 1.04) | | | | | | | | 0.080 | | | | | | | |  |  |  |  |  |  |  |  |  |  |  |  |  |  |  |  |  |  |  |  |  |  |  |  |  |  |  |  |  |  |  |  |  |  |  |  |  |  |  |  |
|  |  |  |  |  |  |  |  |  |  |  |  |  |  |  |  |  |  |  |  |  |  |  |  |  |  |  | Inverse variance weighted | | | | | | | | -0.26 | | | | | | | | 0.13 | | | | | | | | 0.77  (0.59- 0.99) | | | | | | | | 0.046 | | | | | | | |  |  |  |  |  |  |  |  |  |  |  |  |  |  |  |  |  |  |  |  |  |  |  |  |  |  |  |  |  |  |  |  |  |  |  |  |  |  |  |  |
|  |  |  |  |  |  |  |  |  |  |  |  |  |  |  |  |  |  |  |  |  |  |  |  |  |  |  | Simple mode | | | | | | | | -0.46 | | | | | | | | 0.27 | | | | | | | | 0.63  (0.37- 1.07) | | | | | | | | 0.149 | | | | | | | |  |  |  |  |  |  |  |  |  |  |  |  |  |  |  |  |  |  |  |  |  |  |  |  |  |  |  |  |  |  |  |  |  |  |  |  |  |  |  |  |
|  |  |  |  |  |  |  |  |  |  |  |  |  |  |  |  |  |  |  |  |  |  |  |  |  |  |  | Weighted mode | | | | | | | | -0.45 | | | | | | | | 0.28 | | | | | | | | 0.63  (0.36- 1.10) | | | | | | | | 0.168 | | | | | | | |  |  |  |  |  |  |  |  |  |  |  |  |  |  |  |  |  |  |  |  |  |  |  |  |  |  |  |  |  |  |  |  |  |  |  |  |  |  |  |  |
| **Supplementary Table 2:In reverse MR results and sensitivity analysis of AP on gut microbiota** | | | | | | | | | | | | | | | | | | | | | | | | | | | | | | | | | | | | | | | | | | | | | | | | | | | | | | | | | | | | | | | | | | | | | | | | | | | | | | | | | | | | | | | | | | | | | | | | | | | | | | | | | | |
| **Taxa** | **exposure** | | | | | | | **outcome** | | | | | | | | **Nsnp** | | | | | | **Methods** | | | | | | | **Beta** | | | | | | | | **SE** | | | | | | | | **OR (95%CI)** | | | | | | | | ***P* value** | | | | | | | | **MR-PRESSO** | | | | | | | | **Heterogeneity** | | | | | | | | | | | | | | **Horizontal pleiotrop** | | | | | | | | | | | | | | | | | | | | | | | |
|  |  |  |  |  |  |  |  |  |  |  |  |  |  |  |  |  |  |  |  |  |  |  |  |  |  |  |  |  |  |  |  |  |  |  |  |  |  |  |  |  |  |  |  |  |  |  |  |  |  |  |  |  |  |  |  |  |  |  |  |  |  |  |  |  |  |  |  |  | **Cochran’s Q** | | | | | | | | ***P* value** | | | | | | **Egger intercept** | | | | | | | | **SE** | | | | | | | | ***P* value** | | | | | | | |
| phylum | Acute pancreatitis | | | | | | | Proteobacteria | | | | | | | | 7 | | | | | | MR-Egger | | | | | | | 0.28 | | | | | | | | 0.30 | | | | | | | | 1.33 (0.74-2.40) | | | | | | | | 0.388 | | | | | | | | 0.29 | | | | | | | | 7.662 | | | | | | | | 0.263 | | | | | | -0.031 | | | | | | | | 0.0439 | | | | | | | | 0.5112 | | | | | | | |
|  |  |  |  |  |  |  |  |  |  |  |  |  |  |  |  |  |  |  |  |  |  | Weighted median | | | | | | | 0.07 | | | | | | | | 0.04 | | | | | | | | 1.08 (0.99-1.18) | | | | | | | | 0.073 | | | | | | | |  |  |  |  |  |  |  |  |  |  |  |  |  |  |  |  |  |  |  |  |  |  |  |  |  |  |  |  |  |  |  |  |  |  |  |  |  |  |  |  |  |  |  |  |  |  |
|  |  |  |  |  |  |  |  |  |  |  |  |  |  |  |  |  |  |  |  |  |  | Inverse variance weighted | | | | | | | 0.07 | | | | | | | | 0.03 | | | | | | | | 1.08 (1.01-1.15) | | | | | | | | 0.038 | | | | | | | |  |  |  |  |  |  |  |  |  |  |  |  |  |  |  |  |  |  |  |  |  |  |  |  |  |  |  |  |  |  |  |  |  |  |  |  |  |  |  |  |  |  |  |  |  |  |
|  |  |  |  |  |  |  |  |  |  |  |  |  |  |  |  |  |  |  |  |  |  | Simple mode | | | | | | | 0.08 | | | | | | | | 0.06 | | | | | | | | 1.09 (0.97-1.23) | | | | | | | | 0.203 | | | | | | | |  |  |  |  |  |  |  |  |  |  |  |  |  |  |  |  |  |  |  |  |  |  |  |  |  |  |  |  |  |  |  |  |  |  |  |  |  |  |  |  |  |  |  |  |  |  |
|  |  |  |  |  |  |  |  |  |  |  |  |  |  |  |  |  |  |  |  |  |  | Weighted mode | | | | | | | 0.08 | | | | | | | | 0.05 | | | | | | | | 1.09 (0.97-1.23) | | | | | | | | 0.190 | | | | | | | |  |  |  |  |  |  |  |  |  |  |  |  |  |  |  |  |  |  |  |  |  |  |  |  |  |  |  |  |  |  |  |  |  |  |  |  |  |  |  |  |  |  |  |  |  |  |
| genus | Acute pancreatitis | | | | | | | Holdemania | | | | | | | | 7 | | | | | | MR-Egger | | | | | | | -0.40 | | | | | | | | 0.35 | | | | | | | | 0.67 (0.33-1.35) | | | | | | | | 0.312 | | | | | | | | 0.61 | | | | | | | | 4.81173 | | | | | | | | 0.5681 | | | | | | 0.0461 | | | | | | | | 0.0523 | | | | | | | | 0.418 | | | | | | | |
|  |  |  |  |  |  |  |  |  |  |  |  |  |  |  |  |  |  |  |  |  |  | Weighted median | | | | | | | -0.116 | | | | | | | | 0.05 | | | | | | | | 0.89 (0.80-1.00) | | | | | | | | 0.041 | | | | | | | |  |  |  |  |  |  |  |  |  |  |  |  |  |  |  |  |  |  |  |  |  |  |  |  |  |  |  |  |  |  |  |  |  |  |  |  |  |  |  |  |  |  |  |  |  |  |
|  |  |  |  |  |  |  |  |  |  |  |  |  |  |  |  |  |  |  |  |  |  | Inverse variance weighted | | | | | | | -0.089 | | | | | | | | 0.04 | | | | | | | | 0.91 (0.84-0.99) | | | | | | | | 0.043 | | | | | | | |  |  |  |  |  |  |  |  |  |  |  |  |  |  |  |  |  |  |  |  |  |  |  |  |  |  |  |  |  |  |  |  |  |  |  |  |  |  |  |  |  |  |  |  |  |  |
|  |  |  |  |  |  |  |  |  |  |  |  |  |  |  |  |  |  |  |  |  |  | Simple mode | | | | | | | -0.12 | | | | | | | | 0.07 | | | | | | | | 0.88 (0.76-1.03 | | | | | | | | 0.163 | | | | | | | |  |  |  |  |  |  |  |  |  |  |  |  |  |  |  |  |  |  |  |  |  |  |  |  |  |  |  |  |  |  |  |  |  |  |  |  |  |  |  |  |  |  |  |  |  |  |
|  |  |  |  |  |  |  |  |  |  |  |  |  |  |  |  |  |  |  |  |  |  | Weighted mode | | | | | | | -0.124 | | | | | | | | 0.07 | | | | | | | | 0.88 (0.77-1.02) | | | | | | | | 0.134 | | | | | | | |  |  |  |  |  |  |  |  |  |  |  |  |  |  |  |  |  |  |  |  |  |  |  |  |  |  |  |  |  |  |  |  |  |  |  |  |  |  |  |  |  |  |  |  |  |  |
| genus | Acute pancreatitis | | | | | | | Lachnospiraceae NC2004 group | | | | | | | | 7 | | | | | | MR-Egger | | | | | | | 0.186 | | | | | | | | 0.440 | | | | | | | | 1.20 (0.51-2.86) | | | | | | | | 0.689 | | | | | | | | 0.72 | | | | | | | | 3.346 | | | | | | | | 0.7642 | | | | | | -0.0070 | | | | | | | | 0.0640 | | | | | | | | 0.9165 | | | | | | | |
|  |  |  |  |  |  |  |  |  |  |  |  |  |  |  |  |  |  |  |  |  |  | Weighted median | | | | | | | 0.127 | | | | | | | | 0.072 | | | | | | | | 1.14 (0.99-1.31) | | | | | | | | 0.078 | | | | | | | |  |  |  |  |  |  |  |  |  |  |  |  |  |  |  |  |  |  |  |  |  |  |  |  |  |  |  |  |  |  |  |  |  |  |  |  |  |  |  |  |  |  |  |  |  |  |
|  |  |  |  |  |  |  |  |  |  |  |  |  |  |  |  |  |  |  |  |  |  | Inverse variance weighted | | | | | | | 0.138 | | | | | | | | 0.053 | | | | | | | | 1.15 (1.03-1.28) | | | | | | | | 0.010 | | | | | | | |  |  |  |  |  |  |  |  |  |  |  |  |  |  |  |  |  |  |  |  |  |  |  |  |  |  |  |  |  |  |  |  |  |  |  |  |  |  |  |  |  |  |  |  |  |  |
|  |  |  |  |  |  |  |  |  |  |  |  |  |  |  |  |  |  |  |  |  |  | Simple mode | | | | | | | 0.111 | | | | | | | | 0.103 | | | | | | | | 1.12 (0.91-1.37) | | | | | | | | 0.3244 | | | | | | | |  |  |  |  |  |  |  |  |  |  |  |  |  |  |  |  |  |  |  |  |  |  |  |  |  |  |  |  |  |  |  |  |  |  |  |  |  |  |  |  |  |  |  |  |  |  |
|  |  |  |  |  |  |  |  |  |  |  |  |  |  |  |  |  |  |  |  |  |  | Weighted mode | | | | | | | 0.106 | | | | | | | | 0.105 | | | | | | | | 1.11 (0.90-1.37) | | | | | | | | 0.3531 | | | | | | | |  |  |  |  |  |  |  |  |  |  |  |  |  |  |  |  |  |  |  |  |  |  |  |  |  |  |  |  |  |  |  |  |  |  |  |  |  |  |  |  |  |  |  |  |  |  |
| genus | Acute pancreatitis | | | | | | | Marvinbryantia | | | | | | | | 7 | | | | | | MR-Egger | | | | | | | -0.02 | | | | | | | | 0.37 | | | | | | | | 0.97 (0.46-2.04) | | | | | | | | 0.944 | | | | | | | | 0.25 | | | | | | | | 7.408 | | | | | | | | 0.284 | | | | | | 0.018 | | | | | | | | 0.054 | | | | | | | | 0.755 | | | | | | | |
|  |  |  |  |  |  |  |  |  |  |  |  |  |  |  |  |  |  |  |  |  |  | Weighted median | | | | | | | 0.05 | | | | | | | | 0.05 | | | | | | | | 1.05 (0.94-1.17) | | | | | | | | 0.357 | | | | | | | |  |  |  |  |  |  |  |  |  |  |  |  |  |  |  |  |  |  |  |  |  |  |  |  |  |  |  |  |  |  |  |  |  |  |  |  |  |  |  |  |  |  |  |  |  |  |
|  |  |  |  |  |  |  |  |  |  |  |  |  |  |  |  |  |  |  |  |  |  | Inverse variance weighted | | | | | | | 0.09 | | | | | | | | 0.04 | | | | | | | | 1.10 (1.01-1.20) | | | | | | | | 0.024 | | | | | | | |  |  |  |  |  |  |  |  |  |  |  |  |  |  |  |  |  |  |  |  |  |  |  |  |  |  |  |  |  |  |  |  |  |  |  |  |  |  |  |  |  |  |  |  |  |  |
|  |  |  |  |  |  |  |  |  |  |  |  |  |  |  |  |  |  |  |  |  |  | Simple mode | | | | | | | 0.04 | | | | | | | | 0.07 | | | | | | | | 1.05 (0.91-1.21) | | | | | | | | 0.553 | | | | | | | |  |  |  |  |  |  |  |  |  |  |  |  |  |  |  |  |  |  |  |  |  |  |  |  |  |  |  |  |  |  |  |  |  |  |  |  |  |  |  |  |  |  |  |  |  |  |
|  |  |  |  |  |  |  |  |  |  |  |  |  |  |  |  |  |  |  |  |  |  | Weighted mode | | | | | | | 0.04 | | | | | | | | 0.07 | | | | | | | | 1.05 (0.90-1.21) | | | | | | | | 0.563 | | | | | | | |  |  |  |  |  |  |  |  |  |  |  |  |  |  |  |  |  |  |  |  |  |  |  |  |  |  |  |  |  |  |  |  |  |  |  |  |  |  |  |  |  |  |  |  |  |  |
| genus | Acute pancreatitis | | | | | | | Oscillospira | | | | | | | | 7 | | | | | | MR-Egger | | | | | | | 0.29 | | | | | | | | 0.34 | | | | | | | | 1.34 (0.69-2.62) | | | | | | | | 0.428 | | | | | | | | 0.55 | | | | | | | | 5.367 | | | | | | | | 0.4975 | | | | | | -0.055 | | | | | | | | 0.049 | | | | | | | | 0.315 | | | | | | | |
|  |  |  |  |  |  |  |  |  |  |  |  |  |  |  |  |  |  |  |  |  |  | Weighted median | | | | | | | -0.04 | | | | | | | | 0.05 | | | | | | | | 0.96 (0.86-1.07) | | | | | | | | 0.439 | | | | | | | |  |  |  |  |  |  |  |  |  |  |  |  |  |  |  |  |  |  |  |  |  |  |  |  |  |  |  |  |  |  |  |  |  |  |  |  |  |  |  |  |  |  |  |  |  |  |
|  |  |  |  |  |  |  |  |  |  |  |  |  |  |  |  |  |  |  |  |  |  | Inverse variance weighted | | | | | | | -0.08 | | | | | | | | 0.04 | | | | | | | | 0.92 (0.85-1.00) | | | | | | | | 0.045 | | | | | | | |  |  |  |  |  |  |  |  |  |  |  |  |  |  |  |  |  |  |  |  |  |  |  |  |  |  |  |  |  |  |  |  |  |  |  |  |  |  |  |  |  |  |  |  |  |  |
|  |  |  |  |  |  |  |  |  |  |  |  |  |  |  |  |  |  |  |  |  |  | Simple mode | | | | | | | -0.02 | | | | | | | | 0.09 | | | | | | | | 0.98 (0.82-1.17) | | | | | | | | 0.805 | | | | | | | |  |  |  |  |  |  |  |  |  |  |  |  |  |  |  |  |  |  |  |  |  |  |  |  |  |  |  |  |  |  |  |  |  |  |  |  |  |  |  |  |  |  |  |  |  |  |
|  |  |  |  |  |  |  |  |  |  |  |  |  |  |  |  |  |  |  |  |  |  | Weighted mode | | | | | | | -0.02 | | | | | | | | 0.07 | | | | | | | | 0.98 (0.85-1.13) | | | | | | | | 0.763 | | | | | | | |  |  |  |  |  |  |  |  |  |  |  |  |  |  |  |  |  |  |  |  |  |  |  |  |  |  |  |  |  |  |  |  |  |  |  |  |  |  |  |  |  |  |  |  |  |  |
| **Supplementary Table 3:MR results and sensitivity analysis of gut microbiota on CP** | | | | | | | | | | | | | | | | | | | | | | | | | | | | | | | | | | | | | | | | | | | | | | | | | | | | | | | | | | | | | | | | | | | | | | | | | | | | | | | | | | | | | | | | | | | | | | | | | | | | | | | | | | |
| **Taxa** | | | | | | | **Gut microbiota (exposure)** | | | | | | **Trait (outcome)** | | | | | | | | **Nsnp** | | | | | | | **Methods** | | | | | | | | **Beta** | | | | | | | | **SE** | | | | | | | | **OR (95%CI)** | | | | | | | | ***P* value** | | | | | | | | **MR-PRESSO** | | | | | | | | **Heterogeneity** | | | | | | | | | | | | | | **Horizontal pleiotrop** | | | | | | | | | | | | | | | | |
|  |  |  |  |  |  |  |  |  |  |  |  |  |  |  |  |  |  |  |  |  |  |  |  |  |  |  |  |  |  |  |  |  |  |  |  |  |  |  |  |  |  |  |  |  |  |  |  |  |  |  |  |  |  |  |  |  |  |  |  |  |  |  |  |  |  |  |  |  |  |  |  |  |  |  |  | **Cochran’s Q** | | | | | | | ***P* value** | | | | | | | **Egger intercept** | | | | | | | | **SE** | | | | | | | | ***P* value** |
| Family | | | | | | | Defluviitaleaceae | | | | | | Chronic pancreatitis | | | | | | | | 11 | | | | | | | MR-Egger | | | | | | | | 0.37 | | | | | | | | 0.51 | | | | | | | | 1.45 (0.54-3.91) | | | | | | | | 0.484 | | | | | | | | 0.6 | | | | | | | | 8.751 | | | | | | | 0.460 | | | | | | | 0.036 | | | | | | | | 0.0516 | | | | | | | | 0.9566 |
|  |  |  |  |  |  |  |  |  |  |  |  |  |  |  |  |  |  |  |  |  |  |  |  |  |  |  |  | Weighted median | | | | | | | | 0.17 | | | | | | | | 0.21 | | | | | | | | 1.19 (0.80-1.78) | | | | | | | | 0.398 | | | | | | | |  |  |  |  |  |  |  |  |  |  |  |  |  |  |  |  |  |  |  |  |  |  |  |  |  |  |  |  |  |  |  |  |  |  |  |  |  |  |  |
|  |  |  |  |  |  |  |  |  |  |  |  |  |  |  |  |  |  |  |  |  |  |  |  |  |  |  |  | Inverse variance weighted | | | | | | | | 0.34 | | | | | | | | 0.14 | | | | | | | | 1.41 (1.05-1.88) | | | | | | | | 0.021 | | | | | | | |  |  |  |  |  |  |  |  |  |  |  |  |  |  |  |  |  |  |  |  |  |  |  |  |  |  |  |  |  |  |  |  |  |  |  |  |  |  |  |
|  |  |  |  |  |  |  |  |  |  |  |  |  |  |  |  |  |  |  |  |  |  |  |  |  |  |  |  | Simple mode | | | | | | | | 0.04 | | | | | | | | 0.33 | | | | | | | | 1.05 (0.55-2.01) | | | | | | | | 0.888 | | | | | | | |  |  |  |  |  |  |  |  |  |  |  |  |  |  |  |  |  |  |  |  |  |  |  |  |  |  |  |  |  |  |  |  |  |  |  |  |  |  |  |
|  |  |  |  |  |  |  |  |  |  |  |  |  |  |  |  |  |  |  |  |  |  |  |  |  |  |  |  | Weighted mode | | | | | | | | 0.04 | | | | | | | | 0.34 | | | | | | | | 1.05 (0.53-2.07) | | | | | | | | 0.893 | | | | | | | |  |  |  |  |  |  |  |  |  |  |  |  |  |  |  |  |  |  |  |  |  |  |  |  |  |  |  |  |  |  |  |  |  |  |  |  |  |  |  |
| genus | | | | | | | Barnesiella | | | | | | Chronic pancreatitis | | | | | | | | 13 | | | | | | | MR-Egger | | | | | | | | 0.29 | | | | | | | | 0.70 | | | | | | | | 1.34 (0.33-5.38) | | | | | | | | 0.686 | | | | | | | | 0.72 | | | | | | | | 7.9779 | | | | | | | 0.7152 | | | | | | | 0.007 | | | | | | | | 0.0535 | | | | | | | | 0.8851 |
|  |  |  |  |  |  |  |  |  |  |  |  |  |  |  |  |  |  |  |  |  |  |  |  |  |  |  |  | Weighted median | | | | | | | | 0.19 | | | | | | | | 0.22 | | | | | | | | 1.21 (0.77-1.90) | | | | | | | | 0.402 | | | | | | | |  |  |  |  |  |  |  |  |  |  |  |  |  |  |  |  |  |  |  |  |  |  |  |  |  |  |  |  |  |  |  |  |  |  |  |  |  |  |  |
|  |  |  |  |  |  |  |  |  |  |  |  |  |  |  |  |  |  |  |  |  |  |  |  |  |  |  |  | Inverse variance weighted | | | | | | | | 0.39 | | | | | | | | 0.17 | | | | | | | | 1.48 (1.06-2.09) | | | | | | | | 0.023 | | | | | | | |  |  |  |  |  |  |  |  |  |  |  |  |  |  |  |  |  |  |  |  |  |  |  |  |  |  |  |  |  |  |  |  |  |  |  |  |  |  |  |
|  |  |  |  |  |  |  |  |  |  |  |  |  |  |  |  |  |  |  |  |  |  |  |  |  |  |  |  | Simple mode | | | | | | | | 0.09 | | | | | | | | 0.40 | | | | | | | | 1.10 (0.50-2.42) | | | | | | | | 0.814 | | | | | | | |  |  |  |  |  |  |  |  |  |  |  |  |  |  |  |  |  |  |  |  |  |  |  |  |  |  |  |  |  |  |  |  |  |  |  |  |  |  |  |
|  |  |  |  |  |  |  |  |  |  |  |  |  |  |  |  |  |  |  |  |  |  |  |  |  |  |  |  | Weighted mode | | | | | | | | 0.10 | | | | | | | | 0.40 | | | | | | | | 1.11 (0.51-2.45) | | | | | | | | 0.795 | | | | | | | |  |  |  |  |  |  |  |  |  |  |  |  |  |  |  |  |  |  |  |  |  |  |  |  |  |  |  |  |  |  |  |  |  |  |  |  |  |  |  |
| genus | | | | | | | Defluviitaleaceae | | | | | | Chronic pancreatitis | | | | | | | | 9 | | | | | | | MR-Egger | | | | | | | | 0.30 | | | | | | | | 0.60 | | | | | | | | 1.36 (0.42-4.42) | | | | | | | | 0.624 | | | | | | | | 0.62 | | | | | | | | 6.927 | | | | | | | 0.544 | | | | | | | 0.0061 | | | | | | | | 0.0600 | | | | | | | | 0.9210 |
|  |  |  |  |  |  |  |  |  |  |  |  |  |  |  |  |  |  |  |  |  |  |  |  |  |  |  |  | Weighted median | | | | | | | | 0.34 | | | | | | | | 0.22 | | | | | | | | 1.41 (0.91-2.17) | | | | | | | | 0.123 | | | | | | | |  |  |  |  |  |  |  |  |  |  |  |  |  |  |  |  |  |  |  |  |  |  |  |  |  |  |  |  |  |  |  |  |  |  |  |  |  |  |  |
|  |  |  |  |  |  |  |  |  |  |  |  |  |  |  |  |  |  |  |  |  |  |  |  |  |  |  |  | Inverse variance weighted | | | | | | | | 0.36 | | | | | | | | 0.16 | | | | | | | | 1.44 (1.04-2.00) | | | | | | | | 0.027 | | | | | | | |  |  |  |  |  |  |  |  |  |  |  |  |  |  |  |  |  |  |  |  |  |  |  |  |  |  |  |  |  |  |  |  |  |  |  |  |  |  |  |
|  |  |  |  |  |  |  |  |  |  |  |  |  |  |  |  |  |  |  |  |  |  |  |  |  |  |  |  | Simple mode | | | | | | | | 0.15 | | | | | | | | 0.38 | | | | | | | | 1.16 (0.55-2.46) | | | | | | | | 0.701 | | | | | | | |  |  |  |  |  |  |  |  |  |  |  |  |  |  |  |  |  |  |  |  |  |  |  |  |  |  |  |  |  |  |  |  |  |  |  |  |  |  |  |
|  |  |  |  |  |  |  |  |  |  |  |  |  |  |  |  |  |  |  |  |  |  |  |  |  |  |  |  | Weighted mode | | | | | | | | 0.15 | | | | | | | | 0.37 | | | | | | | | 1.21 (0.58-2.50) | | | | | | | | 0.627 | | | | | | | |  |  |  |  |  |  |  |  |  |  |  |  |  |  |  |  |  |  |  |  |  |  |  |  |  |  |  |  |  |  |  |  |  |  |  |  |  |  |  |
| genus | | | | | | | Eubacterium xylanophilum group | | | | | | Chronic pancreatitis | | | | | | | | 9 | | | | | | | MR-Egger | | | | | | | | 0.51 | | | | | | | | 0.54 | | | | | | | | 1.68 (0.57-4.88) | | | | | | | | 0.375 | | | | | | | | 0.68 | | | | | | | | 5.9540 | | | | | | | 0.6523 | | | | | | | -0.0094 | | | | | | | | 0.044 | | | | | | | | 0.838 |
|  |  |  |  |  |  |  |  |  |  |  |  |  |  |  |  |  |  |  |  |  |  |  |  |  |  |  |  | Weighted median | | | | | | | | 0.54 | | | | | | | | 0.24 | | | | | | | | 1.72 (1.07-2.78) | | | | | | | | 0.025 | | | | | | | |  |  |  |  |  |  |  |  |  |  |  |  |  |  |  |  |  |  |  |  |  |  |  |  |  |  |  |  |  |  |  |  |  |  |  |  |  |  |  |
|  |  |  |  |  |  |  |  |  |  |  |  |  |  |  |  |  |  |  |  |  |  |  |  |  |  |  |  | Inverse variance weighted | | | | | | | | 0.40 | | | | | | | | 0.18 | | | | | | | | 1.50 (1.05-2.15) | | | | | | | | 0.026 | | | | | | | |  |  |  |  |  |  |  |  |  |  |  |  |  |  |  |  |  |  |  |  |  |  |  |  |  |  |  |  |  |  |  |  |  |  |  |  |  |  |  |
|  |  |  |  |  |  |  |  |  |  |  |  |  |  |  |  |  |  |  |  |  |  |  |  |  |  |  |  | Simple mode | | | | | | | | 0.61 | | | | | | | | 0.39 | | | | | | | | 1.85 (0.86-4.02) | | | | | | | | 0.156 | | | | | | | |  |  |  |  |  |  |  |  |  |  |  |  |  |  |  |  |  |  |  |  |  |  |  |  |  |  |  |  |  |  |  |  |  |  |  |  |  |  |  |
|  |  |  |  |  |  |  |  |  |  |  |  |  |  |  |  |  |  |  |  |  |  |  |  |  |  |  |  | Weighted mode | | | | | | | | 0.61 | | | | | | | | 0.40 | | | | | | | | 1.85 (0.84-4.11) | | | | | | | | 0.166 | | | | | | | |  |  |  |  |  |  |  |  |  |  |  |  |  |  |  |  |  |  |  |  |  |  |  |  |  |  |  |  |  |  |  |  |  |  |  |  |  |  |  |
| genus | | | | | | | Sellimonas | | | | | | Chronic pancreatitis | | | | | | | | 9 | | | | | | | MR-Egger | | | | | | | | -0.51 | | | | | | | | 0.55 | | | | | | | | 0.60 (0.20-1.79) | | | | | | | | 0.389 | | | | | | | | 0.39 | | | | | | | | 8.5010 | | | | | | | 0.3861 | | | | | | | 0.1039 | | | | | | | | 0.080 | | | | | | | | 0.236 |
|  |  |  |  |  |  |  |  |  |  |  |  |  |  |  |  |  |  |  |  |  |  |  |  |  |  |  |  | Weighted median | | | | | | | | 0.15 | | | | | | | | 0.12 | | | | | | | | 1.17 (0.90-1.50) | | | | | | | | 0.234 | | | | | | | |  |  |  |  |  |  |  |  |  |  |  |  |  |  |  |  |  |  |  |  |  |  |  |  |  |  |  |  |  |  |  |  |  |  |  |  |  |  |  |
|  |  |  |  |  |  |  |  |  |  |  |  |  |  |  |  |  |  |  |  |  |  |  |  |  |  |  |  | Inverse variance weighted | | | | | | | | 0.19 | | | | | | | | 0.09 | | | | | | | | 1.22 (1.01-1.48) | | | | | | | | 0.042 | | | | | | | |  |  |  |  |  |  |  |  |  |  |  |  |  |  |  |  |  |  |  |  |  |  |  |  |  |  |  |  |  |  |  |  |  |  |  |  |  |  |  |
|  |  |  |  |  |  |  |  |  |  |  |  |  |  |  |  |  |  |  |  |  |  |  |  |  |  |  |  | Simple mode | | | | | | | | 0.07 | | | | | | | | 0.22 | | | | | | | | 1.08 (0.69-1.68) | | | | | | | | 0.837 | | | | | | | |  |  |  |  |  |  |  |  |  |  |  |  |  |  |  |  |  |  |  |  |  |  |  |  |  |  |  |  |  |  |  |  |  |  |  |  |  |  |  |
|  |  |  |  |  |  |  |  |  |  |  |  |  |  |  |  |  |  |  |  |  |  |  |  |  |  |  |  | Weighted mode | | | | | | | | 0.04 | | | | | | | | 0.23 | | | | | | | | 1.05 (0.67-1.65) | | | | | | | |  | | | | | | | |  |  |  |  |  |  |  |  |  |  |  |  |  |  |  |  |  |  |  |  |  |  |  |  |  |  |  |  |  |  |  |  |  |  |  |  |  |  |  |
| genus | | | | | | | Slackia | | | | | | Chronic pancreatitis | | | | | | | | 6 | | | | | | | MR-Egger | | | | | | | | 1.088 | | | | | | | | 1.13 | | | | | | | | 2.97 (0.32-27.61) | | | | | | | | 0.393 | | | | | | | | 0.61 | | | | | | | | 3.3418 | | | | | | | 0.166 | | | | | | | -0.1555 | | | | | | | | 0.113 | | | | | | | | 0.241 |
|  |  |  |  |  |  |  |  |  |  |  |  |  |  |  |  |  |  |  |  |  |  |  |  |  |  |  |  | Weighted median | | | | | | | | -0.43 | | | | | | | | 0.21 | | | | | | | | 0.65  (0.43- 0.99) | | | | | | | | 0.044 | | | | | | | |  |  |  |  |  |  |  |  |  |  |  |  |  |  |  |  |  |  |  |  |  |  |  |  |  |  |  |  |  |  |  |  |  |  |  |  |  |  |  |
|  |  |  |  |  |  |  |  |  |  |  |  |  |  |  |  |  |  |  |  |  |  |  |  |  |  |  |  | Inverse variance weighted | | | | | | | | -0.45 | | | | | | | | 0.17 | | | | | | | | 0.63  (0.45- 0.89) | | | | | | | | 0.009 | | | | | | | |  |  |  |  |  |  |  |  |  |  |  |  |  |  |  |  |  |  |  |  |  |  |  |  |  |  |  |  |  |  |  |  |  |  |  |  |  |  |  |
|  |  |  |  |  |  |  |  |  |  |  |  |  |  |  |  |  |  |  |  |  |  |  |  |  |  |  |  | Simple mode | | | | | | | | -0.65 | | | | | | | | 0.32 | | | | | | | | 0.52  (0.28- 0.97) | | | | | | | | 0.096 | | | | | | | |  |  |  |  |  |  |  |  |  |  |  |  |  |  |  |  |  |  |  |  |  |  |  |  |  |  |  |  |  |  |  |  |  |  |  |  |  |  |  |
|  |  |  |  |  |  |  |  |  |  |  |  |  |  |  |  |  |  |  |  |  |  |  |  |  |  |  |  | Weighted mode | | | | | | | | -0.24 | | | | | | | | 0.32 | | | | | | | | 0.78  (0.41- 1.48) | | | | | | | | 0.488 | | | | | | | |  |  |  |  |  |  |  |  |  |  |  |  |  |  |  |  |  |  |  |  |  |  |  |  |  |  |  |  |  |  |  |  |  |  |  |  |  |  |  |
| **Supplementary Table 4:In reverse MR results and sensitivity analysis of CP on gut microbiota** | | | | | | | | | | | | | | | | | | | | | | | | | | | | | | | | | | | | | | | | | | | | | | | | | | | | | | | | | | | | | | | | | | | | | | | | | | | | | | | | | | | | | | | | | | | | | | | | | | | | | | | | | | |
| **Taxa** | | **exposure** | | | | | | | **outcome** | | | | | | | | **Nsnp** | | | | | | **Methods** | | | | | | | **Beta** | | | | | | | | **SE** | | | | | | | | **OR (95%CI)** | | | | | | | | ***P* value** | | | | | | | | **MR-PRESSO** | | | | | | | | **Heterogeneity** | | | | | | | | | | | | | | **Horizontal pleiotrop** | | | | | | | | | | | | | | | | | | | | | | |
|  |  |  |  |  |  |  |  |  |  |  |  |  |  |  |  |  |  |  |  |  |  |  |  |  |  |  |  |  |  |  |  |  |  |  |  |  |  |  |  |  |  |  |  |  |  |  |  |  |  |  |  |  |  |  |  |  |  |  |  |  |  |  |  |  |  |  |  |  |  | **Cochran’s Q** | | | | | | | | ***P* value** | | | | | | **Egger intercept** | | | | | | | | **SE** | | | | | | | | ***P* value** | | | | | | |
| phylum | | Chronic pancreatitis | | | | | | | Firmicutes | | | | | | | | 16 | | | | | | MR-Egger | | | | | | | 0.01 | | | | | | | | 0.05 | | | | | | | | 1.01 (0.91-1.11) | | | | | | | | 0.851 | | | | | | | | 0.66 | | | | | | | | 11.96 | | | | | | | | 0.681 | | | | | | -0.0088 | | | | | | | | 0.0099 | | | | | | | | 0.388591 | | | | | | |
|  |  |  |  |  |  |  |  |  |  |  |  |  |  |  |  |  |  |  |  |  |  |  | Weighted median | | | | | | | -0.01 | | | | | | | | 0.02 | | | | | | | | 0.98 (0.94-1.03) | | | | | | | | 0.418 | | | | | | | |  |  |  |  |  |  |  |  |  |  |  |  |  |  |  |  |  |  |  |  |  |  |  |  |  |  |  |  |  |  |  |  |  |  |  |  |  |  |  |  |  |  |  |  |  |
|  |  |  |  |  |  |  |  |  |  |  |  |  |  |  |  |  |  |  |  |  |  |  | Inverse variance weighted | | | | | | | -0.03 | | | | | | | | 0.01 | | | | | | | | 0.97 (0.94-1.00) | | | | | | | | 0.047 | | | | | | | |  |  |  |  |  |  |  |  |  |  |  |  |  |  |  |  |  |  |  |  |  |  |  |  |  |  |  |  |  |  |  |  |  |  |  |  |  |  |  |  |  |  |  |  |  |
|  |  |  |  |  |  |  |  |  |  |  |  |  |  |  |  |  |  |  |  |  |  |  | Simple mode | | | | | | | 0.0002 | | | | | | | | 0.04 | | | | | | | | 1.00 (0.92-1.09) | | | | | | | | 0.996 | | | | | | | |  |  |  |  |  |  |  |  |  |  |  |  |  |  |  |  |  |  |  |  |  |  |  |  |  |  |  |  |  |  |  |  |  |  |  |  |  |  |  |  |  |  |  |  |  |
|  |  |  |  |  |  |  |  |  |  |  |  |  |  |  |  |  |  |  |  |  |  |  | Weighted mode | | | | | | | 0.004 | | | | | | | | 0.03 | | | | | | | | 1.00 (0.93-1.09) | | | | | | | | 0.913 | | | | | | | |  |  |  |  |  |  |  |  |  |  |  |  |  |  |  |  |  |  |  |  |  |  |  |  |  |  |  |  |  |  |  |  |  |  |  |  |  |  |  |  |  |  |  |  |  |
| Family | | Chronic pancreatitis | | | | | | | Family XIII | | | | | | | | 15 | | | | | | MR-Egger | | | | | | | -0.18 | | | | | | | | 0.10 | | | | | | | | 0.83 (0.68-1.02) | | | | | | | | 0.104 | | | | | | | | 0.72 | | | | | | | | 11.978 | | | | | | | | 0.6080 | | | | | | 0.026 | | | | | | | | 0.019 | | | | | | | | 0.2037 | | | | | | |
|  |  |  |  |  |  |  |  |  |  |  |  |  |  |  |  |  |  |  |  |  |  |  | Weighted median | | | | | | | -0.01 | | | | | | | | 0.02 | | | | | | | | 0.98 (0.94-1.03) | | | | | | | | 0.491 | | | | | | | |  |  |  |  |  |  |  |  |  |  |  |  |  |  |  |  |  |  |  |  |  |  |  |  |  |  |  |  |  |  |  |  |  |  |  |  |  |  |  |  |  |  |  |  |  |
|  |  |  |  |  |  |  |  |  |  |  |  |  |  |  |  |  |  |  |  |  |  |  | Inverse variance weighted | | | | | | | -0.04 | | | | | | | | 0.01 | | | | | | | | 0.96 (0.92-0.99) | | | | | | | | 0.014 | | | | | | | |  |  |  |  |  |  |  |  |  |  |  |  |  |  |  |  |  |  |  |  |  |  |  |  |  |  |  |  |  |  |  |  |  |  |  |  |  |  |  |  |  |  |  |  |  |
|  |  |  |  |  |  |  |  |  |  |  |  |  |  |  |  |  |  |  |  |  |  |  | Simple mode | | | | | | | -0.004 | | | | | | | | 0.04 | | | | | | | | 1.00 (0.91-1.09) | | | | | | | | 0.915 | | | | | | | |  |  |  |  |  |  |  |  |  |  |  |  |  |  |  |  |  |  |  |  |  |  |  |  |  |  |  |  |  |  |  |  |  |  |  |  |  |  |  |  |  |  |  |  |  |
|  |  |  |  |  |  |  |  |  |  |  |  |  |  |  |  |  |  |  |  |  |  |  | Weighted mode | | | | | | | -0.004 | | | | | | | | 0.03 | | | | | | | | 1.11 (0.51-2.45) | | | | | | | | 0.900 | | | | | | | |  |  |  |  |  |  |  |  |  |  |  |  |  |  |  |  |  |  |  |  |  |  |  |  |  |  |  |  |  |  |  |  |  |  |  |  |  |  |  |  |  |  |  |  |  |
| genus | | Chronic pancreatitis | | | | | | | Coprobacter | | | | | | | | 15 | | | | | | MR-Egger | | | | | | | 0.127 | | | | | | | | 0.14 | | | | | | | | 1.14 (0.85-1.51) | | | | | | | | 0.396 | | | | | | | | 0.95 | | | | | | | | 7.372 | | | | | | | | 0.919 | | | | | | -0.035 | | | | | | | | 0.0271 | | | | | | | | 0.2086 | | | | | | |
|  |  |  |  |  |  |  |  |  |  |  |  |  |  |  |  |  |  |  |  |  |  |  | Weighted median | | | | | | | -0.05 | | | | | | | | 0.03 | | | | | | | | 0.95 (0.88-1.02) | | | | | | | | 0.179 | | | | | | | |  |  |  |  |  |  |  |  |  |  |  |  |  |  |  |  |  |  |  |  |  |  |  |  |  |  |  |  |  |  |  |  |  |  |  |  |  |  |  |  |  |  |  |  |  |
|  |  |  |  |  |  |  |  |  |  |  |  |  |  |  |  |  |  |  |  |  |  |  | Inverse variance weighted | | | | | | | -0.06 | | | | | | | | 0.02 | | | | | | | | 0.94 (0.89-1.00) | | | | | | | | 0.032 | | | | | | | |  |  |  |  |  |  |  |  |  |  |  |  |  |  |  |  |  |  |  |  |  |  |  |  |  |  |  |  |  |  |  |  |  |  |  |  |  |  |  |  |  |  |  |  |  |
|  |  |  |  |  |  |  |  |  |  |  |  |  |  |  |  |  |  |  |  |  |  |  | Simple mode | | | | | | | -0.002 | | | | | | | | 0.07 | | | | | | | | 1.00 (0.87-1.14) | | | | | | | | 0.972 | | | | | | | |  |  |  |  |  |  |  |  |  |  |  |  |  |  |  |  |  |  |  |  |  |  |  |  |  |  |  |  |  |  |  |  |  |  |  |  |  |  |  |  |  |  |  |  |  |
|  |  |  |  |  |  |  |  |  |  |  |  |  |  |  |  |  |  |  |  |  |  |  | Weighted mode | | | | | | | 0.0004 | | | | | | | | 0.06 | | | | | | | | 1.00 (0.88-1.14) | | | | | | | | 0.994 | | | | | | | |  |  |  |  |  |  |  |  |  |  |  |  |  |  |  |  |  |  |  |  |  |  |  |  |  |  |  |  |  |  |  |  |  |  |  |  |  |  |  |  |  |  |  |  |  |
| genus | | Chronic pancreatitis | | | | | | | Eubacterium hallii group | | | | | | | | 15 | | | | | | MR-Egger | | | | | | | 0.02 | | | | | | | | 0.10 | | | | | | | | 1.03 (0.83-1.27) | | | | | | | | 0.815 | | | | | | | | 0.75 | | | | | | | | 10.256 | | | | | | | | 0.743 | | | | | | -0.0139 | | | | | | | | 0.0198 | | | | | | | | 0.4938 | | | | | | |
|  |  |  |  |  |  |  |  |  |  |  |  |  |  |  |  |  |  |  |  |  |  |  | Weighted median | | | | | | | -0.04 | | | | | | | | 0.02 | | | | | | | | 0.95 (0.91-1.00) | | | | | | | | 0.058 | | | | | | | |  |  |  |  |  |  |  |  |  |  |  |  |  |  |  |  |  |  |  |  |  |  |  |  |  |  |  |  |  |  |  |  |  |  |  |  |  |  |  |  |  |  |  |  |  |
|  |  |  |  |  |  |  |  |  |  |  |  |  |  |  |  |  |  |  |  |  |  |  | Inverse variance weighted | | | | | | | -0.048 | | | | | | | | 0.01 | | | | | | | | 0.95 (0.92-0.99) | | | | | | | | 0.008 | | | | | | | |  |  |  |  |  |  |  |  |  |  |  |  |  |  |  |  |  |  |  |  |  |  |  |  |  |  |  |  |  |  |  |  |  |  |  |  |  |  |  |  |  |  |  |  |  |
|  |  |  |  |  |  |  |  |  |  |  |  |  |  |  |  |  |  |  |  |  |  |  | Simple mode | | | | | | | -0.061 | | | | | | | | 0.04 | | | | | | | | 0.94 (0.86-1.03) | | | | | | | | 0.189 | | | | | | | |  |  |  |  |  |  |  |  |  |  |  |  |  |  |  |  |  |  |  |  |  |  |  |  |  |  |  |  |  |  |  |  |  |  |  |  |  |  |  |  |  |  |  |  |  |
|  |  |  |  |  |  |  |  |  |  |  |  |  |  |  |  |  |  |  |  |  |  |  | Weighted mode | | | | | | | -0.07 | | | | | | | | 0.04 | | | | | | | | 0.93 (0.85-1.02) | | | | | | | | 0.140 | | | | | | | |  |  |  |  |  |  |  |  |  |  |  |  |  |  |  |  |  |  |  |  |  |  |  |  |  |  |  |  |  |  |  |  |  |  |  |  |  |  |  |  |  |  |  |  |  |
| genus | | Chronic pancreatitis | | | | | | | Fusicatenibacter | | | | | | | | 15 | | | | | | MR-Egger | | | | | | | -0.11 | | | | | | | | 0.10 | | | | | | | | 0.89 (0.73-1.09) | | | | | | | | 0.271 | | | | | | | | 0.47 | | | | | | | | 13.31 | | | | | | | | 0.5015 | | | | | | 0.0136 | | | | | | | | 0.019 | | | | | | | | 0.4865 | | | | | | |
|  |  |  |  |  |  |  |  |  |  |  |  |  |  |  |  |  |  |  |  |  |  |  | Weighted median | | | | | | | -0.05 | | | | | | | | 0.02 | | | | | | | | 0.95 (0.90-1.00) | | | | | | | | 0.042 | | | | | | | |  |  |  |  |  |  |  |  |  |  |  |  |  |  |  |  |  |  |  |  |  |  |  |  |  |  |  |  |  |  |  |  |  |  |  |  |  |  |  |  |  |  |  |  |  |
|  |  |  |  |  |  |  |  |  |  |  |  |  |  |  |  |  |  |  |  |  |  |  | Inverse variance weighted | | | | | | | -0.04 | | | | | | | | 0.01 | | | | | | | | 0.96 (0.92-0.99) | | | | | | | | 0.011 | | | | | | | |  |  |  |  |  |  |  |  |  |  |  |  |  |  |  |  |  |  |  |  |  |  |  |  |  |  |  |  |  |  |  |  |  |  |  |  |  |  |  |  |  |  |  |  |  |
|  |  |  |  |  |  |  |  |  |  |  |  |  |  |  |  |  |  |  |  |  |  |  | Simple mode | | | | | | | -0.06 | | | | | | | | 0.04 | | | | | | | | 0.93 (0.85-1.02) | | | | | | | | 0.164 | | | | | | | |  |  |  |  |  |  |  |  |  |  |  |  |  |  |  |  |  |  |  |  |  |  |  |  |  |  |  |  |  |  |  |  |  |  |  |  |  |  |  |  |  |  |  |  |  |
|  |  |  |  |  |  |  |  |  |  |  |  |  |  |  |  |  |  |  |  |  |  |  | Weighted mode | | | | | | | -0.06 | | | | | | | | 0.04 | | | | | | | | 0.94 (0.86-1.02) | | | | | | | | 0.167 | | | | | | | |  |  |  |  |  |  |  |  |  |  |  |  |  |  |  |  |  |  |  |  |  |  |  |  |  |  |  |  |  |  |  |  |  |  |  |  |  |  |  |  |  |  |  |  |  |
| genus | | Chronic pancreatitis | | | | | | | Prevotella9 | | | | | | | | 15 | | | | | | MR-Egger | | | | | | | 0.160 | | | | | | | | 0.12 | | | | | | | | 2.97 (0.32-27.61) | | | | | | | | 0.231 | | | | | | | | 0.66 | | | | | | | | 10.43 | | | | | | | | 0.729 | | | | | | -0.018 | | | | | | | | 0.0237 | | | | | | | | 0.459 | | | | | | |
|  |  |  |  |  |  |  |  |  |  |  |  |  |  |  |  |  |  |  |  |  |  |  | Weighted median | | | | | | | 0.031 | | | | | | | | 0.03 | | | | | | | | 0.65  (0.43- 0.99) | | | | | | | | 0.324 | | | | | | | |  |  |  |  |  |  |  |  |  |  |  |  |  |  |  |  |  |  |  |  |  |  |  |  |  |  |  |  |  |  |  |  |  |  |  |  |  |  |  |  |  |  |  |  |  |
|  |  |  |  |  |  |  |  |  |  |  |  |  |  |  |  |  |  |  |  |  |  |  | Inverse variance weighted | | | | | | | 0.064 | | | | | | | | 0.023 | | | | | | | | 0.63  (0.45- 0.89) | | | | | | | | 0.006 | | | | | | | |  |  |  |  |  |  |  |  |  |  |  |  |  |  |  |  |  |  |  |  |  |  |  |  |  |  |  |  |  |  |  |  |  |  |  |  |  |  |  |  |  |  |  |  |  |
|  |  |  |  |  |  |  |  |  |  |  |  |  |  |  |  |  |  |  |  |  |  |  | Simple mode | | | | | | | 0.011 | | | | | | | | 0.04 | | | | | | | | 0.52  (0.28- 0.97) | | | | | | | | 0.826 | | | | | | | |  |  |  |  |  |  |  |  |  |  |  |  |  |  |  |  |  |  |  |  |  |  |  |  |  |  |  |  |  |  |  |  |  |  |  |  |  |  |  |  |  |  |  |  |  |
|  |  |  |  |  |  |  |  |  |  |  |  |  |  |  |  |  |  |  |  |  |  |  | Weighted mode | | | | | | | 0.010 | | | | | | | | 0.05 | | | | | | | | 0.78  (0.41- 1.48) | | | | | | | | 0.841 | | | | | | | |  |  |  |  |  |  |  |  |  |  |  |  |  |  |  |  |  |  |  |  |  |  |  |  |  |  |  |  |  |  |  |  |  |  |  |  |  |  |  |  |  |  |  |  |  |
| genus | | Chronic pancreatitis | | | | | | | Slackia | | | | | | | | 15 | | | | | | MR-Egger | | | | | | | -0.26 | | | | | | | | 0.151 | | | | | | | | 0.77 (0.57-1.03) | | | | | | | | 0.101 | | | | | | | | 0.9 | | | | | | | | 7.656 | | | | | | | | 0.906 | | | | | | 0.0395 | | | | | | | | 0.0284 | | | | | | | | 0.18716 | | | | | | |
|  |  |  |  |  |  |  |  |  |  |  |  |  |  |  |  |  |  |  |  |  |  |  | Weighted median | | | | | | | -0.06 | | | | | | | | 0.041 | | | | | | | | 0.94 (0.86-1.01) | | | | | | | | 0.103 | | | | | | | |  |  |  |  |  |  |  |  |  |  |  |  |  |  |  |  |  |  |  |  |  |  |  |  |  |  |  |  |  |  |  |  |  |  |  |  |  |  |  |  |  |  |  |  |  |
|  |  |  |  |  |  |  |  |  |  |  |  |  |  |  |  |  |  |  |  |  |  |  | Inverse variance weighted | | | | | | | -0.060 | | | | | | | | 0.0298 | | | | | | | | 0.94 (0.89-1.00) | | | | | | | | 0.043 | | | | | | | |  |  |  |  |  |  |  |  |  |  |  |  |  |  |  |  |  |  |  |  |  |  |  |  |  |  |  |  |  |  |  |  |  |  |  |  |  |  |  |  |  |  |  |  |  |
|  |  |  |  |  |  |  |  |  |  |  |  |  |  |  |  |  |  |  |  |  |  |  | Simple mode | | | | | | | -0.11 | | | | | | | | 0.069 | | | | | | | | 0.89 (0.78-1.02) | | | | | | | | 0.127 | | | | | | | |  |  |  |  |  |  |  |  |  |  |  |  |  |  |  |  |  |  |  |  |  |  |  |  |  |  |  |  |  |  |  |  |  |  |  |  |  |  |  |  |  |  |  |  |  |
|  |  |  |  |  |  |  |  |  |  |  |  |  |  |  |  |  |  |  |  |  |  |  | Weighted mode | | | | | | | -0.10 | | | | | | | | 0.0687 | | | | | | | | 0.90 (0.79-1.03) | | | | | | | | 0.1506 | | | | | | | |  |  |  |  |  |  |  |  |  |  |  |  |  |  |  |  |  |  |  |  |  |  |  |  |  |  |  |  |  |  |  |  |  |  |  |  |  |  |  |  |  |  |  |  |  |
| **Supplementary Table 5**:MR results and sensitivity analysis of gut microbiota on AAP. | | | | | | | | | | | | | | | | | | | | | | | | | | | | | | | | | | | | | | | | | | | | | | | | | | | | | | | | | | | | | | | | | | | | | | | | | | | | | | | | | | | | | | | | | | | | | | | | | | | | | | | | | | |
| **Taxa** | | | **exposure** | | | | | | | **outcome** | | | | | | | | **Nsnp** | | | | | | **Methods** | | | | | | | **Beta** | | | | | | | | **SE** | | | | | | | | **OR (95%CI)** | | | | | | | | ***P* value** | | | | | | | | **MR-PRESSO** | | | | | | | | **Heterogeneity** | | | | | | | | | | | | | | **Horizontal pleiotrop** | | | | | | | | | | | | | | | | | | | | | |
|  |  |  |  |  |  |  |  |  |  |  |  |  |  |  |  |  |  |  |  |  |  |  |  |  |  |  |  |  |  |  |  |  |  |  |  |  |  |  |  |  |  |  |  |  |  |  |  |  |  |  |  |  |  |  |  |  |  |  |  |  |  |  |  |  |  |  |  |  |  |  | **Cochran’s Q** | | | | | | | | ***P* value** | | | | | | **Egger intercept** | | | | | | | | **SE** | | | | | | | | ***P* value** | | | | | |
| genus | | | Flavonifractor | | | | | | | Acohol-induced acute pancreatitis | | | | | | | | 5 | | | | | | MR-Egger | | | | | | | -3.55 | | | | | | | | 1.81 | | | | | | | | 0.03 (0.00-1.00) | | | | | | | | 0.144 | | | | | | | | 0.6 | | | | | | | | 3.484 | | | | | | | | 0.480 | | | | | | 0.1969 | | | | | | | | 0.149 | | | | | | | | 0.2780 | | | | | |
|  |  |  |  |  |  |  |  |  |  |  |  |  |  |  |  |  |  |  |  |  |  |  |  | Weighted median | | | | | | | -1.21 | | | | | | | | 0.61 | | | | | | | | 0.30 (0.09-0.99) | | | | | | | | 0.048 | | | | | | | |  |  |  |  |  |  |  |  |  |  |  |  |  |  |  |  |  |  |  |  |  |  |  |  |  |  |  |  |  |  |  |  |  |  |  |  |  |  |  |  |  |  |  |  |
|  |  |  |  |  |  |  |  |  |  |  |  |  |  |  |  |  |  |  |  |  |  |  |  | Inverse variance weighted | | | | | | | -1.23 | | | | | | | | 0.45 | | | | | | | | 0.29 (0.12-0.71) | | | | | | | | 0.006 | | | | | | | |  |  |  |  |  |  |  |  |  |  |  |  |  |  |  |  |  |  |  |  |  |  |  |  |  |  |  |  |  |  |  |  |  |  |  |  |  |  |  |  |  |  |  |  |
|  |  |  |  |  |  |  |  |  |  |  |  |  |  |  |  |  |  |  |  |  |  |  |  | Simple mode | | | | | | | -0.88 | | | | | | | | 0.86 | | | | | | | | 0.41 (0.08-2.26) | | | | | | | | 0.366 | | | | | | | |  |  |  |  |  |  |  |  |  |  |  |  |  |  |  |  |  |  |  |  |  |  |  |  |  |  |  |  |  |  |  |  |  |  |  |  |  |  |  |  |  |  |  |  |
|  |  |  |  |  |  |  |  |  |  |  |  |  |  |  |  |  |  |  |  |  |  |  |  | Weighted mode | | | | | | | -0.92 | | | | | | | | 0.80 | | | | | | | | 0.40 (0.08-1.93) | | | | | | | | 0.314 | | | | | | | |  |  |  |  |  |  |  |  |  |  |  |  |  |  |  |  |  |  |  |  |  |  |  |  |  |  |  |  |  |  |  |  |  |  |  |  |  |  |  |  |  |  |  |  |
| genus | | | Haemophilus | | | | | | | Acohol-induced acute pancreatitis | | | | | | | | 9 | | | | | | MR-Egger | | | | | | | 0.15 | | | | | | | | 0.62 | | | | | | | | 1.16 (0.34- 3.94) | | | | | | | | 0.816 | | | | | | | | 0.59 | | | | | | | | 6.239 | | | | | | | | 0.6204 | | | | | | 0.0544 | | | | | | | | 0.072 | | | | | | | | 0.479 | | | | | |
|  |  |  |  |  |  |  |  |  |  |  |  |  |  |  |  |  |  |  |  |  |  |  |  | Weighted median | | | | | | | 0.97 | | | | | | | | 0.36 | | | | | | | | 2.66 (1.29- 5.49) | | | | | | | | 0.007 | | | | | | | |  |  |  |  |  |  |  |  |  |  |  |  |  |  |  |  |  |  |  |  |  |  |  |  |  |  |  |  |  |  |  |  |  |  |  |  |  |  |  |  |  |  |  |  |
|  |  |  |  |  |  |  |  |  |  |  |  |  |  |  |  |  |  |  |  |  |  |  |  | Inverse variance weighted | | | | | | | 0.56 | | | | | | | | 0.27 | | | | | | | | 1.76 (1.02- 3.04) | | | | | | | | 0.041 | | | | | | | |  |  |  |  |  |  |  |  |  |  |  |  |  |  |  |  |  |  |  |  |  |  |  |  |  |  |  |  |  |  |  |  |  |  |  |  |  |  |  |  |  |  |  |  |
|  |  |  |  |  |  |  |  |  |  |  |  |  |  |  |  |  |  |  |  |  |  |  |  | Simple mode | | | | | | | 1.12 | | | | | | | | 0.64 | | | | | | | | 3.08 (0.87-10.98) | | | | | | | | 0.120 | | | | | | | |  |  |  |  |  |  |  |  |  |  |  |  |  |  |  |  |  |  |  |  |  |  |  |  |  |  |  |  |  |  |  |  |  |  |  |  |  |  |  |  |  |  |  |  |
|  |  |  |  |  |  |  |  |  |  |  |  |  |  |  |  |  |  |  |  |  |  |  |  | Weighted mode | | | | | | | 1.11 | | | | | | | | 0.70 | | | | | | | | 3.04 (0.76-12.20) | | | | | | | | 0.156 | | | | | | | |  |  |  |  |  |  |  |  |  |  |  |  |  |  |  |  |  |  |  |  |  |  |  |  |  |  |  |  |  |  |  |  |  |  |  |  |  |  |  |  |  |  |  |  |
| genus | | | Intestinimonas | | | | | | | Acohol-induced acute pancreatitis | | | | | | | | 16 | | | | | | MR-Egger | | | | | | | 1.16 | | | | | | | | 0.74 | | | | | | | | 3.20 (0.74-13.91) | | | | | | | | 0.142 | | | | | | | | 0.65 | | | | | | | | 12.136 | | | | | | | | 0.668 | | | | | | -0.056 | | | | | | | | 0.064 | | | | | | | | 0.391 | | | | | |
|  |  |  |  |  |  |  |  |  |  |  |  |  |  |  |  |  |  |  |  |  |  |  |  | Weighted median | | | | | | | 0.84 | | | | | | | | 0.37 | | | | | | | | 2.34 (1.13- 4.85) | | | | | | | | 0.022 | | | | | | | |  |  |  |  |  |  |  |  |  |  |  |  |  |  |  |  |  |  |  |  |  |  |  |  |  |  |  |  |  |  |  |  |  |  |  |  |  |  |  |  |  |  |  |  |
|  |  |  |  |  |  |  |  |  |  |  |  |  |  |  |  |  |  |  |  |  |  |  |  | Inverse variance weighted | | | | | | | 0.54 | | | | | | | | 0.26 | | | | | | | | 1.72 (1.02- 2.92) | | | | | | | | 0.043 | | | | | | | |  |  |  |  |  |  |  |  |  |  |  |  |  |  |  |  |  |  |  |  |  |  |  |  |  |  |  |  |  |  |  |  |  |  |  |  |  |  |  |  |  |  |  |  |
|  |  |  |  |  |  |  |  |  |  |  |  |  |  |  |  |  |  |  |  |  |  |  |  | Simple mode | | | | | | | 1.17 | | | | | | | | 0.65 | | | | | | | | 3.24 (0.90-11.60) | | | | | | | | 0.090 | | | | | | | |  |  |  |  |  |  |  |  |  |  |  |  |  |  |  |  |  |  |  |  |  |  |  |  |  |  |  |  |  |  |  |  |  |  |  |  |  |  |  |  |  |  |  |  |
|  |  |  |  |  |  |  |  |  |  |  |  |  |  |  |  |  |  |  |  |  |  |  |  | Weighted mode | | | | | | | 1.10 | | | | | | | | 0.56 | | | | | | | | 3.03 (1.00- 9.20) | | | | | | | | 0.068 | | | | | | | |  |  |  |  |  |  |  |  |  |  |  |  |  |  |  |  |  |  |  |  |  |  |  |  |  |  |  |  |  |  |  |  |  |  |  |  |  |  |  |  |  |  |  |  |
| genus | | | Lachnospiraceae UCG001 | | | | | | | Acohol-induced acute pancreatitis | | | | | | | | 13 | | | | | | MR-Egger | | | | | | | -0.45 | | | | | | | | 1.15 | | | | | | | | 0.64 (0.07-6.13) | | | | | | | | 0.703 | | | | | | | | 0.95 | | | | | | | | 5.136 | | | | | | | | 0.953 | | | | | | 0.1076 | | | | | | | | 0.105 | | | | | | | | 0.328 | | | | | |
|  |  |  |  |  |  |  |  |  |  |  |  |  |  |  |  |  |  |  |  |  |  |  |  | Weighted median | | | | | | | 0.59 | | | | | | | | 0.34 | | | | | | | | 1.82 (0.93-3.56) | | | | | | | | 0.078 | | | | | | | |  |  |  |  |  |  |  |  |  |  |  |  |  |  |  |  |  |  |  |  |  |  |  |  |  |  |  |  |  |  |  |  |  |  |  |  |  |  |  |  |  |  |  |  |
|  |  |  |  |  |  |  |  |  |  |  |  |  |  |  |  |  |  |  |  |  |  |  |  | Inverse variance weighted | | | | | | | 0.69 | | | | | | | | 0.26 | | | | | | | | 2.01 (1.19-3.39) | | | | | | | | 0.008 | | | | | | | |  |  |  |  |  |  |  |  |  |  |  |  |  |  |  |  |  |  |  |  |  |  |  |  |  |  |  |  |  |  |  |  |  |  |  |  |  |  |  |  |  |  |  |  |
|  |  |  |  |  |  |  |  |  |  |  |  |  |  |  |  |  |  |  |  |  |  |  |  | Simple mode | | | | | | | 0.58 | | | | | | | | 0.51 | | | | | | | | 1.79 (0.66-4.89) | | | | | | | | 0.276 | | | | | | | |  |  |  |  |  |  |  |  |  |  |  |  |  |  |  |  |  |  |  |  |  |  |  |  |  |  |  |  |  |  |  |  |  |  |  |  |  |  |  |  |  |  |  |  |
|  |  |  |  |  |  |  |  |  |  |  |  |  |  |  |  |  |  |  |  |  |  |  |  | Weighted mode | | | | | | | 0.58 | | | | | | | | 0.46 | | | | | | | | 1.79 (0.72-4.47) | | | | | | | | 0.234 | | | | | | | |  |  |  |  |  |  |  |  |  |  |  |  |  |  |  |  |  |  |  |  |  |  |  |  |  |  |  |  |  |  |  |  |  |  |  |  |  |  |  |  |  |  |  |  |
| genus | | | Sellimonas | | | | | | | Acohol-induced acute pancreatitis | | | | | | | | 9 | | | | | | MR-Egger | | | | | | | -1.23 | | | | | | | | 1.07 | | | | | | | | 0.29 (0.04-2.37) | | | | | | | | 0.285 | | | | | | | | 0.76 | | | | | | | | 6.268 | | | | | | | | 0.6171 | | | | | | 0.256 | | | | | | | | 0.154 | | | | | | | | 0.140 | | | | | |
|  |  |  |  |  |  |  |  |  |  |  |  |  |  |  |  |  |  |  |  |  |  |  |  | Weighted median | | | | | | | 0.71 | | | | | | | | 0.25 | | | | | | | | 2.04 (1.24-3.37) | | | | | | | | 0.005 | | | | | | | |  |  |  |  |  |  |  |  |  |  |  |  |  |  |  |  |  |  |  |  |  |  |  |  |  |  |  |  |  |  |  |  |  |  |  |  |  |  |  |  |  |  |  |  |
|  |  |  |  |  |  |  |  |  |  |  |  |  |  |  |  |  |  |  |  |  |  |  |  | Inverse variance weighted | | | | | | | 0.51 | | | | | | | | 0.18 | | | | | | | | 1.67 (1.17-2.40) | | | | | | | | 0.004 | | | | | | | |  |  |  |  |  |  |  |  |  |  |  |  |  |  |  |  |  |  |  |  |  |  |  |  |  |  |  |  |  |  |  |  |  |  |  |  |  |  |  |  |  |  |  |  |
|  |  |  |  |  |  |  |  |  |  |  |  |  |  |  |  |  |  |  |  |  |  |  |  | Simple mode | | | | | | | 0.74 | | | | | | | | 0.35 | | | | | | | | 2.12 (1.05-4.25) | | | | | | | | 0.068 | | | | | | | |  |  |  |  |  |  |  |  |  |  |  |  |  |  |  |  |  |  |  |  |  |  |  |  |  |  |  |  |  |  |  |  |  |  |  |  |  |  |  |  |  |  |  |  |
|  |  |  |  |  |  |  |  |  |  |  |  |  |  |  |  |  |  |  |  |  |  |  |  | Weighted mode | | | | | | | 0.74 | | | | | | | | 0.39 | | | | | | | | 2.12 (0.98-4.56) | | | | | | | | 0.092 | | | | | | | |  |  |  |  |  |  |  |  |  |  |  |  |  |  |  |  |  |  |  |  |  |  |  |  |  |  |  |  |  |  |  |  |  |  |  |  |  |  |  |  |  |  |  |  |
| **Supplementary Table 6**: In reverse MR results and sensitivity analysis of AAP on gut microbiota. | | | | | | | | | | | | | | | | | | | | | | | | | | | | | | | | | | | | | | | | | | | | | | | | | | | | | | | | | | | | | | | | | | | | | | | | | | | | | | | | | | | | | | | | | | | | | | | | | | | | | | | | | | |
| **Taxa** | | | | **exposure** | | | | | | | **outcome** | | | | | | | | **Nsnp** | | | | | | **Methods** | | | | | | | **Beta** | | | | | | | | **SE** | | | | | | | | **OR (95%CI)** | | | | | | | | ***P* value** | | | | | | | | **MR-PRESSO** | | | | | | | | **Heterogeneity** | | | | | | | | | | | | | | **Horizontal pleiotrop** | | | | | | | | | | | | | | | | | | | | |
|  |  |  |  |  |  |  |  |  |  |  |  |  |  |  |  |  |  |  |  |  |  |  |  |  |  |  |  |  |  |  |  |  |  |  |  |  |  |  |  |  |  |  |  |  |  |  |  |  |  |  |  |  |  |  |  |  |  |  |  |  |  |  |  |  |  |  |  |  |  |  |  | **Cochran’s Q** | | | | | | | | ***P* value** | | | | | | **Egger intercept** | | | | | | | | **SE** | | | | | | | | ***P* value** | | | | |
| phylum | | | | Acohol-induced acute pancreatitis | | | | | | | Tenericutes | | | | | | | | 6 | | | | | | MR-Egger | | | | | | | -0.01 | | | | | | | | 0.05 | | | | | | | | 0.98 (0.88-1.10) | | | | | | | | 0.794 | | | | | | | | 0.55 | | | | | | | | 4.019 | | | | | | | | 0.546 | | | | | | -0.007 | | | | | | | | 0.0218 | | | | | | | | 0.747 | | | | |
|  |  |  |  |  |  |  |  |  |  |  |  |  |  |  |  |  |  |  |  |  |  |  |  |  | Weighted median | | | | | | | -0.04 | | | | | | | | 0.02 | | | | | | | | 0.95 (0.91-0.99) | | | | | | | | 0.022 | | | | | | | |  |  |  |  |  |  |  |  |  |  |  |  |  |  |  |  |  |  |  |  |  |  |  |  |  |  |  |  |  |  |  |  |  |  |  |  |  |  |  |  |  |  |  |
|  |  |  |  |  |  |  |  |  |  |  |  |  |  |  |  |  |  |  |  |  |  |  |  |  | Inverse variance weighted | | | | | | | -0.03 | | | | | | | | 0.01 | | | | | | | | 0.97 (0.94-0.99) | | | | | | | | 0.032 | | | | | | | |  |  |  |  |  |  |  |  |  |  |  |  |  |  |  |  |  |  |  |  |  |  |  |  |  |  |  |  |  |  |  |  |  |  |  |  |  |  |  |  |  |  |  |
|  |  |  |  |  |  |  |  |  |  |  |  |  |  |  |  |  |  |  |  |  |  |  |  |  | Simple mode | | | | | | | -0.05 | | | | | | | | 0.03 | | | | | | | | 0.94 (0.88-1.01) | | | | | | | | 0.173 | | | | | | | |  |  |  |  |  |  |  |  |  |  |  |  |  |  |  |  |  |  |  |  |  |  |  |  |  |  |  |  |  |  |  |  |  |  |  |  |  |  |  |  |  |  |  |
|  |  |  |  |  |  |  |  |  |  |  |  |  |  |  |  |  |  |  |  |  |  |  |  |  | Weighted mode | | | | | | | -0.05 | | | | | | | | 0.03 | | | | | | | | 0.95 (0.89-1.01) | | | | | | | | 0.133 | | | | | | | |  |  |  |  |  |  |  |  |  |  |  |  |  |  |  |  |  |  |  |  |  |  |  |  |  |  |  |  |  |  |  |  |  |  |  |  |  |  |  |  |  |  |  |
| phylum | | | | Acohol-induced acute pancreatitis | | | | | | | Firmicutes | | | | | | | | 6 | | | | | | MR-Egger | | | | | | | -0.03 | | | | | | | | 0.04 | | | | | | | | 0.97 (0.89-1.06) | | | | | | | | 0.535 | | | | | | | | 0.78 | | | | | | | | 3.069 | | | | | | | | 0.689 | | | | | | 0.001 | | | | | | | | 0.0172 | | | | | | | | 0.952 | | | | |
|  |  |  |  |  |  |  |  |  |  |  |  |  |  |  |  |  |  |  |  |  |  |  |  |  | Weighted median | | | | | | | -0.02 | | | | | | | | 0.01 | | | | | | | | 0.98 (0.94-1.01) | | | | | | | | 0.156 | | | | | | | |  |  |  |  |  |  |  |  |  |  |  |  |  |  |  |  |  |  |  |  |  |  |  |  |  |  |  |  |  |  |  |  |  |  |  |  |  |  |  |  |  |  |  |
|  |  |  |  |  |  |  |  |  |  |  |  |  |  |  |  |  |  |  |  |  |  |  |  |  | Inverse variance weighted | | | | | | | -0.02 | | | | | | | | 0.01 | | | | | | | | 0.97 (0.95-0.99) | | | | | | | | 0.031 | | | | | | | |  |  |  |  |  |  |  |  |  |  |  |  |  |  |  |  |  |  |  |  |  |  |  |  |  |  |  |  |  |  |  |  |  |  |  |  |  |  |  |  |  |  |  |
|  |  |  |  |  |  |  |  |  |  |  |  |  |  |  |  |  |  |  |  |  |  |  |  |  | Simple mode | | | | | | | -0.02 | | | | | | | | 0.02 | | | | | | | | 0.98 (0.93-1.02) | | | | | | | | 0.374 | | | | | | | |  |  |  |  |  |  |  |  |  |  |  |  |  |  |  |  |  |  |  |  |  |  |  |  |  |  |  |  |  |  |  |  |  |  |  |  |  |  |  |  |  |  |  |
|  |  |  |  |  |  |  |  |  |  |  |  |  |  |  |  |  |  |  |  |  |  |  |  |  | Weighted mode | | | | | | | -0.02 | | | | | | | | 0.02 | | | | | | | | 0.98 (0.94-1.02) | | | | | | | | 0.332 | | | | | | | |  |  |  |  |  |  |  |  |  |  |  |  |  |  |  |  |  |  |  |  |  |  |  |  |  |  |  |  |  |  |  |  |  |  |  |  |  |  |  |  |  |  |  |
| classes | | | | Acohol-induced acute pancreatitis | | | | | | | Clostridia | | | | | | | | 6 | | | | | | MR-Egger | | | | | | | -0.001 | | | | | | | | 0.04 | | | | | | | | 1.00 (0.91-1.09) | | | | | | | | 0.967 | | | | | | | | 0.73 | | | | | | | | 3.108 | | | | | | | | 0.683 | | | | | | -0.009 | | | | | | | | 0.017 | | | | | | | | 0.5943 | | | | |
|  |  |  |  |  |  |  |  |  |  |  |  |  |  |  |  |  |  |  |  |  |  |  |  |  | Weighted median | | | | | | | -0.02 | | | | | | | | 0.01 | | | | | | | | 0.98 (0.95-1.01) | | | | | | | | 0.183 | | | | | | | |  |  |  |  |  |  |  |  |  |  |  |  |  |  |  |  |  |  |  |  |  |  |  |  |  |  |  |  |  |  |  |  |  |  |  |  |  |  |  |  |  |  |  |
|  |  |  |  |  |  |  |  |  |  |  |  |  |  |  |  |  |  |  |  |  |  |  |  |  | Inverse variance weighted | | | | | | | -0.02 | | | | | | | | 0.01 | | | | | | | | 0.97 (0.95-0.99) | | | | | | | | 0.036 | | | | | | | |  |  |  |  |  |  |  |  |  |  |  |  |  |  |  |  |  |  |  |  |  |  |  |  |  |  |  |  |  |  |  |  |  |  |  |  |  |  |  |  |  |  |  |
|  |  |  |  |  |  |  |  |  |  |  |  |  |  |  |  |  |  |  |  |  |  |  |  |  | Simple mode | | | | | | | -0.008 | | | | | | | | 0.02 | | | | | | | | 0.99 (0.95-1.04) | | | | | | | | 0.739 | | | | | | | |  |  |  |  |  |  |  |  |  |  |  |  |  |  |  |  |  |  |  |  |  |  |  |  |  |  |  |  |  |  |  |  |  |  |  |  |  |  |  |  |  |  |  |
|  |  |  |  |  |  |  |  |  |  |  |  |  |  |  |  |  |  |  |  |  |  |  |  |  | Weighted mode | | | | | | | -0.009 | | | | | | | | 0.02 | | | | | | | | 0.99 (0.95-1.03) | | | | | | | | 0.676 | | | | | | | |  |  |  |  |  |  |  |  |  |  |  |  |  |  |  |  |  |  |  |  |  |  |  |  |  |  |  |  |  |  |  |  |  |  |  |  |  |  |  |  |  |  |  |
| classes | | | | Acohol-induced acute pancreatitis | | | | | | | Coriobacteriia | | | | | | | | 6 | | | | | | MR-Egger | | | | | | | -0.02 | | | | | | | | 0.04 | | | | | | | | 1.03 (0.83-1.27) | | | | | | | | 0.646 | | | | | | | | 0.51 | | | | | | | | 4.672 | | | | | | | | 0.457 | | | | | | -0.0005 | | | | | | | | 0.018 | | | | | | | | 0.976 | | | | |
|  |  |  |  |  |  |  |  |  |  |  |  |  |  |  |  |  |  |  |  |  |  |  |  |  | Weighted median | | | | | | | -0.01 | | | | | | | | 0.01 | | | | | | | | 0.95 (0.91-1.00) | | | | | | | | 0.462 | | | | | | | |  |  |  |  |  |  |  |  |  |  |  |  |  |  |  |  |  |  |  |  |  |  |  |  |  |  |  |  |  |  |  |  |  |  |  |  |  |  |  |  |  |  |  |
|  |  |  |  |  |  |  |  |  |  |  |  |  |  |  |  |  |  |  |  |  |  |  |  |  | Inverse variance weighted | | | | | | | -0.02 | | | | | | | | 0.01 | | | | | | | | 0.95 (0.92-0.99) | | | | | | | | 0.046 | | | | | | | |  |  |  |  |  |  |  |  |  |  |  |  |  |  |  |  |  |  |  |  |  |  |  |  |  |  |  |  |  |  |  |  |  |  |  |  |  |  |  |  |  |  |  |
|  |  |  |  |  |  |  |  |  |  |  |  |  |  |  |  |  |  |  |  |  |  |  |  |  | Simple mode | | | | | | | -0.008 | | | | | | | | 0.02 | | | | | | | | 0.94 (0.86-1.03) | | | | | | | | 0.751 | | | | | | | |  |  |  |  |  |  |  |  |  |  |  |  |  |  |  |  |  |  |  |  |  |  |  |  |  |  |  |  |  |  |  |  |  |  |  |  |  |  |  |  |  |  |  |
|  |  |  |  |  |  |  |  |  |  |  |  |  |  |  |  |  |  |  |  |  |  |  |  |  | Weighted mode | | | | | | | -0.01 | | | | | | | | 0.02 | | | | | | | | 0.93 (0.85-1.02) | | | | | | | | 0.695 | | | | | | | |  |  |  |  |  |  |  |  |  |  |  |  |  |  |  |  |  |  |  |  |  |  |  |  |  |  |  |  |  |  |  |  |  |  |  |  |  |  |  |  |  |  |  |
| classes | | | | Acohol-induced acute pancreatitis | | | | | | | Mollicutes | | | | | | | | 6 | | | | | | MR-Egger | | | | | | | -0.01 | | | | | | | | 0.05 | | | | | | | | 0.98 (0.88-1.10) | | | | | | | | 0.794 | | | | | | | | 0.67 | | | | | | | | 4.019 | | | | | | | | 0.546 | | | | | | -0.0075 | | | | | | | | 0.021 | | | | | | | | 0.747 | | | | |
|  |  |  |  |  |  |  |  |  |  |  |  |  |  |  |  |  |  |  |  |  |  |  |  |  | Weighted median | | | | | | | -0.04 | | | | | | | | 0.02 | | | | | | | | 0.95 (0.91-0.99) | | | | | | | | 0.024 | | | | | | | |  |  |  |  |  |  |  |  |  |  |  |  |  |  |  |  |  |  |  |  |  |  |  |  |  |  |  |  |  |  |  |  |  |  |  |  |  |  |  |  |  |  |  |
|  |  |  |  |  |  |  |  |  |  |  |  |  |  |  |  |  |  |  |  |  |  |  |  |  | Inverse variance weighted | | | | | | | -0.03 | | | | | | | | 0.01 | | | | | | | | 0.97 (0.94-0.99) | | | | | | | | 0.032 | | | | | | | |  |  |  |  |  |  |  |  |  |  |  |  |  |  |  |  |  |  |  |  |  |  |  |  |  |  |  |  |  |  |  |  |  |  |  |  |  |  |  |  |  |  |  |
|  |  |  |  |  |  |  |  |  |  |  |  |  |  |  |  |  |  |  |  |  |  |  |  |  | Simple mode | | | | | | | -0.05 | | | | | | | | 0.03 | | | | | | | | 0.94 (0.88-1.01) | | | | | | | | 0.155 | | | | | | | |  |  |  |  |  |  |  |  |  |  |  |  |  |  |  |  |  |  |  |  |  |  |  |  |  |  |  |  |  |  |  |  |  |  |  |  |  |  |  |  |  |  |  |
|  |  |  |  |  |  |  |  |  |  |  |  |  |  |  |  |  |  |  |  |  |  |  |  |  | Weighted mode | | | | | | | -0.05 | | | | | | | | 0.03 | | | | | | | | 0.95 (0.88-1.01) | | | | | | | | 0.156 | | | | | | | |  |  |  |  |  |  |  |  |  |  |  |  |  |  |  |  |  |  |  |  |  |  |  |  |  |  |  |  |  |  |  |  |  |  |  |  |  |  |  |  |  |  |  |
| orders | | | | Acohol-induced acute pancreatitis | | | | | | | Clostridiales | | | | | | | | 6 | | | | | | MR-Egger | | | | | | | -0.001 | | | | | | | | 0.04 | | | | | | | | 1.00 (0.91-1.09) | | | | | | | | 0.969 | | | | | | | | 0.65 | | | | | | | | 3.238 | | | | | | | | 0.663 | | | | | | -0.010 | | | | | | | | 0.0172 | | | | | | | | 0.5923 | | | | |
|  |  |  |  |  |  |  |  |  |  |  |  |  |  |  |  |  |  |  |  |  |  |  |  |  | Weighted median | | | | | | | -0.02 | | | | | | | | 0.01 | | | | | | | | 0.98 (0.95-1.01) | | | | | | | | 0.185 | | | | | | | |  |  |  |  |  |  |  |  |  |  |  |  |  |  |  |  |  |  |  |  |  |  |  |  |  |  |  |  |  |  |  |  |  |  |  |  |  |  |  |  |  |  |  |
|  |  |  |  |  |  |  |  |  |  |  |  |  |  |  |  |  |  |  |  |  |  |  |  |  | Inverse variance weighted | | | | | | | -0.02 | | | | | | | | 0.01 | | | | | | | | 0.97 (0.95-0.99) | | | | | | | | 0.036 | | | | | | | |  |  |  |  |  |  |  |  |  |  |  |  |  |  |  |  |  |  |  |  |  |  |  |  |  |  |  |  |  |  |  |  |  |  |  |  |  |  |  |  |  |  |  |
|  |  |  |  |  |  |  |  |  |  |  |  |  |  |  |  |  |  |  |  |  |  |  |  |  | Simple mode | | | | | | | -0.009 | | | | | | | | 0.02 | | | | | | | | 0.99 (0.94-1.04) | | | | | | | | 0.725 | | | | | | | |  |  |  |  |  |  |  |  |  |  |  |  |  |  |  |  |  |  |  |  |  |  |  |  |  |  |  |  |  |  |  |  |  |  |  |  |  |  |  |  |  |  |  |
|  |  |  |  |  |  |  |  |  |  |  |  |  |  |  |  |  |  |  |  |  |  |  |  |  | Weighted mode | | | | | | | -0.01 | | | | | | | | 0.02 | | | | | | | | 0.99 (0.95-1.04) | | | | | | | | 0.670 | | | | | | | |  |  |  |  |  |  |  |  |  |  |  |  |  |  |  |  |  |  |  |  |  |  |  |  |  |  |  |  |  |  |  |  |  |  |  |  |  |  |  |  |  |  |  |
| orders | | | | Acohol-induced acute pancreatitis | | | | | | | Coriobacteriales | | | | | | | | 6 | | | | | | MR-Egger | | | | | | | -0.02 | | | | | | | | 0.04 | | | | | | | | 0.98 (0.89-1.08) | | | | | | | | 0.646 | | | | | | | | 0.43 | | | | | | | | 4.672 | | | | | | | | 0.457 | | | | | | -0.00059 | | | | | | | | 0.0189 | | | | | | | | 0.976 | | | | |
|  |  |  |  |  |  |  |  |  |  |  |  |  |  |  |  |  |  |  |  |  |  |  |  |  | Weighted median | | | | | | | -0.01 | | | | | | | | 0.01 | | | | | | | | 0.99 (0.95-1.02) | | | | | | | | 0.460 | | | | | | | |  |  |  |  |  |  |  |  |  |  |  |  |  |  |  |  |  |  |  |  |  |  |  |  |  |  |  |  |  |  |  |  |  |  |  |  |  |  |  |  |  |  |  |
|  |  |  |  |  |  |  |  |  |  |  |  |  |  |  |  |  |  |  |  |  |  |  |  |  | Inverse variance weighted | | | | | | | -0.02 | | | | | | | | 0.01 | | | | | | | | 0.97  (0.95-0.99) | | | | | | | | 0.046 | | | | | | | |  |  |  |  |  |  |  |  |  |  |  |  |  |  |  |  |  |  |  |  |  |  |  |  |  |  |  |  |  |  |  |  |  |  |  |  |  |  |  |  |  |  |  |
|  |  |  |  |  |  |  |  |  |  |  |  |  |  |  |  |  |  |  |  |  |  |  |  |  | Simple mode | | | | | | | -0.008 | | | | | | | | 0.02 | | | | | | | | 0.99 (0.94-1.04) | | | | | | | | 0.741 | | | | | | | |  |  |  |  |  |  |  |  |  |  |  |  |  |  |  |  |  |  |  |  |  |  |  |  |  |  |  |  |  |  |  |  |  |  |  |  |  |  |  |  |  |  |  |
|  |  |  |  |  |  |  |  |  |  |  |  |  |  |  |  |  |  |  |  |  |  |  |  |  | Weighted mode | | | | | | | -0.01 | | | | | | | | 0.02 | | | | | | | | 0.99 (0.95-1.04) | | | | | | | | 0.680 | | | | | | | |  |  |  |  |  |  |  |  |  |  |  |  |  |  |  |  |  |  |  |  |  |  |  |  |  |  |  |  |  |  |  |  |  |  |  |  |  |  |  |  |  |  |  |
| orders | | | | Acohol-induced acute pancreatitis | | | | | | | Mollicutes RF9 | | | | | | | | 6 | | | | | | MR-Egger | | | | | | | -0.03 | | | | | | | | 0.06 | | | | | | | | 0.96 (0.85-1.08) | | | | | | | | 0.544 | | | | | | | | 0.82 | | | | | | | | 2.424 | | | | | | | | 0.787 | | | | | | 0.0005 | | | | | | | | 0.022 | | | | | | | | 0.983 | | | | |
|  |  |  |  |  |  |  |  |  |  |  |  |  |  |  |  |  |  |  |  |  |  |  |  |  | Weighted median | | | | | | | -0.04 | | | | | | | | 0.02 | | | | | | | | 0.96 (0.92-0.99) | | | | | | | | 0.026 | | | | | | | |  |  |  |  |  |  |  |  |  |  |  |  |  |  |  |  |  |  |  |  |  |  |  |  |  |  |  |  |  |  |  |  |  |  |  |  |  |  |  |  |  |  |  |
|  |  |  |  |  |  |  |  |  |  |  |  |  |  |  |  |  |  |  |  |  |  |  |  |  | Inverse variance weighted | | | | | | | -0.03 | | | | | | | | 0.01 | | | | | | | | 0.96 (0.93-1.00) | | | | | | | | 0.024 | | | | | | | |  |  |  |  |  |  |  |  |  |  |  |  |  |  |  |  |  |  |  |  |  |  |  |  |  |  |  |  |  |  |  |  |  |  |  |  |  |  |  |  |  |  |  |
|  |  |  |  |  |  |  |  |  |  |  |  |  |  |  |  |  |  |  |  |  |  |  |  |  | Simple mode | | | | | | | -0.05 | | | | | | | | 0.03 | | | | | | | | 0.95 (0.89-1.01) | | | | | | | | 0.183 | | | | | | | |  |  |  |  |  |  |  |  |  |  |  |  |  |  |  |  |  |  |  |  |  |  |  |  |  |  |  |  |  |  |  |  |  |  |  |  |  |  |  |  |  |  |  |
|  |  |  |  |  |  |  |  |  |  |  |  |  |  |  |  |  |  |  |  |  |  |  |  |  | Weighted mode | | | | | | | -0.05 | | | | | | | | 0.03 | | | | | | | | 0.95 (0.89-1.01) | | | | | | | | 0.158 | | | | | | | |  |  |  |  |  |  |  |  |  |  |  |  |  |  |  |  |  |  |  |  |  |  |  |  |  |  |  |  |  |  |  |  |  |  |  |  |  |  |  |  |  |  |  |
| families | | | | Acohol-induced acute pancreatitis | | | | | | | Coriobacteriaceae | | | | | | | | 6 | | | | | | MR-Egger | | | | | | | -0.02 | | | | | | | | 0.04 | | | | | | | | 0.98 (0.89-1.08) | | | | | | | | 0.646 | | | | | | | | 0.5 | | | | | | | | 4.672 | | | | | | | | 0.457 | | | | | | -0.00059 | | | | | | | | 0.0189 | | | | | | | | 0.976 | | | | |
|  |  |  |  |  |  |  |  |  |  |  |  |  |  |  |  |  |  |  |  |  |  |  |  |  | Weighted median | | | | | | | -0.01 | | | | | | | | 0.01 | | | | | | | | 0.99 (0.95-1.02) | | | | | | | | 0.455 | | | | | | | |  |  |  |  |  |  |  |  |  |  |  |  |  |  |  |  |  |  |  |  |  |  |  |  |  |  |  |  |  |  |  |  |  |  |  |  |  |  |  |  |  |  |  |
|  |  |  |  |  |  |  |  |  |  |  |  |  |  |  |  |  |  |  |  |  |  |  |  |  | Inverse variance weighted | | | | | | | -0.02 | | | | | | | | 0.01 | | | | | | | | 0.97 (0.95-0.99) | | | | | | | | 0.046 | | | | | | | |  |  |  |  |  |  |  |  |  |  |  |  |  |  |  |  |  |  |  |  |  |  |  |  |  |  |  |  |  |  |  |  |  |  |  |  |  |  |  |  |  |  |  |
|  |  |  |  |  |  |  |  |  |  |  |  |  |  |  |  |  |  |  |  |  |  |  |  |  | Simple mode | | | | | | | -0.008 | | | | | | | | 0.02 | | | | | | | | 0.99 (0.94-1.04) | | | | | | | | 0.742 | | | | | | | |  |  |  |  |  |  |  |  |  |  |  |  |  |  |  |  |  |  |  |  |  |  |  |  |  |  |  |  |  |  |  |  |  |  |  |  |  |  |  |  |  |  |  |
|  |  |  |  |  |  |  |  |  |  |  |  |  |  |  |  |  |  |  |  |  |  |  |  |  | Weighted mode | | | | | | | -0.010 | | | | | | | | 0.02 | | | | | | | | 0.99 (0.94-1.04) | | | | | | | | 0.697 | | | | | | | |  |  |  |  |  |  |  |  |  |  |  |  |  |  |  |  |  |  |  |  |  |  |  |  |  |  |  |  |  |  |  |  |  |  |  |  |  |  |  |  |  |  |  |
| families | | | | Acohol-induced acute pancreatitis | | | | | | | Rhodospirillaceae | | | | | | | | 6 | | | | | | MR-Egger | | | | | | | 0.01 | | | | | | | | 0.06 | | | | | | | | 1.00 (0.88-1.14) | | | | | | | | 0.988 | | | | | | | | 0.53 | | | | | | | | 4.061 | | | | | | | | 0.540 | | | | | | -0.0152 | | | | | | | | 0.0248 | | | | | | | | 0.573 | | | | |
|  |  |  |  |  |  |  |  |  |  |  |  |  |  |  |  |  |  |  |  |  |  |  |  |  | Weighted median | | | | | | | -0.02 | | | | | | | | 0.02 | | | | | | | | 0.98 (0.93-1.02) | | | | | | | | 0.305 | | | | | | | |  |  |  |  |  |  |  |  |  |  |  |  |  |  |  |  |  |  |  |  |  |  |  |  |  |  |  |  |  |  |  |  |  |  |  |  |  |  |  |  |  |  |  |
|  |  |  |  |  |  |  |  |  |  |  |  |  |  |  |  |  |  |  |  |  |  |  |  |  | Inverse variance weighted | | | | | | | -0.03 | | | | | | | | 0.01 | | | | | | | | 0.96 (0.93-1.00) | | | | | | | | 0.048 | | | | | | | |  |  |  |  |  |  |  |  |  |  |  |  |  |  |  |  |  |  |  |  |  |  |  |  |  |  |  |  |  |  |  |  |  |  |  |  |  |  |  |  |  |  |  |
|  |  |  |  |  |  |  |  |  |  |  |  |  |  |  |  |  |  |  |  |  |  |  |  |  | Simple mode | | | | | | | -0.01 | | | | | | | | 0.03 | | | | | | | | 0.98 (0.91-1.05) | | | | | | | | 0.606 | | | | | | | |  |  |  |  |  |  |  |  |  |  |  |  |  |  |  |  |  |  |  |  |  |  |  |  |  |  |  |  |  |  |  |  |  |  |  |  |  |  |  |  |  |  |  |
|  |  |  |  |  |  |  |  |  |  |  |  |  |  |  |  |  |  |  |  |  |  |  |  |  | Weighted mode | | | | | | | -0.01 | | | | | | | | 0.03 | | | | | | | | 0.98 (0.92-1.05) | | | | | | | | 0.607 | | | | | | | |  |  |  |  |  |  |  |  |  |  |  |  |  |  |  |  |  |  |  |  |  |  |  |  |  |  |  |  |  |  |  |  |  |  |  |  |  |  |  |  |  |  |  |
| genus | | | | Acohol-induced acute pancreatitis | | | | | | | Eubacterium xylanophilum group | | | | | | | | 6 | | | | | | MR-Egger | | | | | | | -0.02 | | | | | | | | 0.05 | | | | | | | | 0.98 (0.88-1.08) | | | | | | | | 0.690 | | | | | | | | 0.66 | | | | | | | | 3.9766 | | | | | | | | 0.552 | | | | | | -0.0066 | | | | | | | | 0.0200 | | | | | | | | 0.755 | | | | |
|  |  |  |  |  |  |  |  |  |  |  |  |  |  |  |  |  |  |  |  |  |  |  |  |  | Weighted median | | | | | | | -0.02 | | | | | | | | 0.01 | | | | | | | | 0.97 (0.94-1.01) | | | | | | | | 0.176 | | | | | | | |  |  |  |  |  |  |  |  |  |  |  |  |  |  |  |  |  |  |  |  |  |  |  |  |  |  |  |  |  |  |  |  |  |  |  |  |  |  |  |  |  |  |  |
|  |  |  |  |  |  |  |  |  |  |  |  |  |  |  |  |  |  |  |  |  |  |  |  |  | Inverse variance weighted | | | | | | | -0.03 | | | | | | | | 0.01 | | | | | | | | 0.96 (0.93-0.99) | | | | | | | | 0.009 | | | | | | | |  |  |  |  |  |  |  |  |  |  |  |  |  |  |  |  |  |  |  |  |  |  |  |  |  |  |  |  |  |  |  |  |  |  |  |  |  |  |  |  |  |  |  |
|  |  |  |  |  |  |  |  |  |  |  |  |  |  |  |  |  |  |  |  |  |  |  |  |  | Simple mode | | | | | | | -0.01 | | | | | | | | 0.03 | | | | | | | | 0.98 (0.93-1.04) | | | | | | | | 0.581 | | | | | | | |  |  |  |  |  |  |  |  |  |  |  |  |  |  |  |  |  |  |  |  |  |  |  |  |  |  |  |  |  |  |  |  |  |  |  |  |  |  |  |  |  |  |  |
|  |  |  |  |  |  |  |  |  |  |  |  |  |  |  |  |  |  |  |  |  |  |  |  |  | Weighted mode | | | | | | | -0.01 | | | | | | | | 0.02 | | | | | | | | 0.98 (0.93-1.04) | | | | | | | | 0.588 | | | | | | | |  |  |  |  |  |  |  |  |  |  |  |  |  |  |  |  |  |  |  |  |  |  |  |  |  |  |  |  |  |  |  |  |  |  |  |  |  |  |  |  |  |  |  |
| genus | | | | Acohol-induced acute pancreatitis | | | | | | | Oscillibacter | | | | | | | | 6 | | | | | | MR-Egger | | | | | | | 0.002 | | | | | | | | 0.06 | | | | | | | | 1.00 (0.88-1.14) | | | | | | | | 0.969 | | | | | | | | 0.8 | | | | | | | | 2.5439 | | | | | | | | 0.769 | | | | | | 0.01635 | | | | | | | | 0.024 | | | | | | | | 0.547 | | | | |
|  |  |  |  |  |  |  |  |  |  |  |  |  |  |  |  |  |  |  |  |  |  |  |  |  | Weighted median | | | | | | | 0.03 | | | | | | | | 0.02 | | | | | | | | 1.03 (0.99-1.08) | | | | | | | | 0.139 | | | | | | | |  |  |  |  |  |  |  |  |  |  |  |  |  |  |  |  |  |  |  |  |  |  |  |  |  |  |  |  |  |  |  |  |  |  |  |  |  |  |  |  |  |  |  |
|  |  |  |  |  |  |  |  |  |  |  |  |  |  |  |  |  |  |  |  |  |  |  |  |  | Inverse variance weighted | | | | | | | 0.04 | | | | | | | | 0.01 | | | | | | | | 1.05 (1.01-1.08) | | | | | | | | 0.017 | | | | | | | |  |  |  |  |  |  |  |  |  |  |  |  |  |  |  |  |  |  |  |  |  |  |  |  |  |  |  |  |  |  |  |  |  |  |  |  |  |  |  |  |  |  |  |
|  |  |  |  |  |  |  |  |  |  |  |  |  |  |  |  |  |  |  |  |  |  |  |  |  | Simple mode | | | | | | | 0.02 | | | | | | | | 0.03 | | | | | | | | 1.03 (0.96-1.10) | | | | | | | | 0.478 | | | | | | | |  |  |  |  |  |  |  |  |  |  |  |  |  |  |  |  |  |  |  |  |  |  |  |  |  |  |  |  |  |  |  |  |  |  |  |  |  |  |  |  |  |  |  |
|  |  |  |  |  |  |  |  |  |  |  |  |  |  |  |  |  |  |  |  |  |  |  |  |  | Weighted mode | | | | | | | 0.02 | | | | | | | | 0.03 | | | | | | | | 1.03 (0.97-1.09) | | | | | | | | 0.417 | | | | | | | |  |  |  |  |  |  |  |  |  |  |  |  |  |  |  |  |  |  |  |  |  |  |  |  |  |  |  |  |  |  |  |  |  |  |  |  |  |  |  |  |  |  |  |
| genus | | | | Acohol-induced acute pancreatitis | | | | | | | Ruminiclostridium6 | | | | | | | | 6 | | | | | | MR-Egger | | | | | | | -0.06 | | | | | | | | 0.05 | | | | | | | | 0.93 (0.84-1.04) | | | | | | | | 0.267 | | | | | | | | 0.68 | | | | | | | | 2.581 | | | | | | | | 0.764 | | | | | | 0.0145 | | | | | | | | 0.020 | | | | | | | | 0.5070 | | | | |
|  |  |  |  |  |  |  |  |  |  |  |  |  |  |  |  |  |  |  |  |  |  |  |  |  | Weighted median | | | | | | | -0.02 | | | | | | | | 0.01 | | | | | | | | 0.98 (0.94-1.02) | | | | | | | | 0.281 | | | | | | | |  |  |  |  |  |  |  |  |  |  |  |  |  |  |  |  |  |  |  |  |  |  |  |  |  |  |  |  |  |  |  |  |  |  |  |  |  |  |  |  |  |  |  |
|  |  |  |  |  |  |  |  |  |  |  |  |  |  |  |  |  |  |  |  |  |  |  |  |  | Inverse variance weighted | | | | | | | -0.03 | | | | | | | | 0.01 | | | | | | | | 0.97 (0.94-0.99) | | | | | | | | 0.039 | | | | | | | |  |  |  |  |  |  |  |  |  |  |  |  |  |  |  |  |  |  |  |  |  |  |  |  |  |  |  |  |  |  |  |  |  |  |  |  |  |  |  |  |  |  |  |
|  |  |  |  |  |  |  |  |  |  |  |  |  |  |  |  |  |  |  |  |  |  |  |  |  | Simple mode | | | | | | | -0.01 | | | | | | | | 0.02 | | | | | | | | 0.99 (0.93-1.04) | | | | | | | | 0.670 | | | | | | | |  |  |  |  |  |  |  |  |  |  |  |  |  |  |  |  |  |  |  |  |  |  |  |  |  |  |  |  |  |  |  |  |  |  |  |  |  |  |  |  |  |  |  |
|  |  |  |  |  |  |  |  |  |  |  |  |  |  |  |  |  |  |  |  |  |  |  |  |  | Weighted mode | | | | | | | -0.01 | | | | | | | | 0.02 | | | | | | | | 0.99 (0.93-1.05) | | | | | | | | 0.666 | | | | | | | |  |  |  |  |  |  |  |  |  |  |  |  |  |  |  |  |  |  |  |  |  |  |  |  |  |  |  |  |  |  |  |  |  |  |  |  |  |  |  |  |  |  |  |
| genus | | | | Acohol-induced acute pancreatitis | | | | | | | Ruminococcaceae UCG014 | | | | | | | | 6 | | | | | | MR-Egger | | | | | | | -0.05 | | | | | | | | 0.05 | | | | | | | | 0.95 (0.86-1.04) | | | | | | | | 0.330 | | | | | | | | 0.91 | | | | | | | | 1.786 | | | | | | | | 0.877 | | | | | | 0.0034 | | | | | | | | 0.0194 | | | | | | | | 0.865 | | | | |
|  |  |  |  |  |  |  |  |  |  |  |  |  |  |  |  |  |  |  |  |  |  |  |  |  | Weighted median | | | | | | | -0.05 | | | | | | | | 0.01 | | | | | | | | 0.94 (0.91-0.98) | | | | | | | | 0.001 | | | | | | | |  |  |  |  |  |  |  |  |  |  |  |  |  |  |  |  |  |  |  |  |  |  |  |  |  |  |  |  |  |  |  |  |  |  |  |  |  |  |  |  |  |  |  |
|  |  |  |  |  |  |  |  |  |  |  |  |  |  |  |  |  |  |  |  |  |  |  |  |  | Inverse variance weighted | | | | | | | -0.04 | | | | | | | | 0.01 | | | | | | | | 0.95 (0.93-0.98) | | | | | | | | 0.001 | | | | | | | |  |  |  |  |  |  |  |  |  |  |  |  |  |  |  |  |  |  |  |  |  |  |  |  |  |  |  |  |  |  |  |  |  |  |  |  |  |  |  |  |  |  |  |
|  |  |  |  |  |  |  |  |  |  |  |  |  |  |  |  |  |  |  |  |  |  |  |  |  | Simple mode | | | | | | | -0.06 | | | | | | | | 0.02 | | | | | | | | 0.94 (0.89-0.99) | | | | | | | | 0.080 | | | | | | | |  |  |  |  |  |  |  |  |  |  |  |  |  |  |  |  |  |  |  |  |  |  |  |  |  |  |  |  |  |  |  |  |  |  |  |  |  |  |  |  |  |  |  |
|  |  |  |  |  |  |  |  |  |  |  |  |  |  |  |  |  |  |  |  |  |  |  |  |  | Weighted mode | | | | | | | -0.06 | | | | | | | | 0.02 | | | | | | | | 0.94 (0.89-0.99) | | | | | | | | 0.084 | | | | | | | |  |  |  |  |  |  |  |  |  |  |  |  |  |  |  |  |  |  |  |  |  |  |  |  |  |  |  |  |  |  |  |  |  |  |  |  |  |  |  |  |  |  |  |
| **Supplementary Table 7:**MR results and sensitivity analysis of gut microbiota on ACP. | | | | | | | | | | | | | | | | | | | | | | | | | | | | | | | | | | | | | | | | | | | | | | | | | | | | | | | | | | | | | | | | | | | | | | | | | | | | | | | | | | | | | | | | | | | | | | | | | | | | | | | | | | |
| **Taxa** | | | **exposure** | | | | | | | | | | | | **outcome** | | | | | | **Nsnp** | | | | | | **Methods** | | | | | | | **Beta** | | | | | | | | **SE** | | | | | | | | **OR (95%CI)** | | | | | | | | ***P* value** | | | | | | | | **MR-PRESSO** | | | | | | | | **Heterogeneity** | | | | | | | | | | | | | **Horizontal pleiotrop** | | | | | | | | | | | | | | | | | | | |
|  |  |  |  |  |  |  |  |  |  |  |  |  |  |  |  |  |  |  |  |  |  |  |  |  |  |  |  |  |  |  |  |  |  |  |  |  |  |  |  |  |  |  |  |  |  |  |  |  |  |  |  |  |  |  |  |  |  |  |  |  |  |  |  |  |  |  |  |  |  |  |  |  |  | **Cochran’s Q** | | | | | | | ***P* value** | | | | | | **Egger intercept** | | | | | | | | **SE** | | | | | | | | ***P* value** | | | |
| classes | | | Gammaproteobacteria | | | | | | | | | | | | Alcohol-induced chronic pancreatitis | | | | | | 6 | | | | | | MR-Egger | | | | | | | 1.34 | | | | | | | | 1.12 | | | | | | | | 3.83 (0.42-34.50) | | | | | | | | 0.297 | | | | | | | | 0.66 | | | | | | | | 3.331 | | | | | | | 0.649 | | | | | | -0.043 | | | | | | | | 0.0819 | | | | | | | | 0.6236 | | | |
|  |  |  |  |  |  |  |  |  |  |  |  |  |  |  |  |  |  |  |  |  |  |  |  |  |  |  | Weighted median | | | | | | | 0.78 | | | | | | | | 0.49 | | | | | | | | 2.19 (0.83- 5.76) | | | | | | | | 0.112 | | | | | | | |  |  |  |  |  |  |  |  |  |  |  |  |  |  |  |  |  |  |  |  |  |  |  |  |  |  |  |  |  |  |  |  |  |  |  |  |  |  |  |  |  |
|  |  |  |  |  |  |  |  |  |  |  |  |  |  |  |  |  |  |  |  |  |  |  |  |  |  |  | Inverse variance weighted | | | | | | | 0.77 | | | | | | | | 0.36 | | | | | | | | 2.18 (1.06- 4.49) | | | | | | | | 0.034 | | | | | | | |  |  |  |  |  |  |  |  |  |  |  |  |  |  |  |  |  |  |  |  |  |  |  |  |  |  |  |  |  |  |  |  |  |  |  |  |  |  |  |  |  |
|  |  |  |  |  |  |  |  |  |  |  |  |  |  |  |  |  |  |  |  |  |  |  |  |  |  |  | Simple mode | | | | | | | 0.17 | | | | | | | | 0.74 | | | | | | | | 1.20 (0.28- 5.11) | | | | | | | | 0.817 | | | | | | | |  |  |  |  |  |  |  |  |  |  |  |  |  |  |  |  |  |  |  |  |  |  |  |  |  |  |  |  |  |  |  |  |  |  |  |  |  |  |  |  |  |
|  |  |  |  |  |  |  |  |  |  |  |  |  |  |  |  |  |  |  |  |  |  |  |  |  |  |  | Weighted mode | | | | | | | 1.06 | | | | | | | | 0.74 | | | | | | | | 2.89 (0.68-12.34) | | | | | | | | 0.211 | | | | | | | |  |  |  |  |  |  |  |  |  |  |  |  |  |  |  |  |  |  |  |  |  |  |  |  |  |  |  |  |  |  |  |  |  |  |  |  |  |  |  |  |  |
| classes | | | Melainabacteria | | | | | | | | | | | | Alcohol-induced chronic pancreatitis | | | | | | 10 | | | | | | MR-Egger | | | | | | | 1.07 | | | | | | | | 0.51 | | | | | | | | 2.92 (1.06-8.09) | | | | | | | | 0.072 | | | | | | | | 0.57 | | | | | | | | 7.271 | | | | | | | 0.608 | | | | | | -0.056 | | | | | | | | 0.0568 | | | | | | | | 0.3520 | | | |
|  |  |  |  |  |  |  |  |  |  |  |  |  |  |  |  |  |  |  |  |  |  |  |  |  |  |  | Weighted median | | | | | | | 0.63 | | | | | | | | 0.22 | | | | | | | | 1.90 (1.21-2.97) | | | | | | | | 0.005 | | | | | | | |  |  |  |  |  |  |  |  |  |  |  |  |  |  |  |  |  |  |  |  |  |  |  |  |  |  |  |  |  |  |  |  |  |  |  |  |  |  |  |  |  |
|  |  |  |  |  |  |  |  |  |  |  |  |  |  |  |  |  |  |  |  |  |  |  |  |  |  |  | Inverse variance weighted | | | | | | | 0.58 | | | | | | | | 0.17 | | | | | | | | 1.80 (1.29-2.52) | | | | | | | | 0.0005 | | | | | | | |  |  |  |  |  |  |  |  |  |  |  |  |  |  |  |  |  |  |  |  |  |  |  |  |  |  |  |  |  |  |  |  |  |  |  |  |  |  |  |  |  |
|  |  |  |  |  |  |  |  |  |  |  |  |  |  |  |  |  |  |  |  |  |  |  |  |  |  |  | Simple mode | | | | | | | 0.79 | | | | | | | | 0.37 | | | | | | | | 2.21 (1.07-4.59) | | | | | | | | 0.061 | | | | | | | |  |  |  |  |  |  |  |  |  |  |  |  |  |  |  |  |  |  |  |  |  |  |  |  |  |  |  |  |  |  |  |  |  |  |  |  |  |  |  |  |  |
|  |  |  |  |  |  |  |  |  |  |  |  |  |  |  |  |  |  |  |  |  |  |  |  |  |  |  | Weighted mode | | | | | | | 0.77 | | | | | | | | 0.33 | | | | | | | | 2.17 (1.13-4.15) | | | | | | | | 0.043 | | | | | | | |  |  |  |  |  |  |  |  |  |  |  |  |  |  |  |  |  |  |  |  |  |  |  |  |  |  |  |  |  |  |  |  |  |  |  |  |  |  |  |  |  |
| orders | | | Gastranaerophilales | | | | | | | | | | | | Alcohol-induced chronic pancreatitis | | | | | | 9 | | | | | | MR-Egger | | | | | | | 1.28 | | | | | | | | 0.54 | | | | | | | | 3.61 (1.24-10.44) | | | | | | | | 0.050 | | | | | | | | 0.38 | | | | | | | | 9.323 | | | | | | | 0.315 | | | | | | -0.089 | | | | | | | | 0.0617 | | | | | | | | 0.1888 | | | |
|  |  |  |  |  |  |  |  |  |  |  |  |  |  |  |  |  |  |  |  |  |  |  |  |  |  |  | Weighted median | | | | | | | 0.58 | | | | | | | | 0.25 | | | | | | | | 1.79 (1.10- 2.93) | | | | | | | | 0.019 | | | | | | | |  |  |  |  |  |  |  |  |  |  |  |  |  |  |  |  |  |  |  |  |  |  |  |  |  |  |  |  |  |  |  |  |  |  |  |  |  |  |  |  |  |
|  |  |  |  |  |  |  |  |  |  |  |  |  |  |  |  |  |  |  |  |  |  |  |  |  |  |  | Inverse variance weighted | | | | | | | 0.53 | | | | | | | | 0.19 | | | | | | | | 1.71 (1.17- 2.50) | | | | | | | | 0.005 | | | | | | | |  |  |  |  |  |  |  |  |  |  |  |  |  |  |  |  |  |  |  |  |  |  |  |  |  |  |  |  |  |  |  |  |  |  |  |  |  |  |  |  |  |
|  |  |  |  |  |  |  |  |  |  |  |  |  |  |  |  |  |  |  |  |  |  |  |  |  |  |  | Simple mode | | | | | | | 0.83 | | | | | | | | 0.40 | | | | | | | | 2.31 (1.05- 5.06) | | | | | | | | 0.070 | | | | | | | |  |  |  |  |  |  |  |  |  |  |  |  |  |  |  |  |  |  |  |  |  |  |  |  |  |  |  |  |  |  |  |  |  |  |  |  |  |  |  |  |  |
|  |  |  |  |  |  |  |  |  |  |  |  |  |  |  |  |  |  |  |  |  |  |  |  |  |  |  | Weighted mode | | | | | | | 0.78 | | | | | | | | 0.36 | | | | | | | | 2.19 (1.07- 4.49) | | | | | | | | 0.064 | | | | | | | |  |  |  |  |  |  |  |  |  |  |  |  |  |  |  |  |  |  |  |  |  |  |  |  |  |  |  |  |  |  |  |  |  |  |  |  |  |  |  |  |  |
| family | | | Clostridiaceae1 | | | | | | | | | | | | Alcohol-induced chronic pancreatitis | | | | | | 10 | | | | | | MR-Egger | | | | | | | -0.41 | | | | | | | | 0.74 | | | | | | | | 0.66 (0.15-2.82) | | | | | | | | 0.587 | | | | | | | | 0.56 | | | | | | | | 7.871 | | | | | | | 0.547 | | | | | | -0.0119 | | | | | | | | 0.0544 | | | | | | | | 0.8312 | | | |
|  |  |  |  |  |  |  |  |  |  |  |  |  |  |  |  |  |  |  |  |  |  |  |  |  |  |  | Weighted median | | | | | | | -0.54 | | | | | | | | 0.35 | | | | | | | | 0.58 (0.29-1.16) | | | | | | | | 0.122 | | | | | | | |  |  |  |  |  |  |  |  |  |  |  |  |  |  |  |  |  |  |  |  |  |  |  |  |  |  |  |  |  |  |  |  |  |  |  |  |  |  |  |  |  |
|  |  |  |  |  |  |  |  |  |  |  |  |  |  |  |  |  |  |  |  |  |  |  |  |  |  |  | Inverse variance weighted | | | | | | | -0.57 | | | | | | | | 0.25 | | | | | | | | 0.56 (0.34-0.93) | | | | | | | | 0.024 | | | | | | | |  |  |  |  |  |  |  |  |  |  |  |  |  |  |  |  |  |  |  |  |  |  |  |  |  |  |  |  |  |  |  |  |  |  |  |  |  |  |  |  |  |
|  |  |  |  |  |  |  |  |  |  |  |  |  |  |  |  |  |  |  |  |  |  |  |  |  |  |  | Simple mode | | | | | | | -0.58 | | | | | | | | 0.56 | | | | | | | | 0.56 (0.18-1.68) | | | | | | | | 0.326 | | | | | | | |  |  |  |  |  |  |  |  |  |  |  |  |  |  |  |  |  |  |  |  |  |  |  |  |  |  |  |  |  |  |  |  |  |  |  |  |  |  |  |  |  |
|  |  |  |  |  |  |  |  |  |  |  |  |  |  |  |  |  |  |  |  |  |  |  |  |  |  |  | Weighted mode | | | | | | | -0.57 | | | | | | | | 0.47 | | | | | | | | 0.56 (0.22-1.42) | | | | | | | | 0.256 | | | | | | | |  |  |  |  |  |  |  |  |  |  |  |  |  |  |  |  |  |  |  |  |  |  |  |  |  |  |  |  |  |  |  |  |  |  |  |  |  |  |  |  |  |
| genus | | | Butyricimonas | | | | | | | | | | | | Alcohol-induced chronic pancreatitis | | | | | | 13 | | | | | | MR-Egger | | | | | | | 0.85 | | | | | | | | 0.85 | | | | | | | | 2.36 (0.44-12.64) | | | | | | | | 0.337 | | | | | | | | 0.4 | | | | | | | | 14.00 | | | | | | | 0.300 | | | | | | -0.0356 | | | | | | | | 0.0730 | | | | | | | | 0.634 | | | |
|  |  |  |  |  |  |  |  |  |  |  |  |  |  |  |  |  |  |  |  |  |  |  |  |  |  |  | Weighted median | | | | | | | 0.41 | | | | | | | | 0.30 | | | | | | | | 1.51 (0.83- 2.75) | | | | | | | | 0.175 | | | | | | | |  |  |  |  |  |  |  |  |  |  |  |  |  |  |  |  |  |  |  |  |  |  |  |  |  |  |  |  |  |  |  |  |  |  |  |  |  |  |  |  |  |
|  |  |  |  |  |  |  |  |  |  |  |  |  |  |  |  |  |  |  |  |  |  |  |  |  |  |  | Inverse variance weighted | | | | | | | 0.45 | | | | | | | | 0.23 | | | | | | | | 1.58 (1.01- 2.49) | | | | | | | | 0.049 | | | | | | | |  |  |  |  |  |  |  |  |  |  |  |  |  |  |  |  |  |  |  |  |  |  |  |  |  |  |  |  |  |  |  |  |  |  |  |  |  |  |  |  |  |
|  |  |  |  |  |  |  |  |  |  |  |  |  |  |  |  |  |  |  |  |  |  |  |  |  |  |  | Simple mode | | | | | | | 0.43 | | | | | | | | 0.55 | | | | | | | | 1.55 (0.52- 4.61) | | | | | | | | 0.447 | | | | | | | |  |  |  |  |  |  |  |  |  |  |  |  |  |  |  |  |  |  |  |  |  |  |  |  |  |  |  |  |  |  |  |  |  |  |  |  |  |  |  |  |  |
|  |  |  |  |  |  |  |  |  |  |  |  |  |  |  |  |  |  |  |  |  |  |  |  |  |  |  | Weighted mode | | | | | | | 0.30 | | | | | | | | 0.54 | | | | | | | | 1.36 (0.47- 3.95) | | | | | | | | 0.581 | | | | | | | |  |  |  |  |  |  |  |  |  |  |  |  |  |  |  |  |  |  |  |  |  |  |  |  |  |  |  |  |  |  |  |  |  |  |  |  |  |  |  |  |  |
| genus | | | Enterorhabdus | | | | | | | | | | | | Alcohol-induced chronic pancreatitis | | | | | | 6 | | | | | | MR-Egger | | | | | | | 1.05 | | | | | | | | 0.67 | | | | | | | | 2.86 (0.76-10.70) | | | | | | | | 0.193 | | | | | | | | 0.64 | | | | | | | | 3.662 | | | | | | | 0.598 | | | | | | -0.067 | | | | | | | | 0.0868 | | | | | | | | 0.4817 | | | |
|  |  |  |  |  |  |  |  |  |  |  |  |  |  |  |  |  |  |  |  |  |  |  |  |  |  |  | Weighted median | | | | | | | 0.48 | | | | | | | | 0.34 | | | | | | | | 1.62 (0.83- 3.20) | | | | | | | | 0.159 | | | | | | | |  |  |  |  |  |  |  |  |  |  |  |  |  |  |  |  |  |  |  |  |  |  |  |  |  |  |  |  |  |  |  |  |  |  |  |  |  |  |  |  |  |
|  |  |  |  |  |  |  |  |  |  |  |  |  |  |  |  |  |  |  |  |  |  |  |  |  |  |  | Inverse variance weighted | | | | | | | 0.56 | | | | | | | | 0.25 | | | | | | | | 1.76 (1.07- 2.90) | | | | | | | | 0.025 | | | | | | | |  |  |  |  |  |  |  |  |  |  |  |  |  |  |  |  |  |  |  |  |  |  |  |  |  |  |  |  |  |  |  |  |  |  |  |  |  |  |  |  |  |
|  |  |  |  |  |  |  |  |  |  |  |  |  |  |  |  |  |  |  |  |  |  |  |  |  |  |  | Simple mode | | | | | | | 0.47 | | | | | | | | 0.47 | | | | | | | | 1.61 (0.63- 4.12) | | | | | | | | 0.362 | | | | | | | |  |  |  |  |  |  |  |  |  |  |  |  |  |  |  |  |  |  |  |  |  |  |  |  |  |  |  |  |  |  |  |  |  |  |  |  |  |  |  |  |  |
|  |  |  |  |  |  |  |  |  |  |  |  |  |  |  |  |  |  |  |  |  |  |  |  |  |  |  | Weighted mode | | | | | | | 0.51 | | | | | | | | 0.42 | | | | | | | | 1.68 (0.72- 3.89) | | | | | | | | 0.282 | | | | | | | |  |  |  |  |  |  |  |  |  |  |  |  |  |  |  |  |  |  |  |  |  |  |  |  |  |  |  |  |  |  |  |  |  |  |  |  |  |  |  |  |  |
| genus | | | Eubacterium oxidoreducens group | | | | | | | | | | | | Alcohol-induced chronic pancreatitis | | | | | | 5 | | | | | | MR-Egger | | | | | | | 1.32 | | | | | | | | 0.85 | | | | | | | | 3.75 (0.70-20.14) | | | | | | | | 0.220 | | | | | | | | 0.68 | | | | | | | | 2.6103 | | | | | | | 0.624 | | | | | | -0.0947 | | | | | | | | 0.0920 | | | | | | | | 0.379 | | | |
|  |  |  |  |  |  |  |  |  |  |  |  |  |  |  |  |  |  |  |  |  |  |  |  |  |  |  | Weighted median | | | | | | | 0.44 | | | | | | | | 0.28 | | | | | | | | 1.56 (0.89- 2.75) | | | | | | | | 0.121 | | | | | | | |  |  |  |  |  |  |  |  |  |  |  |  |  |  |  |  |  |  |  |  |  |  |  |  |  |  |  |  |  |  |  |  |  |  |  |  |  |  |  |  |  |
|  |  |  |  |  |  |  |  |  |  |  |  |  |  |  |  |  |  |  |  |  |  |  |  |  |  |  | Inverse variance weighted | | | | | | | 0.47 | | | | | | | | 0.22 | | | | | | | | 1.60 (1.02- 2.51) | | | | | | | | 0.039 | | | | | | | |  |  |  |  |  |  |  |  |  |  |  |  |  |  |  |  |  |  |  |  |  |  |  |  |  |  |  |  |  |  |  |  |  |  |  |  |  |  |  |  |  |
|  |  |  |  |  |  |  |  |  |  |  |  |  |  |  |  |  |  |  |  |  |  |  |  |  |  |  | Simple mode | | | | | | | 0.52 | | | | | | | | 0.38 | | | | | | | | 1.69 (0.80- 3.60) | | | | | | | | 0.242 | | | | | | | |  |  |  |  |  |  |  |  |  |  |  |  |  |  |  |  |  |  |  |  |  |  |  |  |  |  |  |  |  |  |  |  |  |  |  |  |  |  |  |  |  |
|  |  |  |  |  |  |  |  |  |  |  |  |  |  |  |  |  |  |  |  |  |  |  |  |  |  |  | Weighted mode | | | | | | | 0.51 | | | | | | | | 0.37 | | | | | | | | 1.67 (0.79- 3.51) | | | | | | | | 0.248 | | | | | | | |  |  |  |  |  |  |  |  |  |  |  |  |  |  |  |  |  |  |  |  |  |  |  |  |  |  |  |  |  |  |  |  |  |  |  |  |  |  |  |  |  |
| genus | | | Eubacterium xylanophilum group | | | | | | | | | | | | Alcohol-induced chronic pancreatitis | | | | | | 9 | | | | | | MR-Egger | | | | | | | 0.60 | | | | | | | | 0.72 | | | | | | | | 1.84 (0.44-7.59) | | | | | | | | 0.429 | | | | | | | | 0.64 | | | | | | | | 6.565 | | | | | | | 0.584 | | | | | | 0.002 | | | | | | | | 0.0593 | | | | | | | | 0.9611 | | | |
|  |  |  |  |  |  |  |  |  |  |  |  |  |  |  |  |  |  |  |  |  |  |  |  |  |  |  | Weighted median | | | | | | | 0.81 | | | | | | | | 0.32 | | | | | | | | 2.25 (1.19-4.25) | | | | | | | | 0.012 | | | | | | | |  |  |  |  |  |  |  |  |  |  |  |  |  |  |  |  |  |  |  |  |  |  |  |  |  |  |  |  |  |  |  |  |  |  |  |  |  |  |  |  |  |
|  |  |  |  |  |  |  |  |  |  |  |  |  |  |  |  |  |  |  |  |  |  |  |  |  |  |  | Inverse variance weighted | | | | | | | 0.64 | | | | | | | | 0.24 | | | | | | | | 1.90 (1.18-3.06) | | | | | | | | 0.008 | | | | | | | |  |  |  |  |  |  |  |  |  |  |  |  |  |  |  |  |  |  |  |  |  |  |  |  |  |  |  |  |  |  |  |  |  |  |  |  |  |  |  |  |  |
|  |  |  |  |  |  |  |  |  |  |  |  |  |  |  |  |  |  |  |  |  |  |  |  |  |  |  | Simple mode | | | | | | | 0.97 | | | | | | | | 0.52 | | | | | | | | 2.64 (0.94-7.47) | | | | | | | | 0.103 | | | | | | | |  |  |  |  |  |  |  |  |  |  |  |  |  |  |  |  |  |  |  |  |  |  |  |  |  |  |  |  |  |  |  |  |  |  |  |  |  |  |  |  |  |
|  |  |  |  |  |  |  |  |  |  |  |  |  |  |  |  |  |  |  |  |  |  |  |  |  |  |  | Weighted mode | | | | | | | 0.92 | | | | | | | | 0.49 | | | | | | | | 2.53 (0.96-6.67) | | | | | | | | 0.098 | | | | | | | |  |  |  |  |  |  |  |  |  |  |  |  |  |  |  |  |  |  |  |  |  |  |  |  |  |  |  |  |  |  |  |  |  |  |  |  |  |  |  |  |  |
| genus | | | Sellimonas | | | | | | | | | | | | Alcohol-induced chronic pancreatitis | | | | | | 9 | | | | | | MR-Egger | | | | | | | -0.72 | | | | | | | | 0.84 | | | | | | | | 0.49 (0.09-2.56) | | | | | | | | 0.422 | | | | | | | | 0.24 | | | | | | | | 11.060 | | | | | | | 0.198 | | | | | | 0.1493 | | | | | | | | 0.1223 | | | | | | | | 0.261 | | | |
|  |  |  |  |  |  |  |  |  |  |  |  |  |  |  |  |  |  |  |  |  |  |  |  |  |  |  | Weighted median | | | | | | | 0.03 | | | | | | | | 0.17 | | | | | | | | 1.03 (0.73-1.47) | | | | | | | | 0.852 | | | | | | | |  |  |  |  |  |  |  |  |  |  |  |  |  |  |  |  |  |  |  |  |  |  |  |  |  |  |  |  |  |  |  |  |  |  |  |  |  |  |  |  |  |
|  |  |  |  |  |  |  |  |  |  |  |  |  |  |  |  |  |  |  |  |  |  |  |  |  |  |  | Inverse variance weighted | | | | | | | 0.29 | | | | | | | | 0.14 | | | | | | | | 1.35 (1.01-1.80) | | | | | | | | 0.044 | | | | | | | |  |  |  |  |  |  |  |  |  |  |  |  |  |  |  |  |  |  |  |  |  |  |  |  |  |  |  |  |  |  |  |  |  |  |  |  |  |  |  |  |  |
|  |  |  |  |  |  |  |  |  |  |  |  |  |  |  |  |  |  |  |  |  |  |  |  |  |  |  | Simple mode | | | | | | | -0.06 | | | | | | | | 0.33 | | | | | | | | 0.94 (0.48-1.82) | | | | | | | | 0.853 | | | | | | | |  |  |  |  |  |  |  |  |  |  |  |  |  |  |  |  |  |  |  |  |  |  |  |  |  |  |  |  |  |  |  |  |  |  |  |  |  |  |  |  |  |
|  |  |  |  |  |  |  |  |  |  |  |  |  |  |  |  |  |  |  |  |  |  |  |  |  |  |  | Weighted mode | | | | | | | -0.07 | | | | | | | | 0.30 | | | | | | | | 0.93 (0.51-1.70) | | | | | | | | 0.818 | | | | | | | |  |  |  |  |  |  |  |  |  |  |  |  |  |  |  |  |  |  |  |  |  |  |  |  |  |  |  |  |  |  |  |  |  |  |  |  |  |  |  |  |  |
| genus | | | Slackia | | | | | | | | | | | | Alcohol-induced chronic pancreatitis | | | | | | 6 | | | | | | MR-Egger | | | | | | | 1.16 | | | | | | | | 1.52 | | | | | | | | 3.19 (0.16-62.95) | | | | | | | | 0.488 | | | | | | | | 0.65 | | | | | | | | 3.546 | | | | | | | 0.616 | | | | | | -0.1658 | | | | | | | | 0.151 | | | | | | | | 0.334 | | | |
|  |  |  |  |  |  |  |  |  |  |  |  |  |  |  |  |  |  |  |  |  |  |  |  |  |  |  | Weighted median | | | | | | | -0.43 | | | | | | | | 0.30 | | | | | | | | 0.65 (0.36- 1.19) | | | | | | | | 0.161 | | | | | | | |  |  |  |  |  |  |  |  |  |  |  |  |  |  |  |  |  |  |  |  |  |  |  |  |  |  |  |  |  |  |  |  |  |  |  |  |  |  |  |  |  |
|  |  |  |  |  |  |  |  |  |  |  |  |  |  |  |  |  |  |  |  |  |  |  |  |  |  |  | Inverse variance weighted | | | | | | | -0.48 | | | | | | | | 0.23 | | | | | | | | 0.61 (0.39- 0.97) | | | | | | | | 0.037 | | | | | | | |  |  |  |  |  |  |  |  |  |  |  |  |  |  |  |  |  |  |  |  |  |  |  |  |  |  |  |  |  |  |  |  |  |  |  |  |  |  |  |  |  |
|  |  |  |  |  |  |  |  |  |  |  |  |  |  |  |  |  |  |  |  |  |  |  |  |  |  |  | Simple mode | | | | | | | -0.10 | | | | | | | | 0.43 | | | | | | | | 0.90 (0.39- 2.09) | | | | | | | | 0.813 | | | | | | | |  |  |  |  |  |  |  |  |  |  |  |  |  |  |  |  |  |  |  |  |  |  |  |  |  |  |  |  |  |  |  |  |  |  |  |  |  |  |  |  |  |
|  |  |  |  |  |  |  |  |  |  |  |  |  |  |  |  |  |  |  |  |  |  |  |  |  |  |  | Weighted mode | | | | | | | -0.19 | | | | | | | | 0.46 | | | | | | | | 0.82 (0.33- 2.04) | | | | | | | | 0.692 | | | | | | | |  |  |  |  |  |  |  |  |  |  |  |  |  |  |  |  |  |  |  |  |  |  |  |  |  |  |  |  |  |  |  |  |  |  |  |  |  |  |  |  |  |
| genus | | | Subdoligranulum | | | | | | | | | | | | Alcohol-induced chronic pancreatitis | | | | | | 11 | | | | | | MR-Egger | | | | | | | -0.64 | | | | | | | | 0.70 | | | | | | | | 0.52 (0.13-2.08) | | | | | | | | 0.382 | | | | | | | | 0.44 | | | | | | | | 10.34 | | | | | | | 0.410 | | | | | | 0.0054 | | | | | | | | 0.0513 | | | | | | | | 0.918 | | | |
|  |  |  |  |  |  |  |  |  |  |  |  |  |  |  |  |  |  |  |  |  |  |  |  |  |  |  | Weighted median | | | | | | | -0.85 | | | | | | | | 0.34 | | | | | | | | 0.42 (0.22-0.83) | | | | | | | | 0.012 | | | | | | | |  |  |  |  |  |  |  |  |  |  |  |  |  |  |  |  |  |  |  |  |  |  |  |  |  |  |  |  |  |  |  |  |  |  |  |  |  |  |  |  |  |
|  |  |  |  |  |  |  |  |  |  |  |  |  |  |  |  |  |  |  |  |  |  |  |  |  |  |  | Inverse variance weighted | | | | | | | -0.57 | | | | | | | | 0.25 | | | | | | | | 0.56 (0.34-0.93) | | | | | | | | 0.024 | | | | | | | |  |  |  |  |  |  |  |  |  |  |  |  |  |  |  |  |  |  |  |  |  |  |  |  |  |  |  |  |  |  |  |  |  |  |  |  |  |  |  |  |  |
|  |  |  |  |  |  |  |  |  |  |  |  |  |  |  |  |  |  |  |  |  |  |  |  |  |  |  | Simple mode | | | | | | | -1.00 | | | | | | | | 0.55 | | | | | | | | 0.37 (0.12-1.09) | | | | | | | | 0.101 | | | | | | | |  |  |  |  |  |  |  |  |  |  |  |  |  |  |  |  |  |  |  |  |  |  |  |  |  |  |  |  |  |  |  |  |  |  |  |  |  |  |  |  |  |
|  |  |  |  |  |  |  |  |  |  |  |  |  |  |  |  |  |  |  |  |  |  |  |  |  |  |  | Weighted mode | | | | | | | -0.98 | | | | | | | | 0.50 | | | | | | | | 0.38 (0.14-1.01) | | | | | | | | 0.080 | | | | | | | |  |  |  |  |  |  |  |  |  |  |  |  |  |  |  |  |  |  |  |  |  |  |  |  |  |  |  |  |  |  |  |  |  |  |  |  |  |  |  |  |  |
| **Supplementary Table 8:** In reverse MR results and sensitivity analysis of ACP on gut microbiota. | | | | | | | | | | | | | | | | | | | | | | | | | | | | | | | | | | | | | | | | | | | | | | | | | | | | | | | | | | | | | | | | | | | | | | | | | | | | | | | | | | | | | | | | | | | | | | | | | | | | | | | | | | |
| **Taxa** | | | | | **exposure** | | | | | | | **outcome** | | | | | | | | **Nsnp** | | | | | | **Methods** | | | | | | | **Beta** | | | | | | | | **SE** | | | | | | | | **OR (95%CI)** | | | | | | | | ***P* value** | | | | | | | | **MR-PRESSO** | | | | | | | | **Heterogeneity** | | | | | | | | | | | | | | | **Horizontal pleiotrop** | | | | | | | | | | | | | | | | | | |
|  |  |  |  |  |  |  |  |  |  |  |  |  |  |  |  |  |  |  |  |  |  |  |  |  |  |  |  |  |  |  |  |  |  |  |  |  |  |  |  |  |  |  |  |  |  |  |  |  |  |  |  |  |  |  |  |  |  |  |  |  |  |  |  |  |  |  |  |  |  |  |  |  | **Cochran’s Q** | | | | | | | | ***P* value** | | | | | | | **Egger intercept** | | | | | | | | **SE** | | | | | | | | ***P* value** | | |
| class | | | | | Alcohol-induced chronic pancreatitis | | | | | | | Negativicutes | | | | | | | | 10 | | | | | | MR-Egger | | | | | | | 0.01 | | | | | | | | 0.04 | | | | | | | | 1.01 (0.93-1.11) | | | | | | | | 0.744 | | | | | | | | 0.85 | | | | | | | | 4.962 | | | | | | | | 0.837 | | | | | | | -0.013 | | | | | | | | 0.012 | | | | | | | | 0.316 | | |
|  |  |  |  |  |  |  |  |  |  |  |  |  |  |  |  |  |  |  |  |  |  |  |  |  |  | Weighted median | | | | | | | -0.02 | | | | | | | | 0.01 | | | | | | | | 0.97 (0.94-1.01) | | | | | | | | 0.158 | | | | | | | |  |  |  |  |  |  |  |  |  |  |  |  |  |  |  |  |  |  |  |  |  |  |  |  |  |  |  |  |  |  |  |  |  |  |  |  |  |  |  |  |  |  |
|  |  |  |  |  |  |  |  |  |  |  |  |  |  |  |  |  |  |  |  |  |  |  |  |  |  | Inverse variance weighted | | | | | | | -0.02 | | | | | | | | 0.01 | | | | | | | | 0.97 (0.94-1.00) | | | | | | | | 0.046 | | | | | | | |  |  |  |  |  |  |  |  |  |  |  |  |  |  |  |  |  |  |  |  |  |  |  |  |  |  |  |  |  |  |  |  |  |  |  |  |  |  |  |  |  |  |
|  |  |  |  |  |  |  |  |  |  |  |  |  |  |  |  |  |  |  |  |  |  |  |  |  |  | Simple mode | | | | | | | -0.03 | | | | | | | | 0.03 | | | | | | | | 0.97 (0.91-1.03) | | | | | | | | 0.314 | | | | | | | |  |  |  |  |  |  |  |  |  |  |  |  |  |  |  |  |  |  |  |  |  |  |  |  |  |  |  |  |  |  |  |  |  |  |  |  |  |  |  |  |  |  |
|  |  |  |  |  |  |  |  |  |  |  |  |  |  |  |  |  |  |  |  |  |  |  |  |  |  | Weighted mode | | | | | | | -0.02 | | | | | | | | 0.02 | | | | | | | | 0.98 (0.92-1.04) | | | | | | | | 0.464 | | | | | | | |  |  |  |  |  |  |  |  |  |  |  |  |  |  |  |  |  |  |  |  |  |  |  |  |  |  |  |  |  |  |  |  |  |  |  |  |  |  |  |  |  |  |
| order | | | | | Alcohol-induced chronic pancreatitis | | | | | | | Selenomonadales | | | | | | | | 10 | | | | | | MR-Egger | | | | | | | 0.01 | | | | | | | | 0.04 | | | | | | | | 1.01 (0.93-1.11) | | | | | | | | 0.744 | | | | | | | | 0.87 | | | | | | | | 4.962 | | | | | | | | 0.8375 | | | | | | | -0.013 | | | | | | | | 0.0123 | | | | | | | | 0.316 | | |
|  |  |  |  |  |  |  |  |  |  |  |  |  |  |  |  |  |  |  |  |  |  |  |  |  |  | Weighted median | | | | | | | -0.02 | | | | | | | | 0.01 | | | | | | | | 0.97 (0.94-1.01) | | | | | | | | 0.181 | | | | | | | |  |  |  |  |  |  |  |  |  |  |  |  |  |  |  |  |  |  |  |  |  |  |  |  |  |  |  |  |  |  |  |  |  |  |  |  |  |  |  |  |  |  |
|  |  |  |  |  |  |  |  |  |  |  |  |  |  |  |  |  |  |  |  |  |  |  |  |  |  | Inverse variance weighted | | | | | | | -0.02 | | | | | | | | 0.01 | | | | | | | | 0.97 (0.94-1.00) | | | | | | | | 0.046 | | | | | | | |  |  |  |  |  |  |  |  |  |  |  |  |  |  |  |  |  |  |  |  |  |  |  |  |  |  |  |  |  |  |  |  |  |  |  |  |  |  |  |  |  |  |
|  |  |  |  |  |  |  |  |  |  |  |  |  |  |  |  |  |  |  |  |  |  |  |  |  |  | Simple mode | | | | | | | -0.03 | | | | | | | | 0.03 | | | | | | | | 0.97 (0.90-1.03) | | | | | | | | 0.328 | | | | | | | |  |  |  |  |  |  |  |  |  |  |  |  |  |  |  |  |  |  |  |  |  |  |  |  |  |  |  |  |  |  |  |  |  |  |  |  |  |  |  |  |  |  |
|  |  |  |  |  |  |  |  |  |  |  |  |  |  |  |  |  |  |  |  |  |  |  |  |  |  | Weighted mode | | | | | | | -0.02 | | | | | | | | 0.03 | | | | | | | | 0.98 (0.92-1.04) | | | | | | | | 0.496 | | | | | | | |  |  |  |  |  |  |  |  |  |  |  |  |  |  |  |  |  |  |  |  |  |  |  |  |  |  |  |  |  |  |  |  |  |  |  |  |  |  |  |  |  |  |
| genus | | | | | Alcohol-induced chronic pancreatitis | | | | | | | Butyrivibrio | | | | | | | | 9 | | | | | | MR-Egger | | | | | | | -0.01 | | | | | | | | 0.13 | | | | | | | | 0.98 (0.75-1.29) | | | | | | | | 0.908 | | | | | | | | 0.5 | | | | | | | | 8.2737 | | | | | | | | 0.407 | | | | | | | -0.0263 | | | | | | | | 0.0367 | | | | | | | | 0.4968 | | |
|  |  |  |  |  |  |  |  |  |  |  |  |  |  |  |  |  |  |  |  |  |  |  |  |  |  | Weighted median | | | | | | | -0.11 | | | | | | | | 0.05 | | | | | | | | 0.89 (0.81-0.99) | | | | | | | | 0.023 | | | | | | | |  |  |  |  |  |  |  |  |  |  |  |  |  |  |  |  |  |  |  |  |  |  |  |  |  |  |  |  |  |  |  |  |  |  |  |  |  |  |  |  |  |  |
|  |  |  |  |  |  |  |  |  |  |  |  |  |  |  |  |  |  |  |  |  |  |  |  |  |  | Inverse variance weighted | | | | | | | -0.11 | | | | | | | | 0.03 | | | | | | | | 0.89 (0.83-0.96) | | | | | | | | 0.002 | | | | | | | |  |  |  |  |  |  |  |  |  |  |  |  |  |  |  |  |  |  |  |  |  |  |  |  |  |  |  |  |  |  |  |  |  |  |  |  |  |  |  |  |  |  |
|  |  |  |  |  |  |  |  |  |  |  |  |  |  |  |  |  |  |  |  |  |  |  |  |  |  | Simple mode | | | | | | | -0.11 | | | | | | | | 0.07 | | | | | | | | 0.89 (0.77-1.02) | | | | | | | | 0.128 | | | | | | | |  |  |  |  |  |  |  |  |  |  |  |  |  |  |  |  |  |  |  |  |  |  |  |  |  |  |  |  |  |  |  |  |  |  |  |  |  |  |  |  |  |  |
|  |  |  |  |  |  |  |  |  |  |  |  |  |  |  |  |  |  |  |  |  |  |  |  |  |  | Weighted mode | | | | | | | -0.11 | | | | | | | | 0.06 | | | | | | | | 0.89 (0.78-1.02) | | | | | | | | 0.128 | | | | | | | |  |  |  |  |  |  |  |  |  |  |  |  |  |  |  |  |  |  |  |  |  |  |  |  |  |  |  |  |  |  |  |  |  |  |  |  |  |  |  |  |  |  |
| genus | | | | | Alcohol-induced chronic pancreatitis | | | | | | | Ruminiclostridium5 | | | | | | | | 10 | | | | | | MR-Egger | | | | | | | 0.001 | | | | | | | | 0.04 | | | | | | | | 1.01 (0.93-1.10) | | | | | | | | 0.839 | | | | | | | | 0.85 | | | | | | | | 4.920 | | | | | | | | 0.841 | | | | | | | -0.0148 | | | | | | | | 0.0125 | | | | | | | | 0.2714 | | |
|  |  |  |  |  |  |  |  |  |  |  |  |  |  |  |  |  |  |  |  |  |  |  |  |  |  | Weighted median | | | | | | | -0.03 | | | | | | | | 0.02 | | | | | | | | 0.97 (0.93-1.01) | | | | | | | | 0.087 | | | | | | | |  |  |  |  |  |  |  |  |  |  |  |  |  |  |  |  |  |  |  |  |  |  |  |  |  |  |  |  |  |  |  |  |  |  |  |  |  |  |  |  |  |  |
|  |  |  |  |  |  |  |  |  |  |  |  |  |  |  |  |  |  |  |  |  |  |  |  |  |  | Inverse variance weighted | | | | | | | -0.04 | | | | | | | | 0.01 | | | | | | | | 0.96 (0.93-0.99) | | | | | | | | 0.007 | | | | | | | |  |  |  |  |  |  |  |  |  |  |  |  |  |  |  |  |  |  |  |  |  |  |  |  |  |  |  |  |  |  |  |  |  |  |  |  |  |  |  |  |  |  |
|  |  |  |  |  |  |  |  |  |  |  |  |  |  |  |  |  |  |  |  |  |  |  |  |  |  | Simple mode | | | | | | | -0.03 | | | | | | | | 0.03 | | | | | | | | 0.97 (0.91-1.03) | | | | | | | | 0.358 | | | | | | | |  |  |  |  |  |  |  |  |  |  |  |  |  |  |  |  |  |  |  |  |  |  |  |  |  |  |  |  |  |  |  |  |  |  |  |  |  |  |  |  |  |  |
|  |  |  |  |  |  |  |  |  |  |  |  |  |  |  |  |  |  |  |  |  |  |  |  |  |  | Weighted mode | | | | | | | -0.02 | | | | | | | | 0.02 | | | | | | | | 0.98 (0.93-1.04) | | | | | | | | 0.480 | | | | | | | |  |  |  |  |  |  |  |  |  |  |  |  |  |  |  |  |  |  |  |  |  |  |  |  |  |  |  |  |  |  |  |  |  |  |  |  |  |  |  |  |  |  |
