# Supplementary material for: Causal link between gut microbiota and four types of pancreatitis: a genetic association and bidirectional Mendelian randomization study
Source: Front Microbiol. 2023 Nov 23;14:1290202. doi: 10.3389/fmicb.2023.1290202 (PMC10702359; doi:10.3389/fmicb.2023.1290202)
Supplement: Supplementary file 3 [file Table_3.DOCX]

**Supplementary Figure legends**

**Supplementary Figure 1:** Plots for "leave-one-out" analysis for causal effect of gut microbiota on AP(A-F), CP (G-L) risk, Leave-one-out plot helps determine whether the overall effect is altered by one or more specific genetic variants by sequentially re-evaluating causal estimates after discarding one SNP at a time. Within each panel, the black points represent the causal estimate of association between a specific exposure and target pancreatitis after discarding each SNP in turn. Red points represent the overall causal estimate using the random-effects inverse variance weighted. Horizontal lines denote 95% confidence intervals. The plots reveal no single SNP distorting overall MR estimates when each SNP is removed from principal MR analyses.

**Supplementary Figure 2:** Plots for "leave-one-out" analysis for causal effect of gut microbiota on AAP(A-E), ACP (F-L) risk, Leave-one-out plot helps determine whether the overall effect is altered by one or more specific genetic variants by sequentially re-evaluating causal estimates after discarding one SNP at a time. Within each panel, the black points represent the causal estimate of association between a specific exposure and target pancreatitis after discarding each SNP in turn. Red points represent the overall causal estimate using the random-effects inverse variance weighted. Horizontal lines denote 95% confidence intervals. The plots reveal no single SNP distorting overall MR estimates when each SNP is removed from principal MR analyses.

**Supplementary Figure 3:** Plots for "leave-one-out" analysis for causal effect of gut microbiota on ACP (A-D) risk. in reverse MR analysis, Plots for "leave-one-out" analysis for causal effect of AP (E-I), CP(J-L) on gut microbiota risk, Leave-one-out plot helps determine whether the overall effect is altered by one or more specific genetic variants by sequentially re-evaluating causal estimates after discarding one SNP at a time. Within each panel, the black points represent the causal estimate of association between a specific exposure and target pancreatitis after discarding each SNP in turn. Red points represent the overall causal estimate using the random-effects inverse variance weighted. Horizontal lines denote 95% confidence intervals. The plots reveal no single SNP distorting overall MR estimates when each SNP is removed from principal MR analyses.

**Supplementary Figure 4**: In reverse MR analysis, Plots for "leave-one-out" analysis for causal effect of CP (A-D), AAP(E-L) on gut microbiota risk, Leave-one-out plot helps determine whether the overall effect is altered by one or more specific genetic variants by sequentially re-evaluating causal estimates after discarding one SNP at a time. Within each panel, the black points represent the causal estimate of association between a specific exposure and target pancreatitis after discarding each SNP in turn. Red points represent the overall causal estimate using the random-effects inverse variance weighted. Horizontal lines denote 95% confidence intervals. The plots reveal no single SNP distorting overall MR estimates when each SNP is removed from principal MR analyses.

**Supplementary Figure 5:** In reverse MR analysis, Plots for "leave-one-out" analysis for causal effect of AAP (A-F), ACP(G-J) on gut microbiota risk, Leave-one-out plot helps determine whether the overall effect is altered by one or more specific genetic variants by sequentially re-evaluating causal estimates after discarding one SNP at a time. Within each panel, the black points represent the causal estimate of association between a specific exposure and target pancreatitis after discarding each SNP in turn. Red points represent the overall causal estimate using the random-effects inverse variance weighted. Horizontal lines denote 95% confidence intervals. The plots reveal no single SNP distorting overall MR estimates when each SNP is removed from principal MR analyses.

**Supplementary Figure 6:** The scatter plots for association between gut microbiota and AP(A-F), CP(G-L).

**Supplementary Figure 7:** The scatter plots for association between gut microbiota and AAP(A-E), ACP(F-L).

**Supplementary Figure 8:** The scatter plots for association between gut microbiota and ACP(A-D), In reverse MR analysis, The scatter plots for association between AP(E-I), CP(J-L) and gut microbiota.

**Supplementary Figure 9:** In reverse MR analysis, The scatter plots for association between CP(A-E), AAP(E-L) and gut microbiota.

**Supplementary Figure 10:** In reverse MR analysis, The scatter plots for association between AAP(A-F), ACP(G-J) and gut microbiota.

**Supplementary Figure 11:** Forest plots for causal effects of gut microbiota on AP (A-F), CP(D-I) risk with individual SNPs.

**Supplementary Figure 12:** Forest plots for causal effects of gut microbiota on AAP (A-E), ACP(F-L) risk with individual SNPs.

**Supplementary Figure 13:** Forest plots for causal effects of gut microbiota on ACP (A-D) risk with individual SNPs, In reverse MR analysis, The Forest plots for association between AP(E-I), CP(J-L) and gut microbiota.

**Supplementary Figure 14:** In reverse MR analysis, The Forest plots for association between CP(A-D), AAP(E-L) and gut microbiota.

**Supplementary Figure 15:** In reverse MR analysis, The Forest plots for association between AAP(A-F), ACP(G-J) and gut microbiota.

**Supplementary Table S1:** Instrumental variables used in MR analysis of the association between gut microbiota and four types of pancreatitis.

**Supplementary Table S2:** In reverse MR analysis, Instrumental variables used in MR analysis of the association between four types of pancreatitis and gut microbiota.

**Supplementary Table 1**: MR results and sensitivity analysis of gut microbiota on AP. Significant threshold was set at p-value <0.05 for the Inverse Variance Weighted method (IVW). IVs, Instrumental Variables; SE, standard error, MR-Egger and Cochran’s Q test of gut microbiome on AC in MR analysis, MR-PRESSO, MR pleiotropy residual sum and outlier.

**Supplementary Table 2:** In reverse MR results and sensitivity analysis of AP on gut microbiota. Significant threshold was set at p-value <0.05 for the Inverse Variance Weighted method (IVW). IVs, Instrumental Variables; SE, standard error, MR-Egger and Cochran’s Q test of gut microbiome on AC in MR analysis, MR-PRESSO, MR pleiotropy residual sum and outlier.

**Supplementary Table 3**:MR results and sensitivity analysis of gut microbiota on CP. Significant threshold was set at p-value <0.05 for the Inverse Variance Weighted method (IVW). IVs, Instrumental Variables; SE, standard error, MR-Egger and Cochran’s Q test of gut microbiome on AC in MR analysis, MR-PRESSO, MR pleiotropy residual sum and outlier.

**Supplementary Table 4**: In reverse MR results and sensitivity analysis of CP on gut microbiota. Significant threshold was set at p-value <0.05 for the Inverse Variance Weighted method (IVW). IVs, Instrumental Variables; SE, standard error, MR-Egger and Cochran’s Q test of gut microbiome on AC in MR analysis, MR-PRESSO, MR pleiotropy residual sum and outlier.

**Supplementary Table 5:**MR results and sensitivity analysis of gut microbiota on AAP.

**Supplementary Table 6:** In reverse MR results and sensitivity analysis of AAP on gut microbiota. Significant threshold was set at p-value <0.05 for the Inverse Variance Weighted method (IVW). IVs, Instrumental Variables; SE, standard error, MR-Egger and Cochran’s Q test of gut microbiome on AC in MR analysis, MR-PRESSO, MR pleiotropy residual sum and outlier.

**Supplementary Table 7**:MR results and sensitivity analysis of gut microbiota on ACP. Significant threshold was set at p-value <0.05 for the Inverse Variance Weighted method (IVW). IVs, Instrumental Variables; SE, standard error, MR-Egger and Cochran’s Q test of gut microbiome on AC in MR analysis, MR-PRESSO, MR pleiotropy residual sum and outlier.

**Supplementary Table 8:** In reverse MR results and sensitivity analysis of ACP on gut microbiota. Significant threshold was set at p-value <0.05 for the Inverse Variance Weighted method (IVW). IVs, Instrumental Variables; SE, standard error, MR-Egger and Cochran’s Q test of gut microbiome on AC in MR analysis, MR-PRESSO, MR pleiotropy residual sum and outlier.
