# Supplementary figures and images for: Causal link between gut microbiota and four types of pancreatitis: a genetic association and bidirectional Mendelian randomization study
Source: Front Microbiol. 2023 Nov 23;14:1290202. doi: 10.3389/fmicb.2023.1290202 (PMC10702359; doi:10.3389/fmicb.2023.1290202)

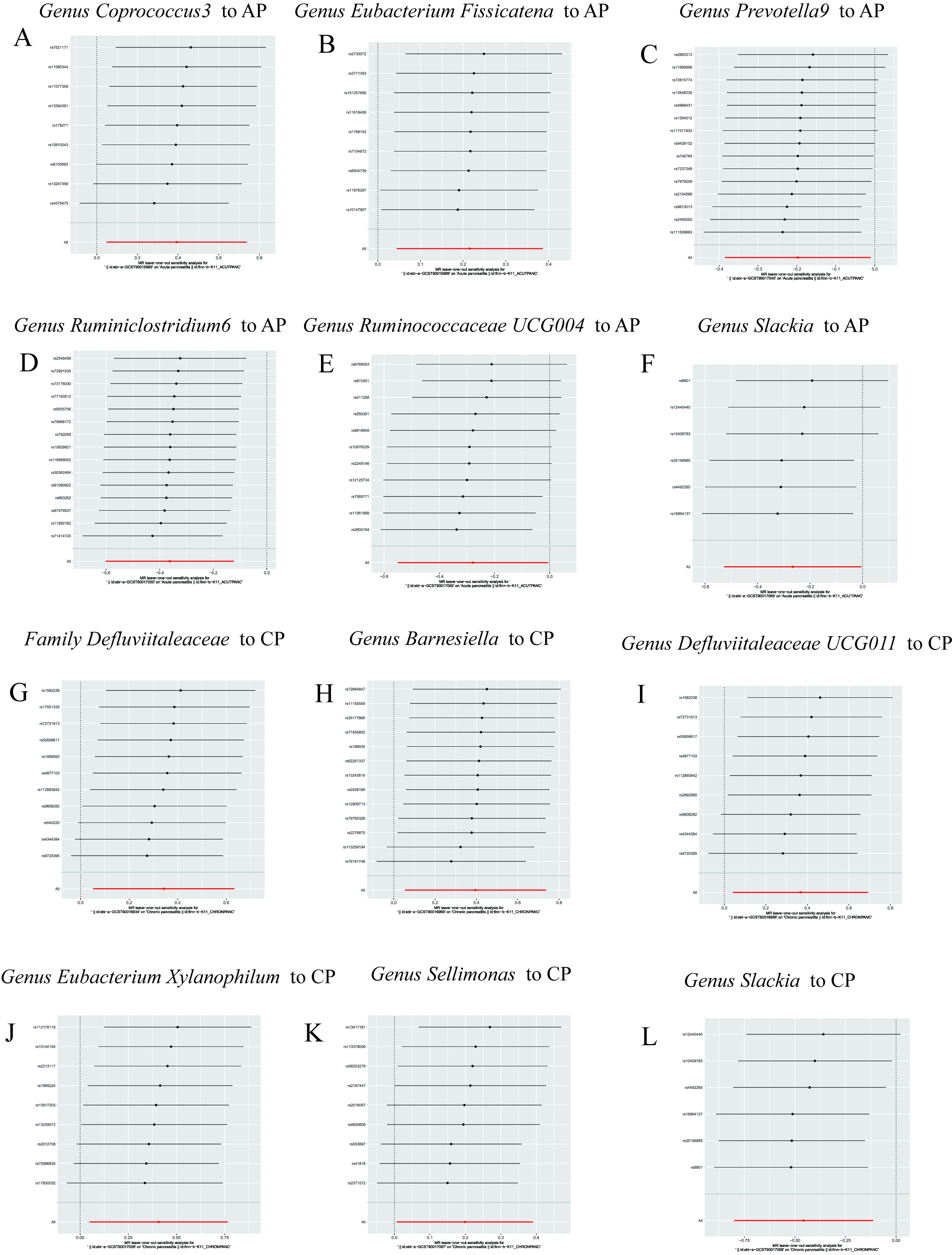

Supplement: Supplementary file 4 [file Image_1.TIF]

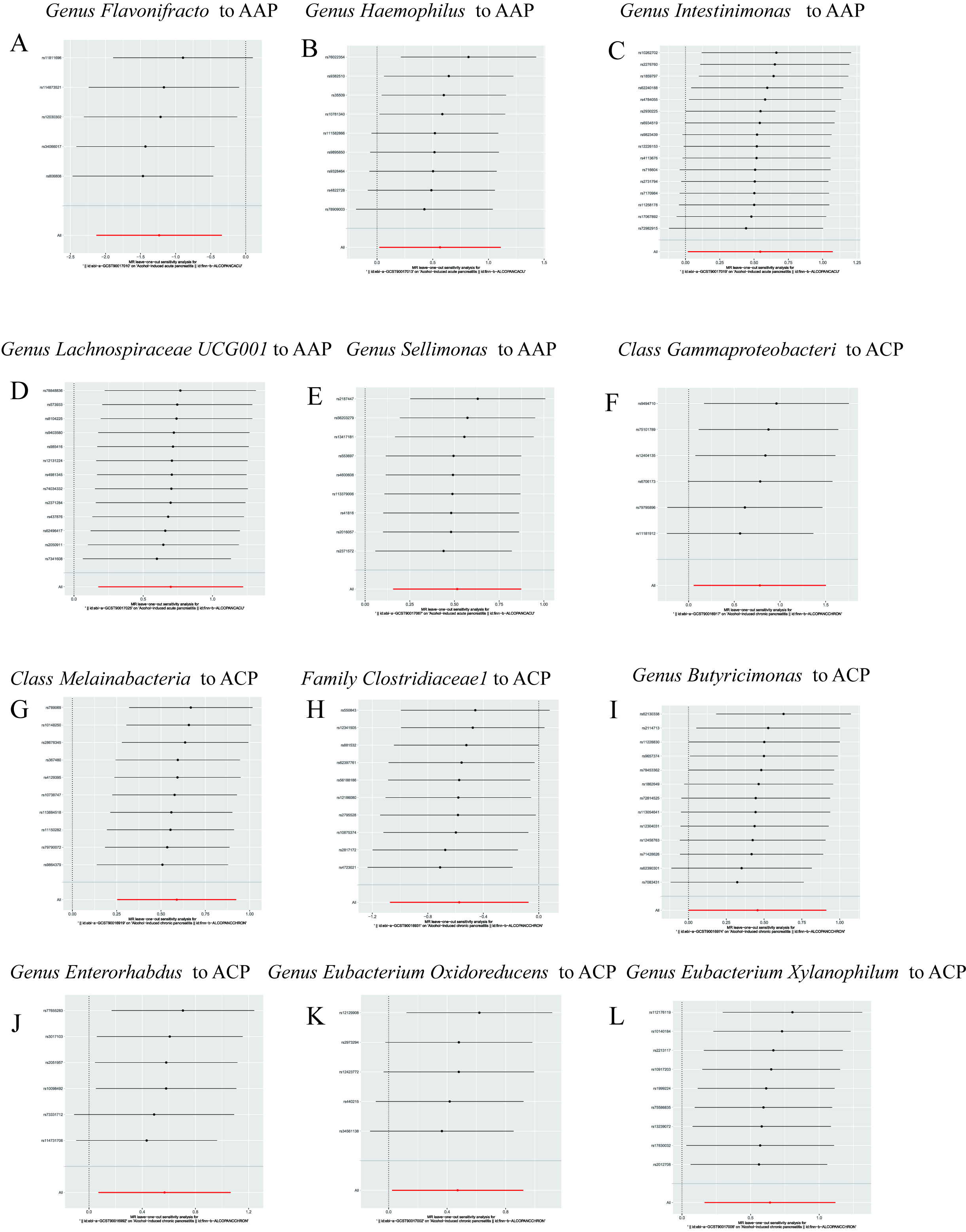

Supplement: Supplementary file 5 [file Image_2.TIF]

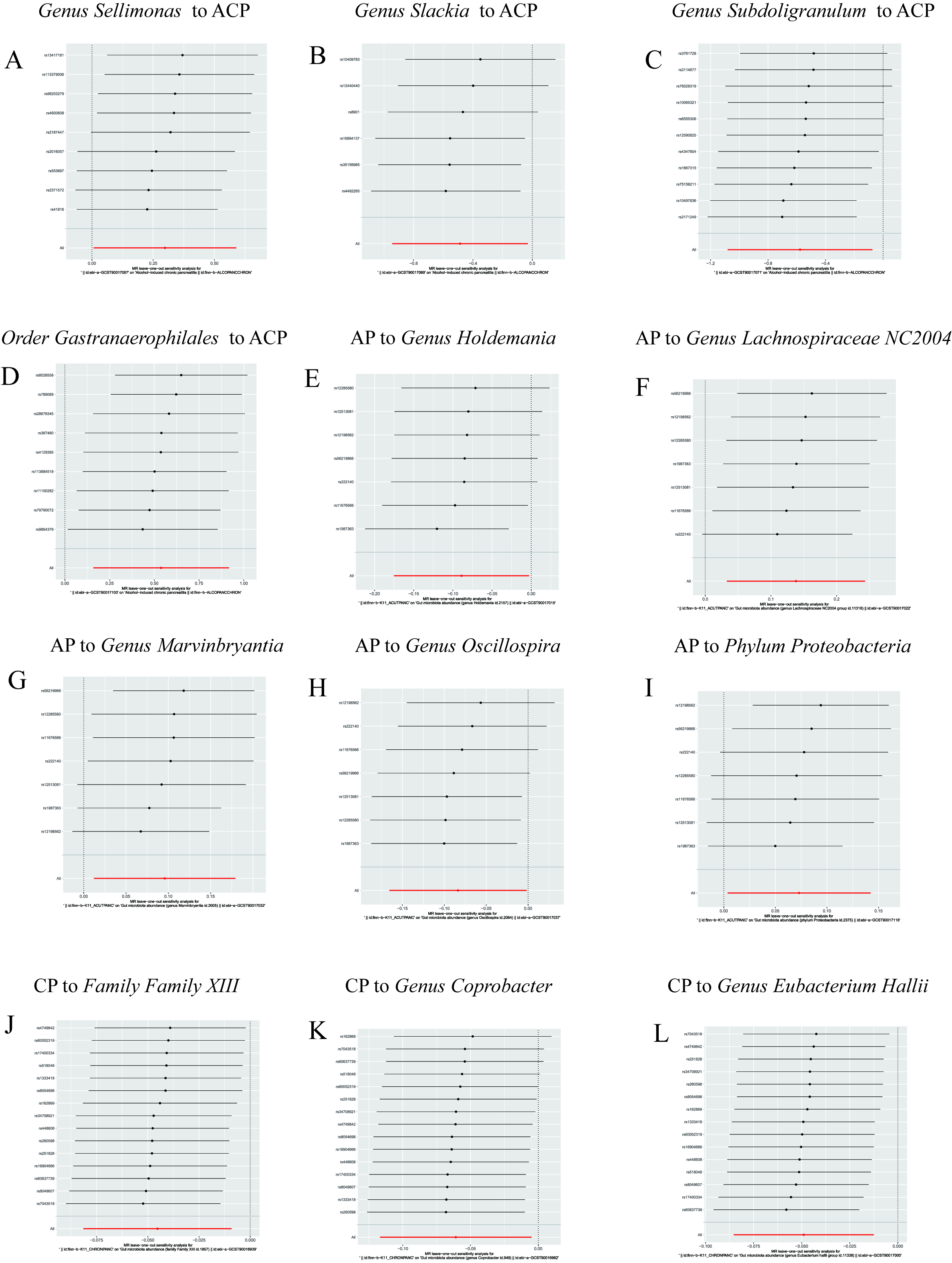

Supplement: Supplementary file 6 [file Image_3.TIF]

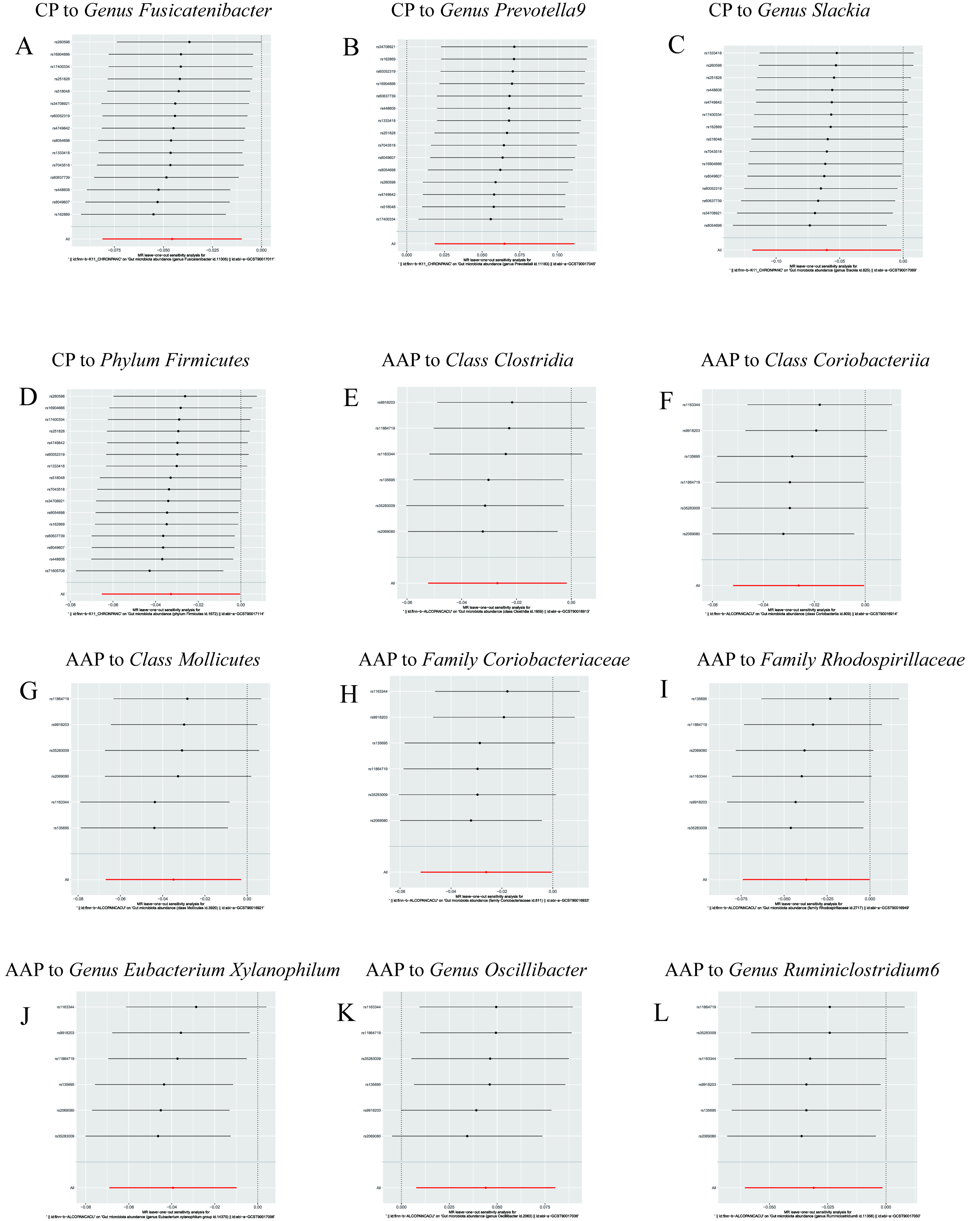

Supplement: Supplementary file 7 [file Image_4.TIF]

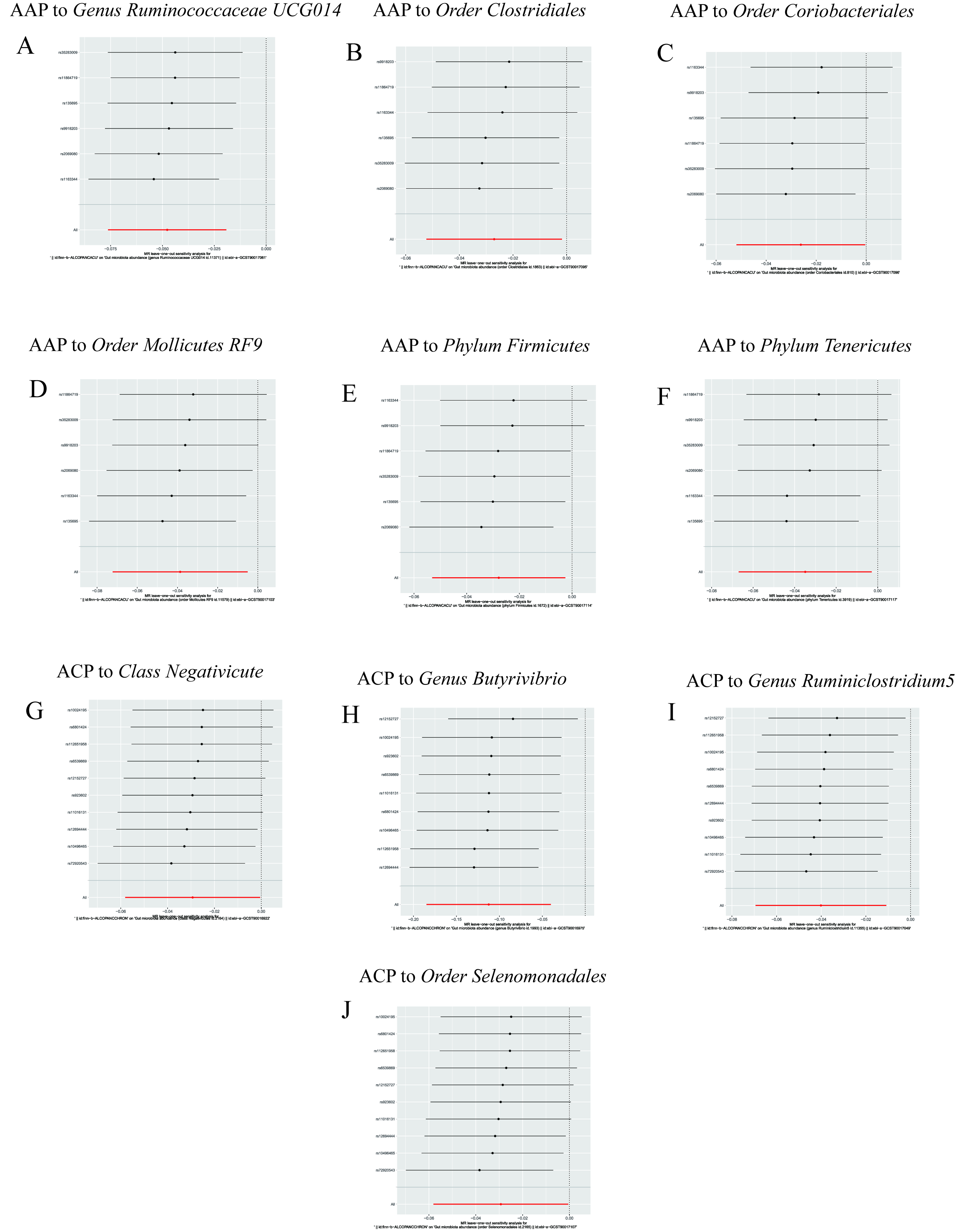

Supplement: Supplementary file 8 [file Image_5.TIF]

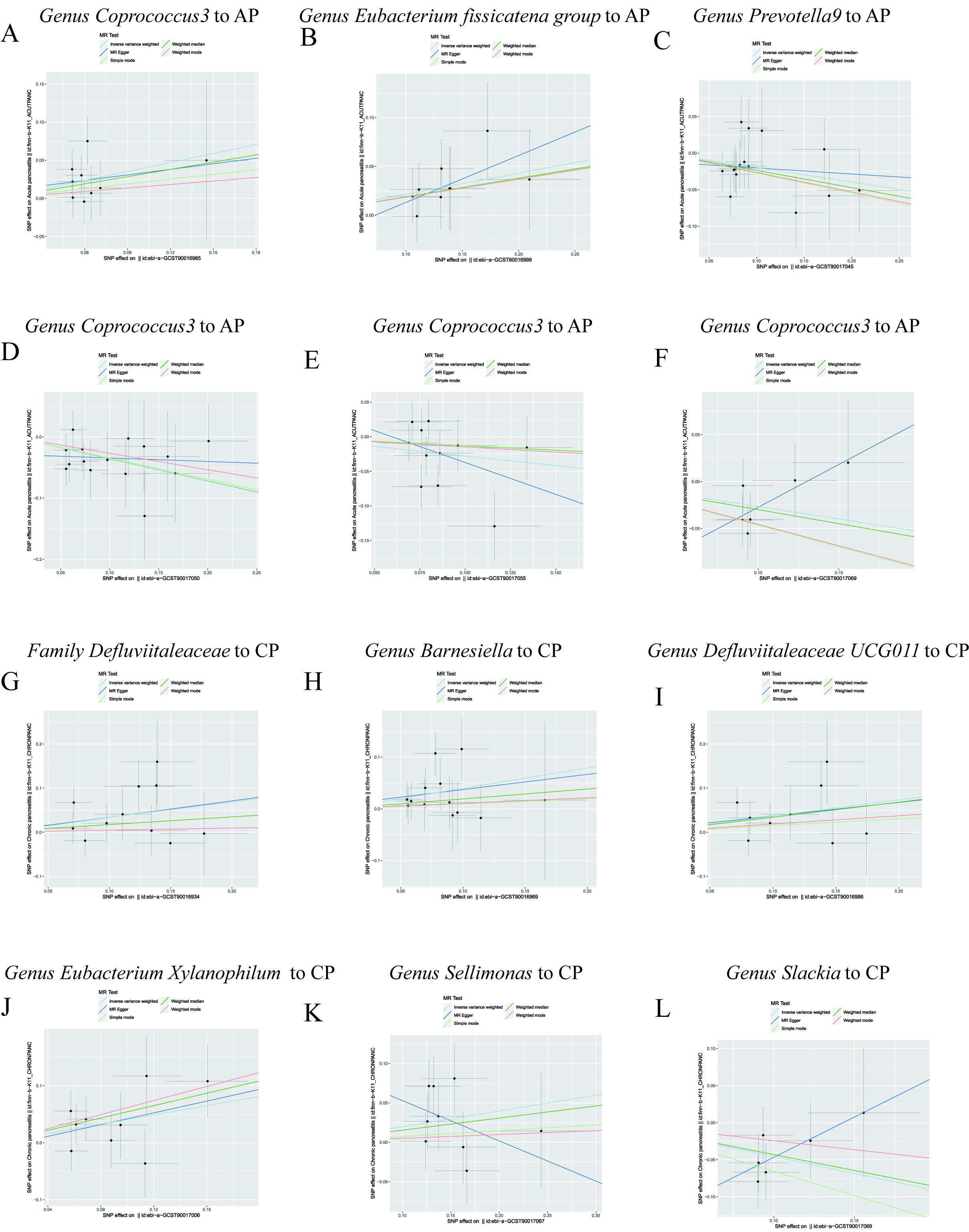

Supplement: Supplementary file 9 [file Image_6.TIF]

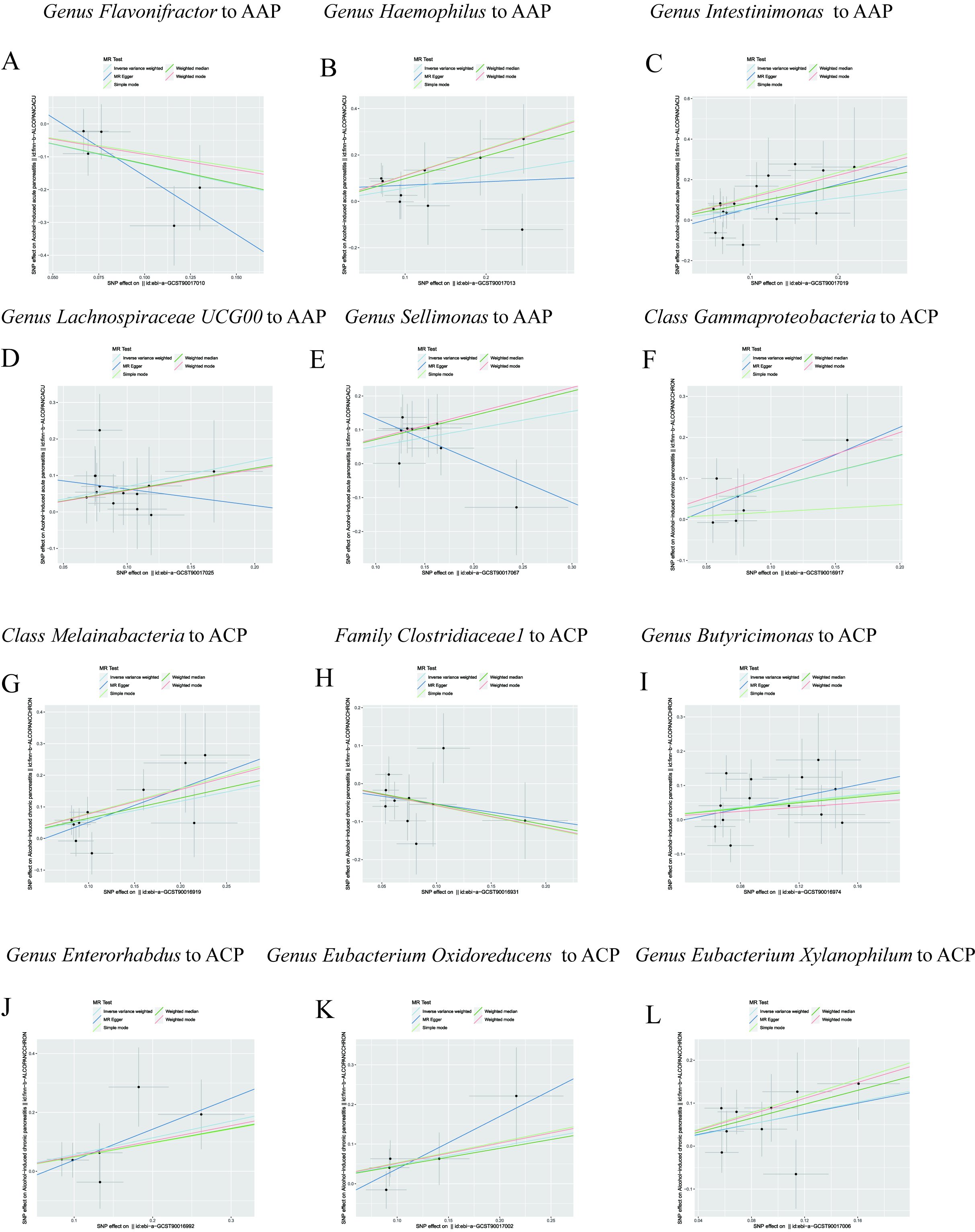

Supplement: Supplementary file 10 [file Image_7.TIF]

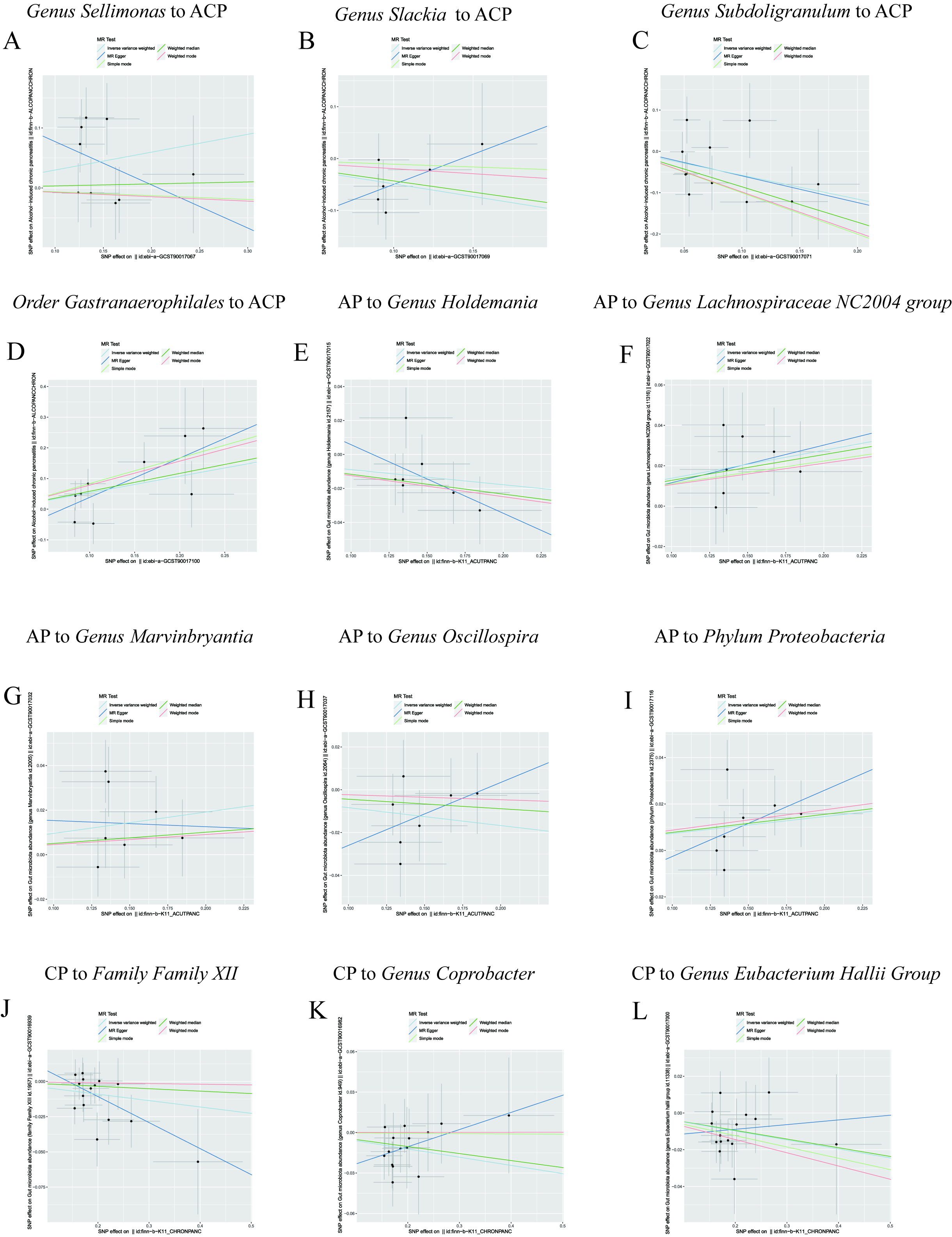

Supplement: Supplementary file 11 [file Image_8.TIF]

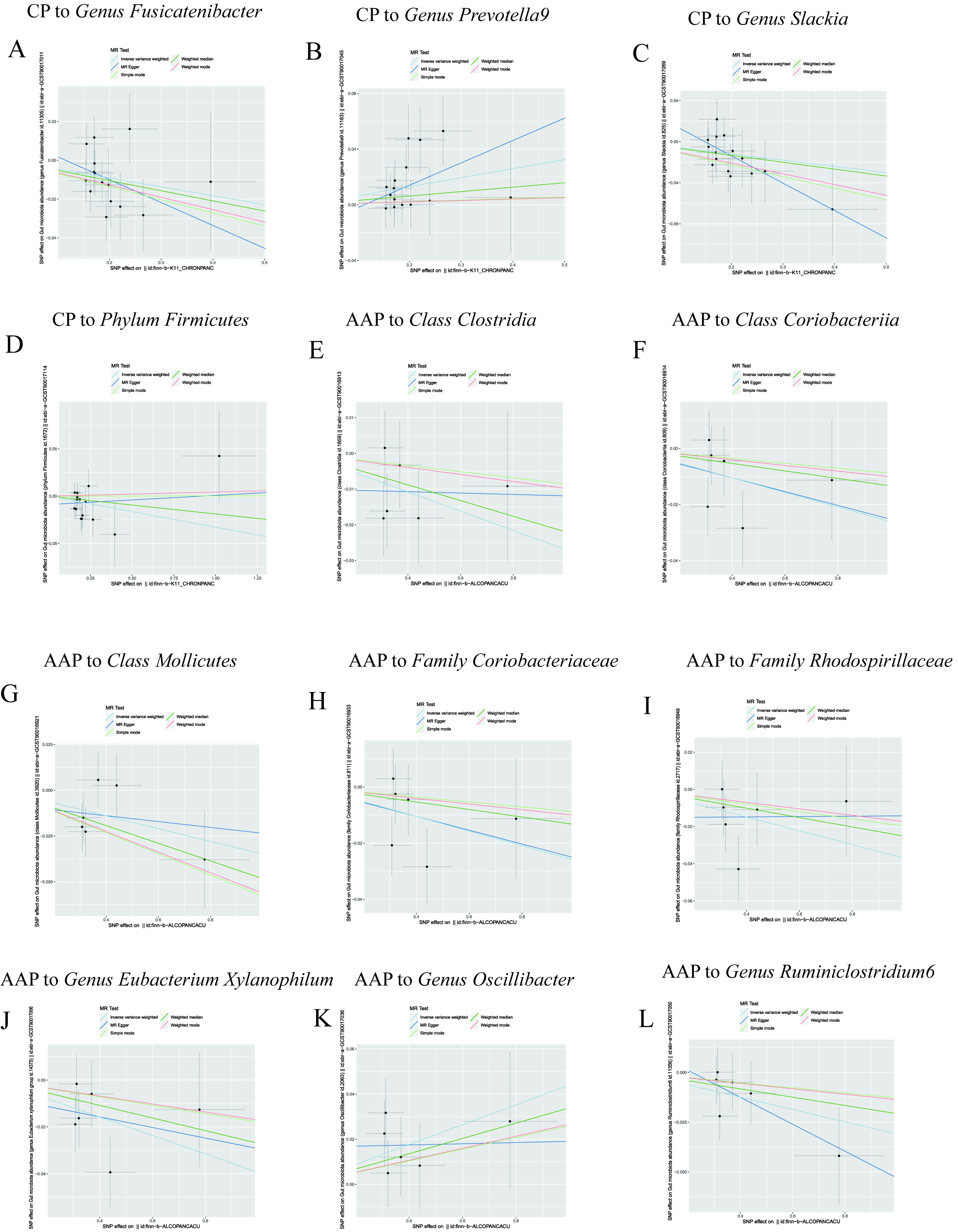

Supplement: Supplementary file 12 [file Image_9.TIF]

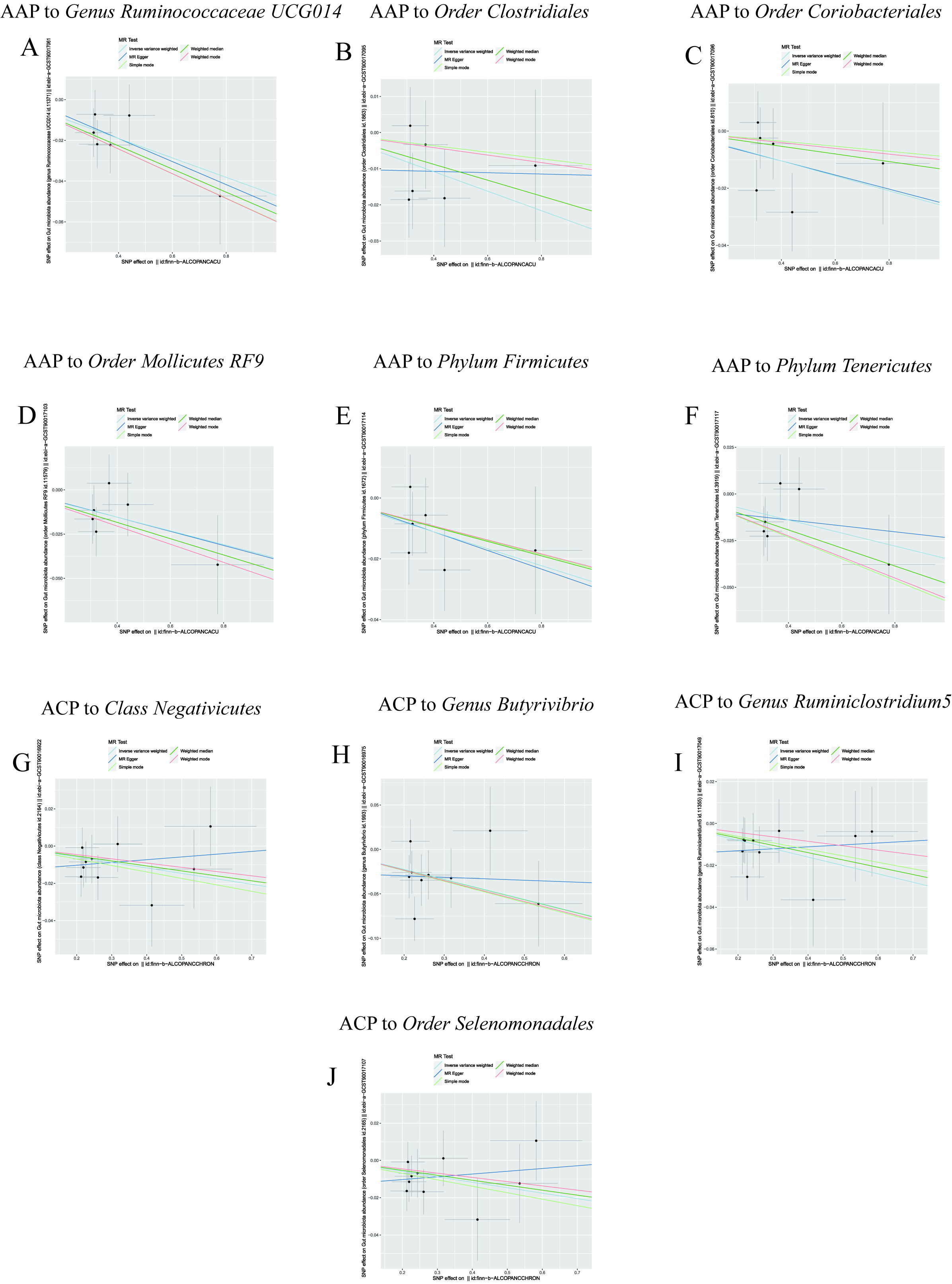

Supplement: Supplementary file 13 [file Image_10.TIF]

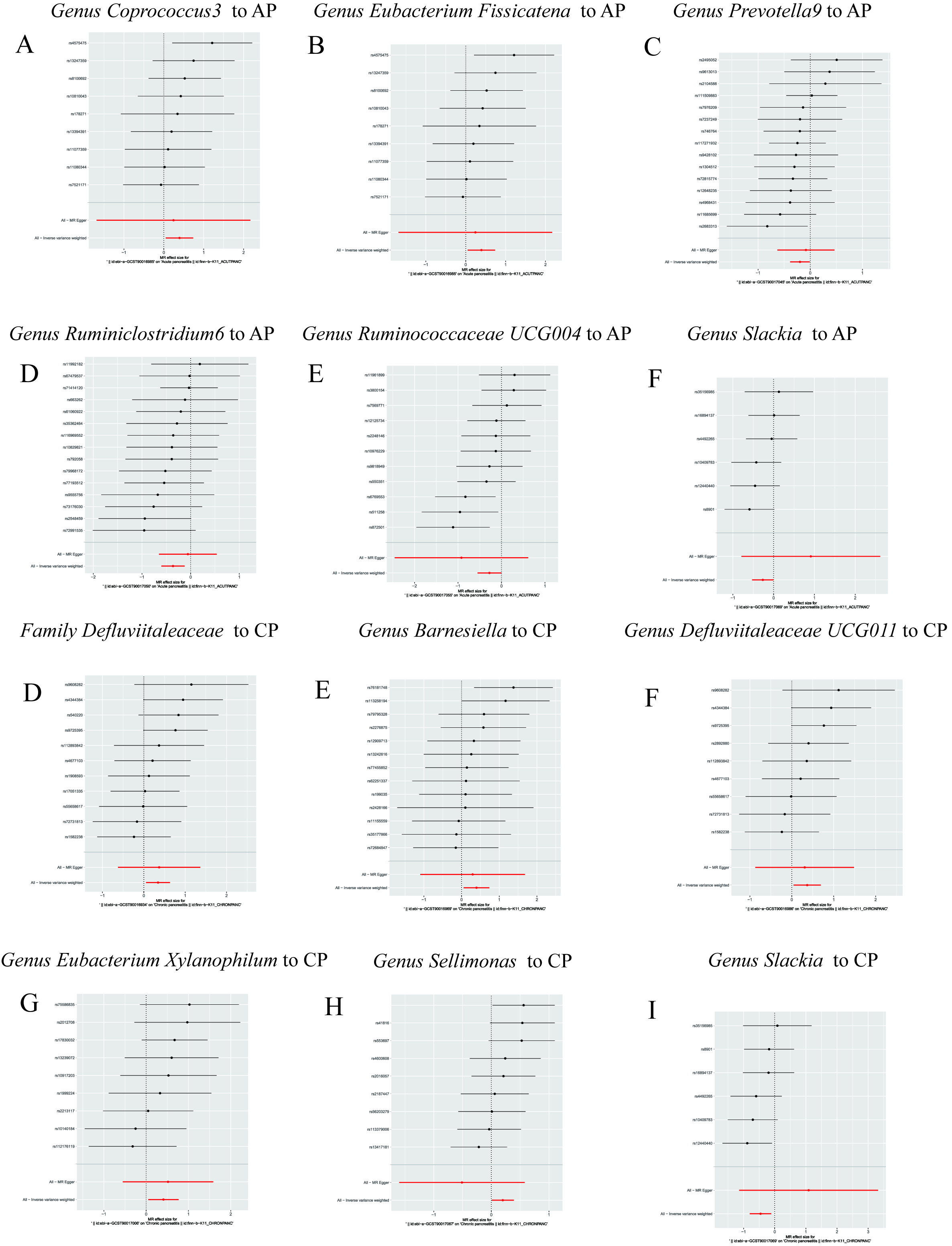

Supplement: Supplementary file 14 [file Image_11.TIF]

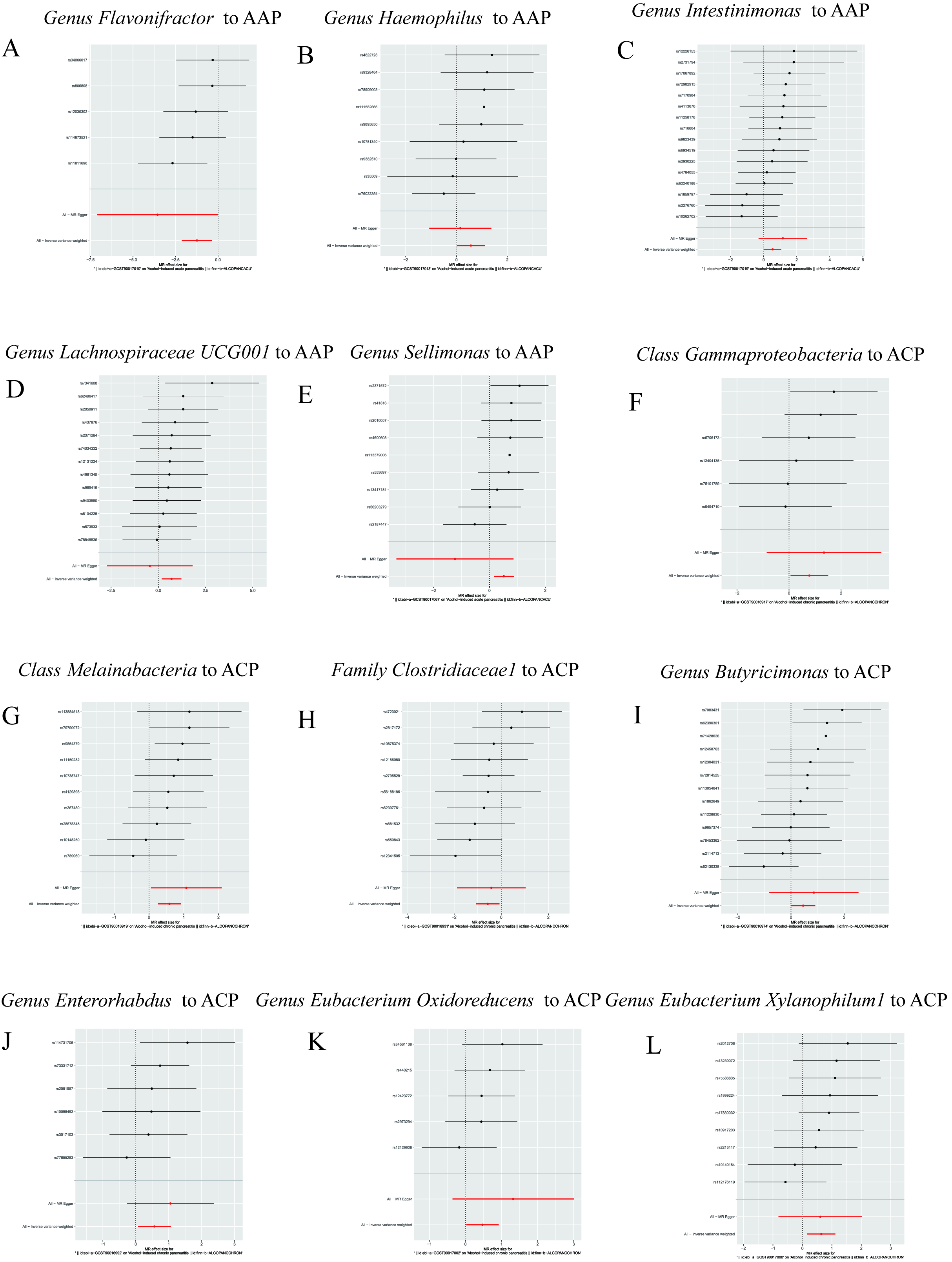

Supplement: Supplementary file 15 [file Image_12.TIF]

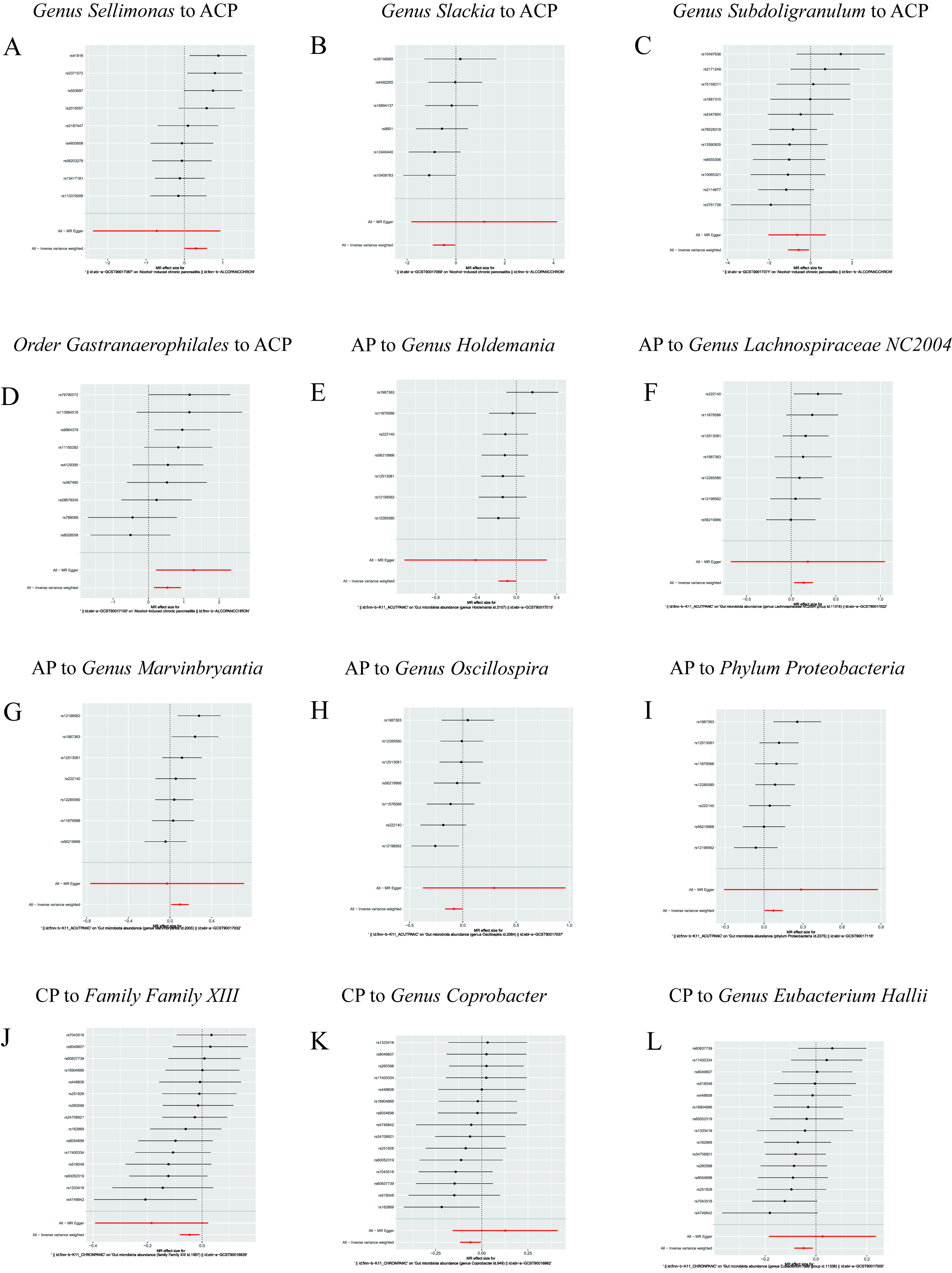

Supplement: Supplementary file 16 [file Image_13.TIF]

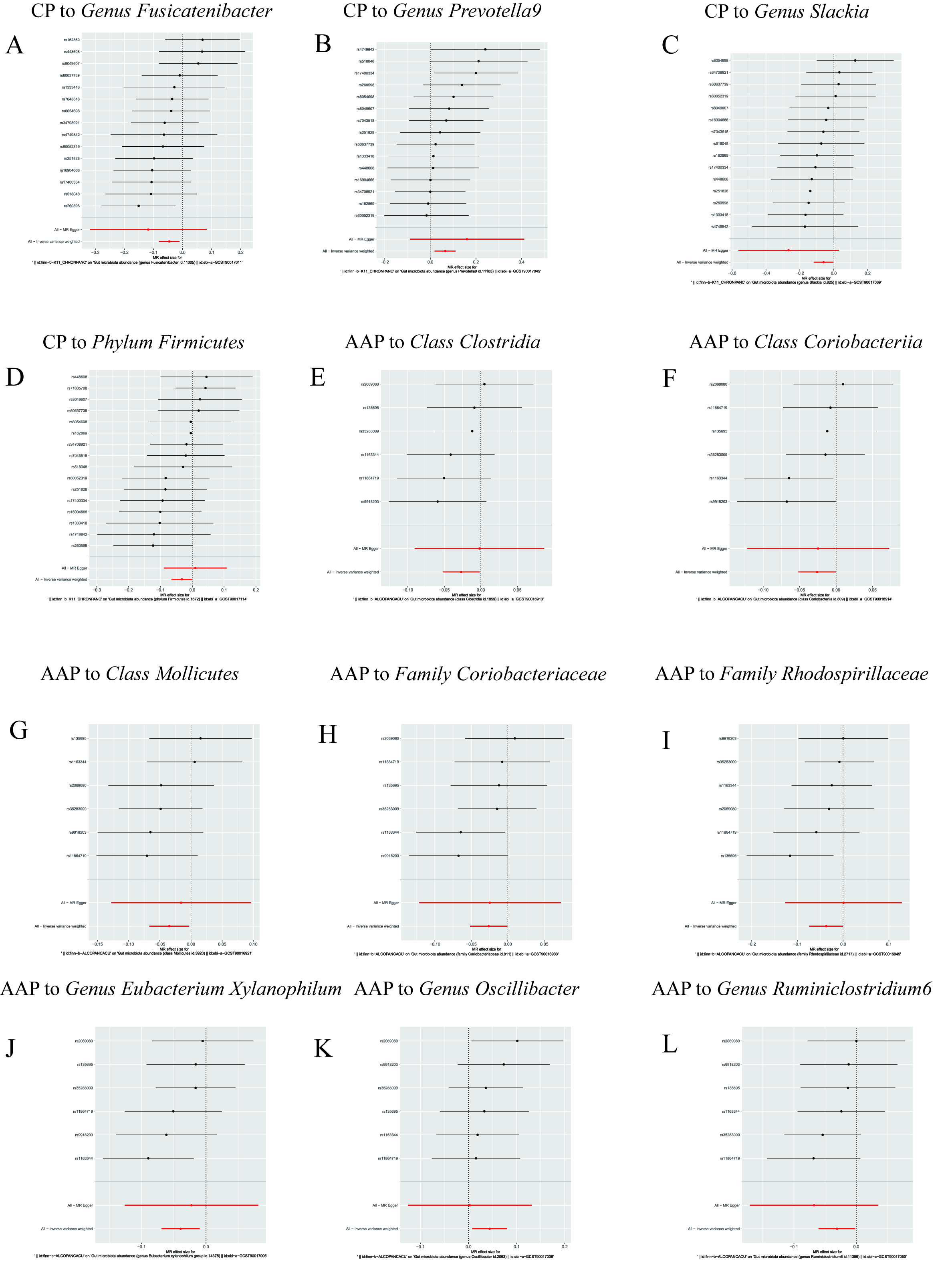

Supplement: Supplementary file 17 [file Image_14.TIF]

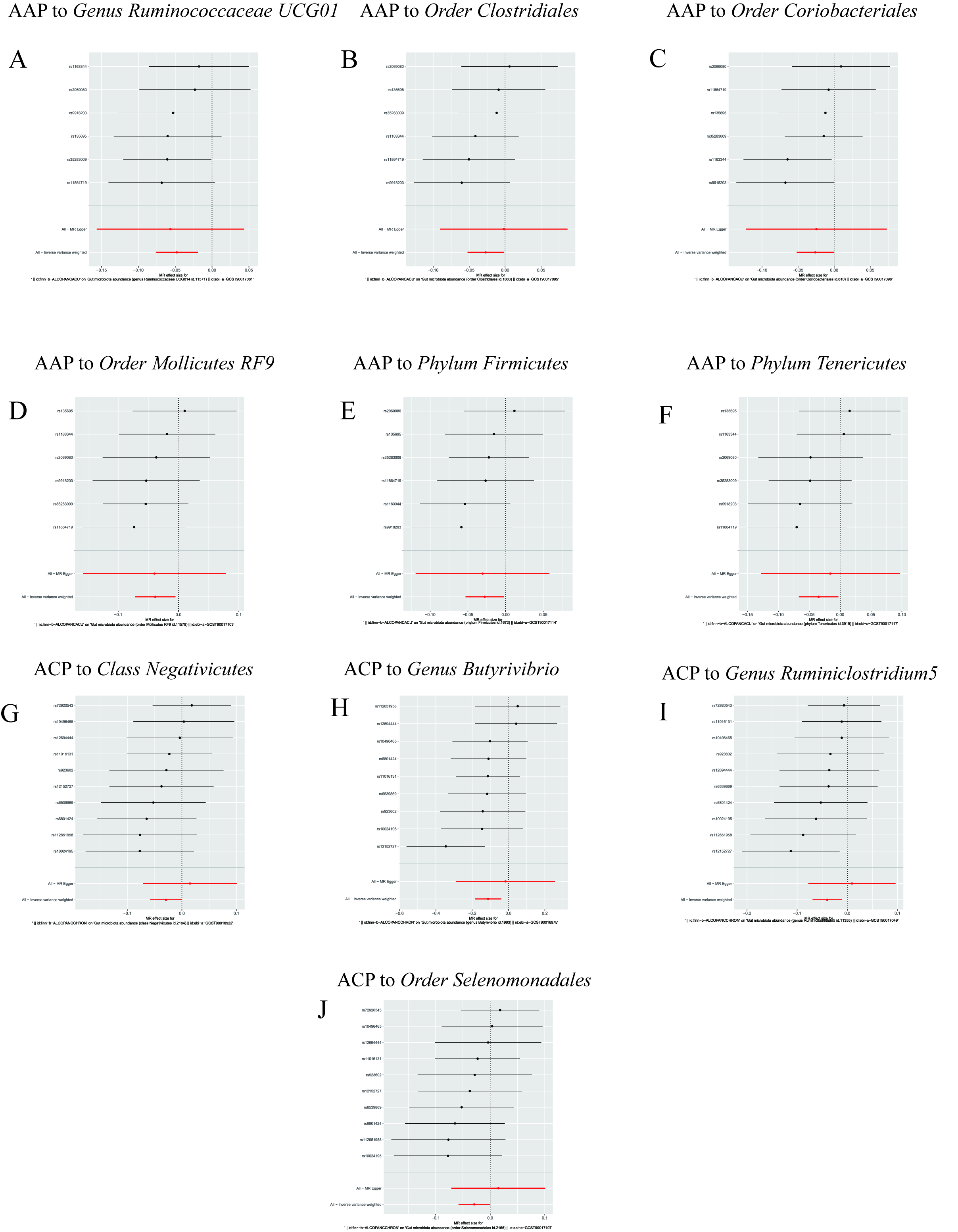

Supplement: Supplementary file 18 [file Image_15.TIF]
